# Ph_3_AsO as a Strong Hydrogen-Bond Acceptor in Cocrystals with Hydrogen Peroxide and *gem-*Dihydroperoxides

**DOI:** 10.1021/acs.inorgchem.4c04535

**Published:** 2025-01-29

**Authors:** Ana Siljanovska, Miha Virant, Matic Lozinšek, Janez Cerkovnik

**Affiliations:** † Faculty of Chemistry and Chemical Technology, University of Ljubljana, Večna pot 113, 1000 Ljubljana, Slovenia; ‡ Jožef Stefan Institute, Jamova cesta 39, 1000 Ljubljana, Slovenia

## Abstract

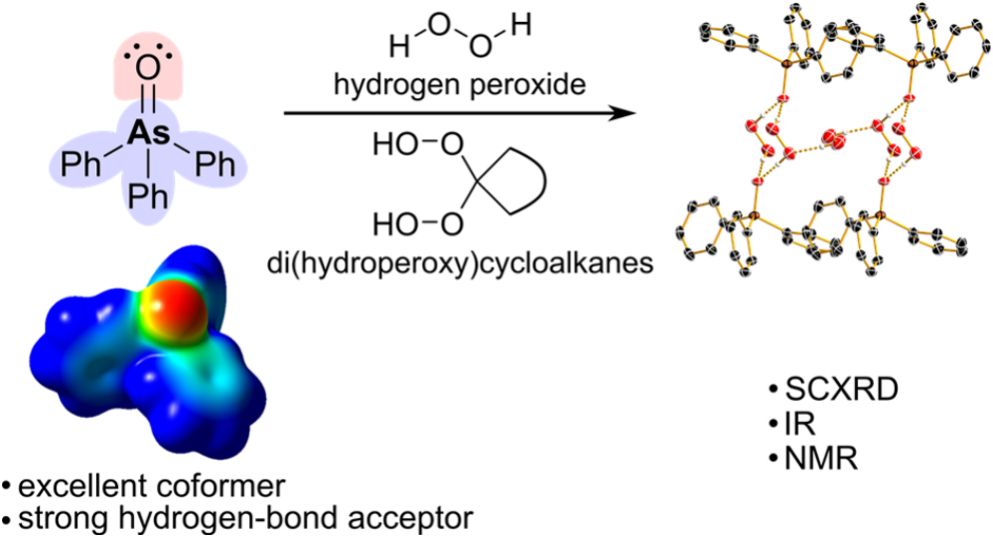

Hydrogen-bonded cocrystals have attracted considerable attention as they allow fine-tuning of properties through the choice of hydrogen-bond donors and acceptors. In this study, triphenylarsine oxide (Ph_3_AsO) is introduced as a strong hydrogen-bond acceptor molecule. Due to its higher Lewis basicity compared to triphenylphosphine oxide (Ph_3_PO), it acts as a strong hydrogen-bond acceptor, which is demonstrated in six new cocrystals with H_2_O_2_ and *gem*-di(hydroperoxy)cycloalkanes. All cocrystals formed large, high-quality single crystals, which were analyzed by X-ray diffraction and IR spectroscopy. The cocrystal of Ph_3_AsO with H_2_O_2_ shows prolonged stability without loss of oxidative power. In addition, the newly formed interactions were also present in solution and were detected by NMR spectroscopy. The higher electron-donating ability of Ph_3_AsO compared to Ph_3_PO was confirmed by competition experiments. Ph_3_AsO exclusively binds H_2_O_2_, even in dilute aqueous solutions and in the presence of Ph_3_PO. This study expands the range of hydrogen-bond acceptors and demonstrates that Ph_3_AsO is a useful cocrystallizing tool in crystal engineering and a sensitive marker for hydrogen peroxide.

## Abstract 2

Triphenylarsine oxide is introduced as a strong hydrogen-bond acceptor and efficient coformer, readily forming cocrystals with H_2_O_2_ and di(hydroperoxy)cycloalkanes. Compared to its phosphorus analog it forms stronger hydrogen bonds with H_2_O_2_ even in dilute aqueous solutions and could serve as a sensitive marker for H_2_O_2_. The obtained cocrystals exhibit improved stability over time.

## Introduction

Crystal engineering has emerged as a powerful strategy in the design of new supramolecular assemblies and advanced materials with tailored properties.^1^ It relies heavily on the use of noncovalent interactions such as hydrogen bonds and σ-hole interactions, π–π stacking and van der Waals forces to combine building blocks into new assemblies, i.e., cocrystals. Among these interactions, hydrogen and halogen bonds stand out, mainly due to their strength and directionality, leading to predictable supramolecular synthons and thus enabling rational design of crystal motifs.

Cocrystal formation has been employed in some remarkable applications, namely to modify the reagent state^2^ as well as to stabilize explosive^3^ or unstable^4^ compounds for safer handling and increased shelf life. In addition, crystal engineering has been used extensively in the development of pharmaceutical cocrystals to improve the physicochemical properties of active pharmaceutical ingredients.^5,6^ Owing to the structure of most biologically active compounds, hydrogen bonding is at the heart of new drug development.^7,8^ The broad applicability of hydrogen bonding is due in part to the versatility of hydrogen-bond donors and acceptors. Acceptor molecules act as Lewis bases and contribute an electron pair to the hydrogen-bond donor. A prototypical example of a Lewis base is a bound oxygen atom with two electron pairs. Electron donation is more pronounced in the case of a strongly polarized bond. While triphenylphosphine oxide (Ph_3_PO) has proven to be an excellent coformer,^9^ the hydrogen-bonding ability and cocrystal formation of its heavier congener triphenylarsine oxide (Ph_3_AsO), which features a highly polarized As=O bond,^10^ was not systematically studied.

**Figure 1:**
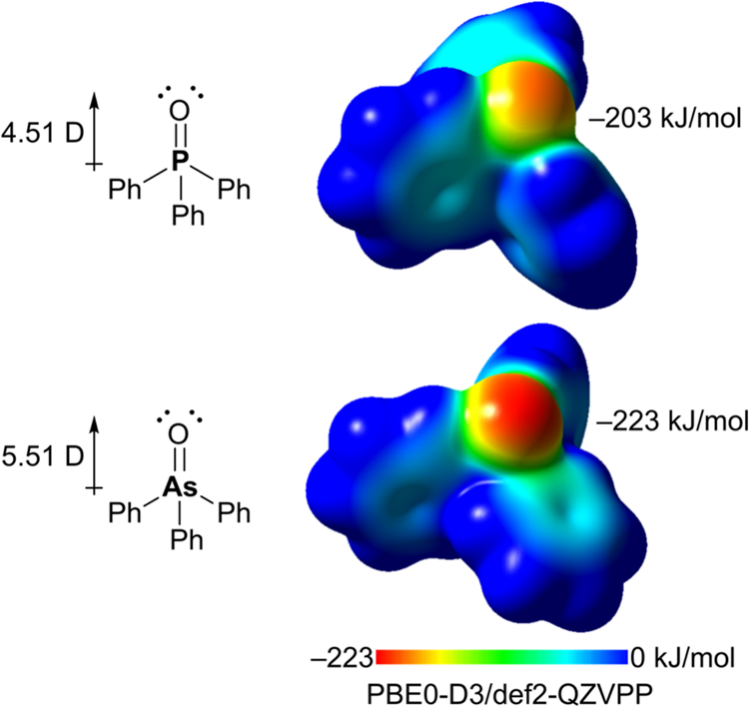
Comparison of the dipole moments and molecular electrostatic potential surfaces (isovalue of 0.001 au) of Ph_3_PO and Ph_3_AsO. Only the negative portion of the electrostatic potential is displayed to better visualize the negatively polarized atoms.

Comparison of the dipole moment of Ph_3_PO (4.51 D, Figure 1)^11^ with its arsenic analog Ph_3_AsO (5.51 D) reveals a more polarized oxygen atom with higher electron density in Ph_3_AsO, which is reflected in its higher basicity (p*K*_aH_ = −2.10, Ph_3_PO; 0.99, Ph_3_AsO).^9,12^ Thus, Ph_3_AsO is a stronger Lewis base compared to its phosphorus analog and is expected to form stronger hydrogen bonds.

In this work, a systematic structural investigation of the hydrogen-bonding propensity of Ph_3_AsO was performed using H_2_O_2_ and *gem*-di(hydroperoxy)cycloalkanes (dhp), which have been previously extensively studied in cocrystals with R_3_PO.^4,13−20^ In addition to structural analysis by single-crystal X-ray diffraction (SCXRD) and IR spectroscopic studies of the solids, the hydrogen-bonded adducts were also investigated in solution by NMR spectroscopy. Furthermore, competition experiments with Ph_3_PO were conducted to compare their electron-donating abilities.

## Results and Discussion

### Preparation of Ph_3_AsO

**Figure 2:**
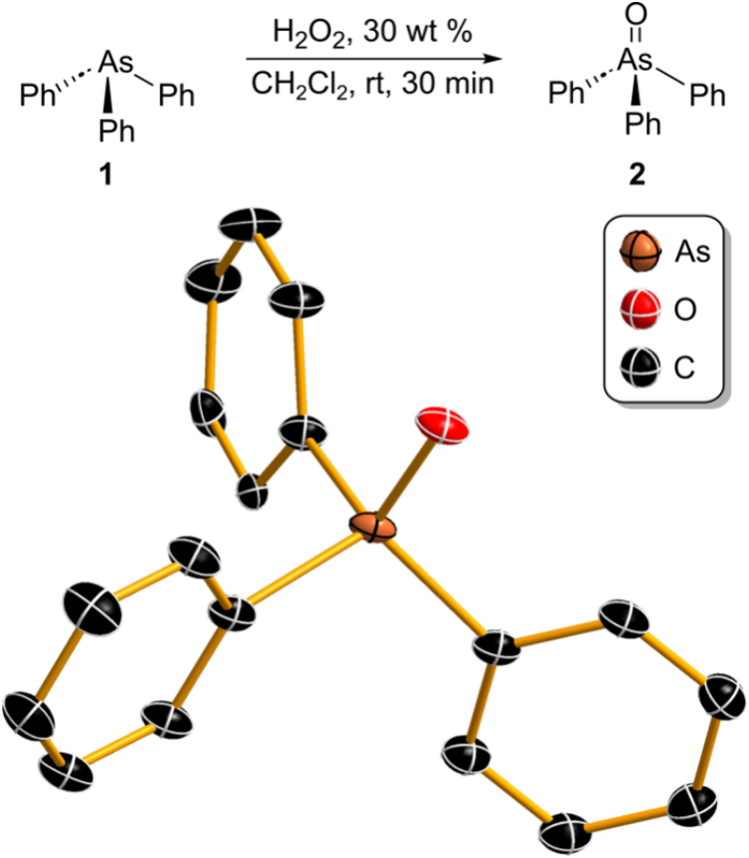
Synthesis and crystal structure of Ph_3_AsO redetermined at low temperature. Thermal ellipsoids are shown at 50% probability and hydrogen atoms are omitted for clarity.

Triphenylarsine, Ph_3_As (**1**), is inert to oxidation even with prolonged exposure to oxygen and heat, similar to triphenylphosphine.^21^ Therefore, the synthesis of triphenylarsine oxide often requires the use of metal reagents.^22−24^ Hydrogen peroxide, a mild and environmentally friendly oxidizing agent, has been used in several examples.^22,25−28^ Existing synthetic protocols sometimes require long reaction times, high temperatures and solvents with high boiling points, as well as the need to further purify the obtained products. A modified, efficient procedure was developed that yields pure Ph_3_AsO after only 30 min with simple extraction (Figure 2). The reaction was also carried out on a multigram scale to illustrate the scalability of the process (see SI).

Slow evaporation of a concentrated CH_2_Cl_2_ solution afforded single crystals suitable for SCXRD analysis. The crystal structure was redetermined^29^ at 100 K (Figure 2) and features molecules of Ph_3_AsO stacked on top of each other. The orientation of the phenyl rings in the solid state is influenced by the weak interactions between the *ortho-* hydrogens and the oxygen of another Ph_3_AsO molecule at an average distance of 2.51 Å. This results in the phenyl rings being virtually parallel to the As=O bond, which is rarely observed in the crystal structures containing Ph_3_AsO (see SI).^30,31^

### Cocrystal of Ph_3_AsO with H_2_O_2_

The formation of adducts from Ph_3_AsO and H_2_O_2_ in either 2:1 or 1:1 ratio has been discussed in previous studies.^32,33^ Their composition and structures were proposed based on observed melting point changes, IR spectroscopy, H_2_O_2_ titration and chemical analysis, which limited the structural information and conclusions derived. To the best of our knowledge, no crystal structure has yet been reported. This provided an additional incentive to investigate the solid-state structures of these systems.

**Figure 3:**
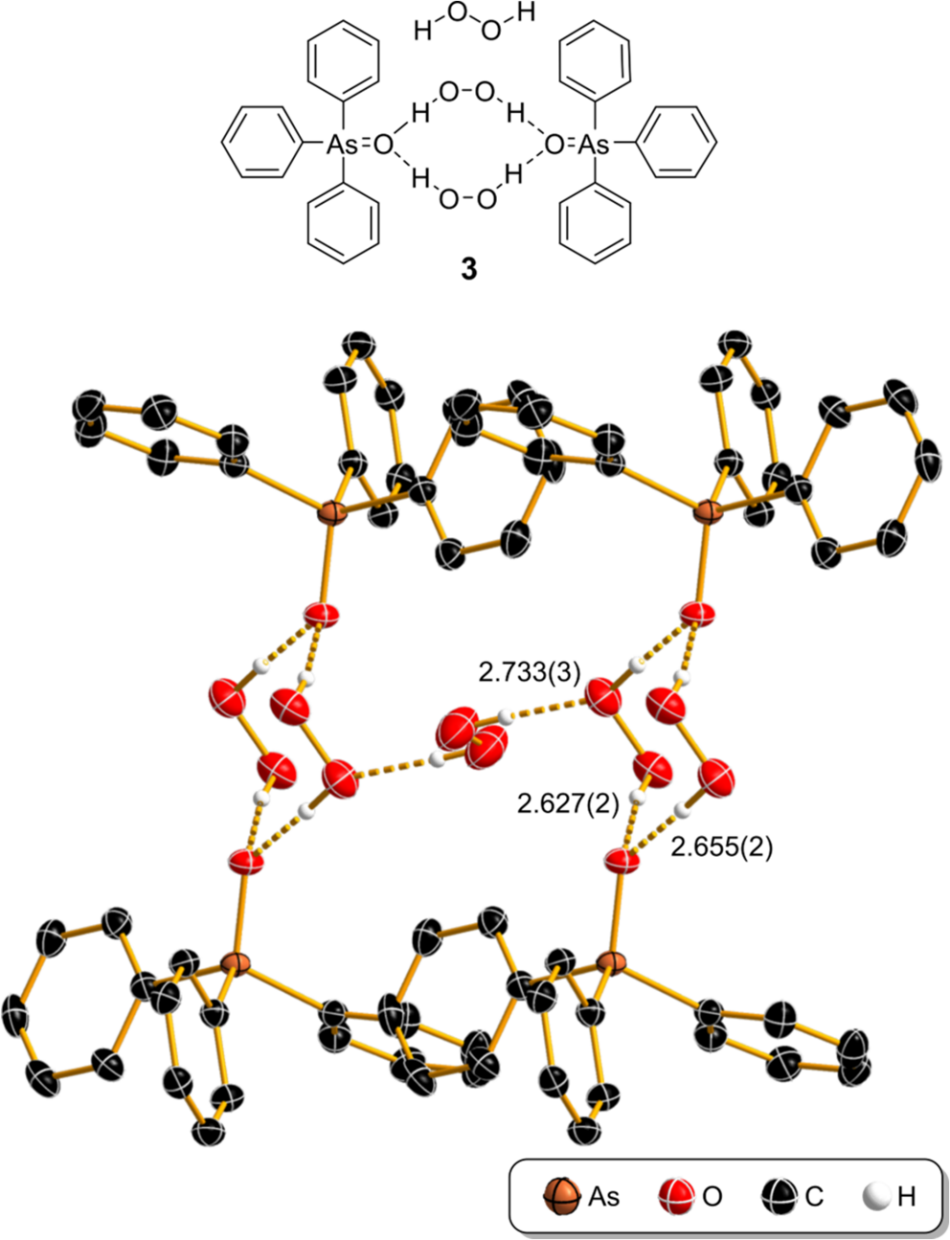
Structure of [Ph_3_AsO·H_2_O_2_]_2_·H_2_O_2_ (**3**) determined by SCXRD. The O···O hydrogen-bond distances are provided in Å. Thermal ellipsoids are shown at 50% probability and hydrogen atoms as spheres of arbitrary radius. Hydrogen atoms not involved in hydrogen bonds are omitted for clarity.

When triphenylarsine oxide (**2**) was mixed with neat H_2_O_2_ in CH_2_Cl_2_ and the solvent slowly evaporated, large colorless single-crystal plates formed over the course of an hour. SCXRD analysis revealed a hydrogen-bonded polymeric structure of [Ph_3_AsO·H_2_O_2_]_2_·H_2_O_2_ (**3**, Figure 3 and SI). It features two H_2_O_2_ molecules that are hydrogen-bonded to two oxygen atoms of arsine oxide **2**. The six oxygen atoms together form a cyclic chair conformation (*D*_3d_-like). Accordingly, Ph_3_AsO is a bifurcated hydrogen-bond acceptor and the hydrogen-bonded ring motif can be described with the graph set notation *R*_4_^2^(10).^34^ These dimers of [Ph_3_AsO·H_2_O_2_]_2_ are connected by an additional “bridging” H_2_O_2_ molecule. The cocrystal **3** is isostructural with the phosphorus analog^4^ [Ph_3_PO·H_2_O_2_]_2_·H_2_O_2_ (Cambridge Structural Database (CSD)^30^ reference code: BAFJUQ).

The hydrogen bonds between H_2_O_2_ and Ph_3_AsO in the [Ph_3_AsO·H_2_O_2_]_2_ dimeric unit are similar, with the O···O distances of 2.627(2) Å and 2.655(2) Å (O···H distances of 1.69(3) Å and 1.71(3) Å) and H_2_O_2_ exhibits an almost perpendicular torsion angle of 84(3)°, slightly smaller than in the native H_2_O_2_ (93(2)°).^35^ The O–O bond of this peroxide molecule is slightly shorter (1.429(2) Å) than the bond distances observed in the crystal structure of pure H_2_O_2_ (1.461(3) Å)^35^ or the average of the CSD reports (1.45 Å, see SI).^30,31^ The quality of the data allowed the free refinement of all hydrogen atoms; the detailed hydrogen-bond parameters are listed in the SI.

The bridging H_2_O_2_ lies on the inversion center with the middle of the O–O bond and is thus interlocked in a planar geometry with a torsion angle of 180° and symmetric hydrogen bonds with O···O and O···H distances of 2.733(3) and 1.86(4) Å, respectively. It exhibits an O–O bond length of 1.464(4) Å, which is within the experimental error of pure H_2_O_2_, despite the torsion angle of 180° being highly distorted from 93(2)° in pure hydrogen peroxide.^35^

### Cocrystals of Ph_3_AsO with *gem*-di(hydroperoxy)cycloalkanes

**Figure 4:**
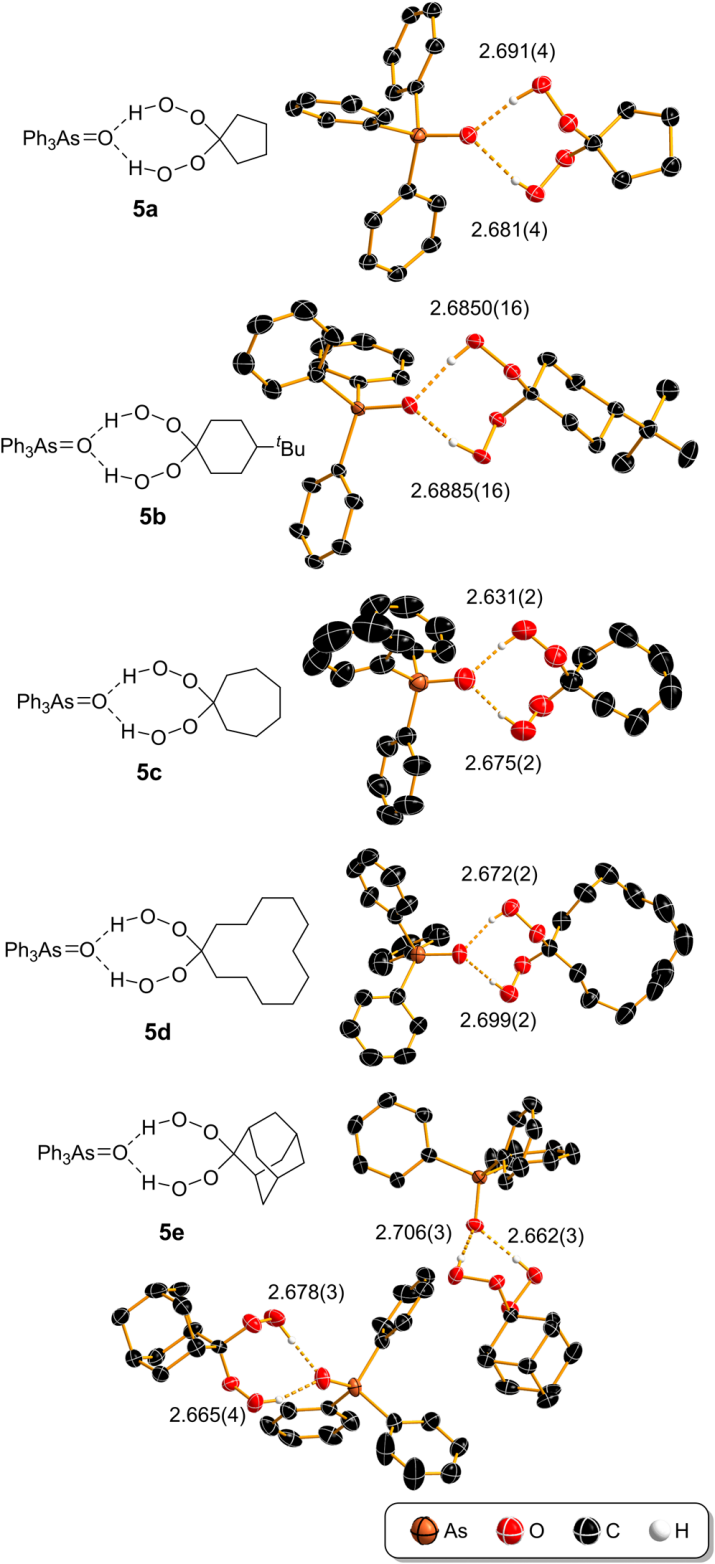
Structures of prepared cocrystals **5a**–**e**. The cocrystal **5e** consists of two hydrogen-bonded adducts in the asymmetric unit (for details see SI). The O···O distances of hydrogen bonds are provided in Å. Thermal ellipsoids are shown at 50% probability and hydrogen atoms as spheres of arbitrary radius. Hydrogen atoms not involved in hydrogen bonds are omitted for clarity.

To further explore the ability of Ph_3_AsO to form hydrogen-bonded cocrystals, a selection of *gem*-di(hydroperoxy)cycloalkanes (dhp, **4a**–**e**, see SI) were used as coformers. These can be readily obtained following the literature procedures (see SI)^36−38^ and have been previously extensively studied in cocrystals with Ph_3_PO.^4,13−20^ Crystallizations of Ph_3_AsO (**2**) and selected dhp (**4a**–**e**) in an equimolar ratio yielded five new hydrogen-bonded cocrystals **5a**–**e** (Figure 4, for preparation see SI).

SCXRD analysis of a series of Ph_3_AsO·dhp cocrystals **5** revealed that both hydroperoxy groups bind to the same Ph_3_AsO molecule, forming the same robust supramolecular synthon in all examples (Figure 4). Ph_3_AsO again acts as a bifurcated hydrogen-bond acceptor, and forms hydrogen-bond ring structures, represented by the graph set notation *R*_2_^1^(8).^34^ Like their phosphorus analogs,^4,13,16,17,20^ all cocrystals **5** contain two active oxygen atoms per AsO group (see SI). Analogous to the dhp cocrystals of Ph_3_PO,^13,20^ the O–C–O angle in the *gem*-di(hydroperoxy)cycloalkane moiety deviates slightly from the ideal tetrahedral angle (110.0 to 111.5°). Structure refinement details are available in the SI.

Hydrogen bonding of two HOO moieties to the oxygen atom of Ph_3_AsO results in the slight elongation of the As=O bond (0.004–0.012 Å), while the largest elongation can be observed in the case of H_2_O_2_ cocrystal **3** (0.014 Å). In cocrystals **5**, the O–O bond lengths, ranging from 1.447(14) to 1.477(4) Å, agree well with the average distances of the CO–OH fragments reported in CSD (1.46 Å, see SI).^30,31^ Structure **5b** represents the first solid-state structure of the 4-(*tert*-butyl)-1,1-dihydroperoxycyclohexane moiety.

The O···O hydrogen-bond distances in cocrystals of Ph_3_AsO with hydrogen peroxide (**3**) and dhp (**5**) are typically smaller than 2.68 Å (see SI) and imply relatively strong hydrogen bonds (0.3 Å shorter than the sum of the vdW radii,^39^*r*_O_ = 1.50 Å).^40,41^ For comparison, the phosphorus analogs have longer hydrogen bonds, i.e., the comparable O···O lengths are 0.05 Å longer in [Ph_3_PO·H_2_O_2_]_2_·H_2_O_2_,^4^ and in R_3_PO·dhp cocrystals they are typically longer than 2.72 Å.^4,13^ This is comparable to the hydrogen bonds formed by the H_2_O_2_ molecule that bridges the hydrogen-bonded dimers [Ph_3_AsO·H_2_O_2_]_2_ in **3**, indicating weaker hydrogen bonds.

### IR Spectroscopy

**Figure 5:**
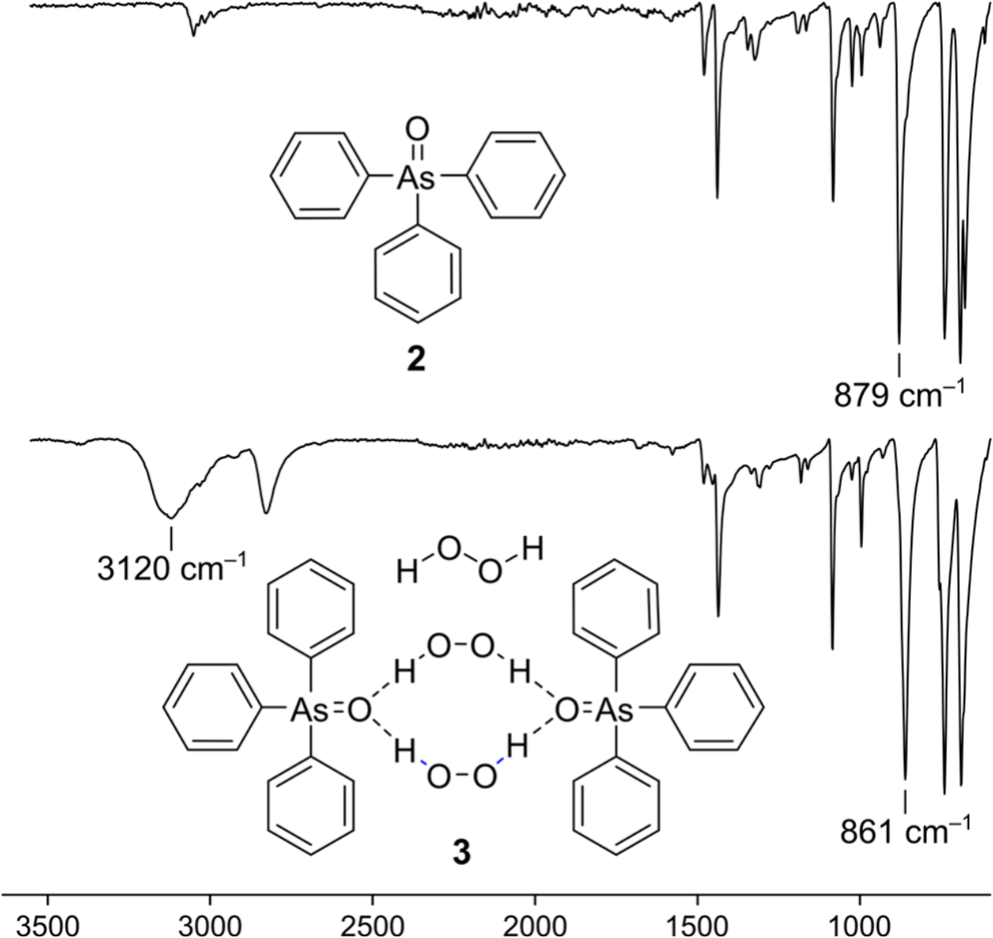
IR spectra of **2** and **3** with marked characteristic As=O and O–H bands.

Hydrogen bonding and cocrystal formation can also be observed by IR spectroscopy, which provides additional insights into the strength and nature of the interactions (Figure 5 and Table 1). The observed ν_As=O_ in **3** (861 cm^–1^) agrees well with the previously reported value (862 cm^–1^)^33^ and is significantly red-shifted in comparison to the ν_As=O_ in pure Ph_3_AsO (879 cm^–1^), which is consistent with the weakening of the As=O bond due to hydrogen bonding. Similarly, the stretching frequencies for the As=O bond in the hydrogen-bonded cocrystals **5a**–**e** (863–872 cm^–1^) are also shifted to lower wavenumbers by 7–16 cm^–1^ (Table 1). In addition, the O–H stretching vibration of hydrogen peroxide in cocrystal **3** (Figure 5, Table 1) is significantly red-shifted (3120 cm^–1^) in comparison to the value observed in neat H_2_O_2_ (3400 cm^–1^)^42^ and slightly lower than the value for the previously reported 1:1 adduct (3150 cm^–1^).^33^ Noteworthy is the stretching vibration at 2829 cm^–1^, which can be assigned to a combination or overtone frequency of O–H deformation vibrations, intensified due to Fermi resonance with the O–H fundamental vibration.^33^ The ν_O–H_ of the hydrogen-bonded OOH moieties of the Ph_3_AsO·dhp cocrystals **5a**–**e** also appear at lower frequencies (3112–3159 cm^–1^) than in the pure dhp compounds (Table 1).^43^

**Table 1:** As=O and O–H IR Stretching Frequencies (cm^–1^) of the Hydrogen-bonded Cocrystals **3** and **5a**–**e**

cocrystal	ν_As=O_	Δν_As__=__O_^a^	ν_O–H_	Δν_O–H_^b^
**3**	861	–18	3120	–280^c^
**5a**	867	–12	3149	–271^d^
**5b**	872	–7	3159	–241
**5c**	868	–11	3112	–300
**5d**	867	–12	3122	–293
**5e**	863	–16	3148	–281

a The difference between the As=O stretch of the studied cocrystals and pure Ph_3_AsO (879 cm^–1^).

b The difference between the O–H stretch of hydrogen-bonded Ph_3_AsO·dhp cocrystals **5b**–**e** and pure dhp compounds.^43^

c The difference between the O–H stretch of **3** and neat H_2_O_2_ (3400 cm^–1^).^42^

d The difference between the O–H stretch of hydrogen-bonded Ph_3_AsO·dhp cocrystal **5a** and pure dhp compound.^100^

The differences between the remaining frequencies of free Ph_3_AsO and those of the corresponding cocrystals are only slight. No clear trend can be observed for the dependence of the stretching frequencies on the ring size and the flexibility of the dhp moiety. A comparison of ν_O–H_ with the reported values for Ph_3_PO·dhp cocrystals^15,17^ shows that Ph_3_AsO is indeed a stronger hydrogen-bond acceptor than Ph_3_PO. This is reflected in the lower O–H stretching frequency of **5e** (3148 cm^–1^), compared to that of the phosphorus analog (3269 cm^–1^)^17^ and the corresponding pure dhp **4e** (3429 cm^–1^).^43^

### Investigation of Hydrogen Bonding in Solution

**Figure 6:**
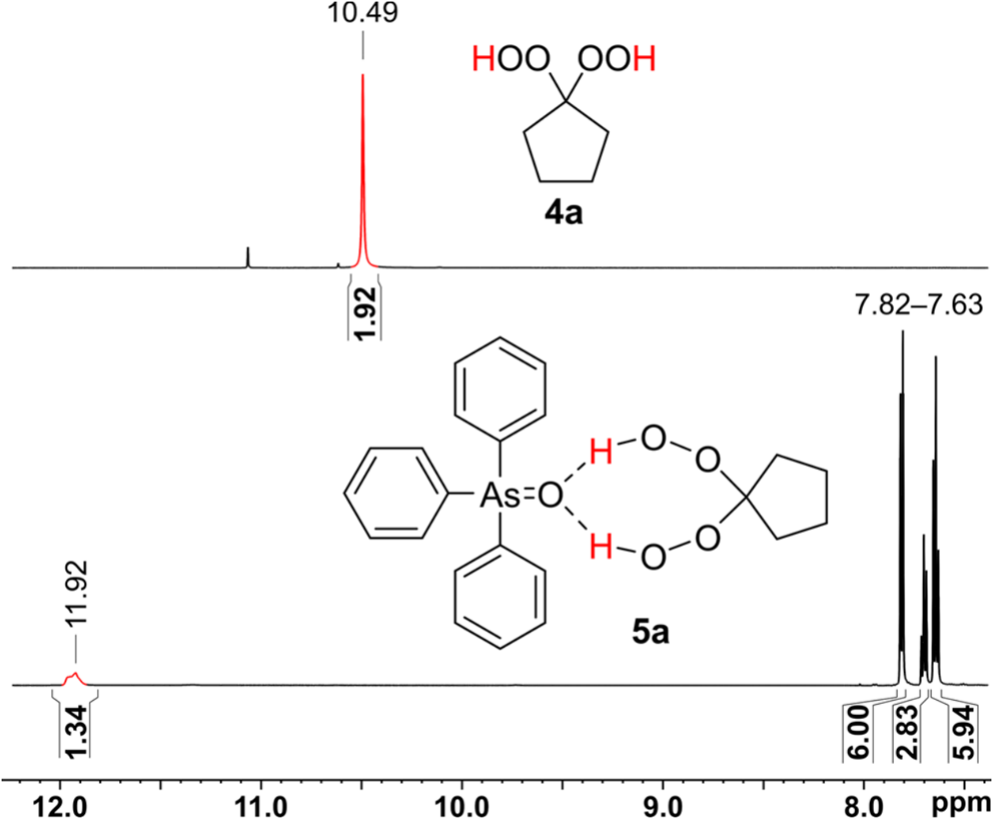
Section of ^1^H NMR spectra for the OOH protons of **4a** (top) and after addition of Ph_3_AsO and formation of hydrogen bonds (bottom) in acetone-*d*_6_.

The formation of hydrogen-bonded adducts of Ph_3_AsO with hydrogen peroxide and dhp was also observed by ^1^H NMR spectroscopy. The hydroperoxy ^1^H NMR resonance shifts significantly downfield upon adduct formation, i.e., δ(OOH): 10.49 ppm for **4a**, and 11.92 ppm for **5a** in acetone-*d*_6_ (Figure 6 and SI). The resonances for the phenyl rings of Ph_3_AsO as well as the resonances for the alkyl protons of **4**, and all carbon resonances far from the hydroperoxy group remain unaffected (see SI).

The cocrystals exhibit high solubility in polar organic solvents, such as MeOH and CH_2_Cl_2_ and are insoluble in water and hexane (see SI).

To gain some insight into the behavior in solution, the persistence of the dimeric structure of [Ph_3_AsO·H_2_O_2_]_2_ in solution was evaluated by DFT (for detailed description of the computational methods see SI). The results suggest that formation of the dimeric species [Ph_3_AsO·H_2_O_2_]_2_ in acetone is slightly endergonic. The monomeric form Ph_3_AsO·H_2_O_2_ is somewhat more stable. On the other hand, adduct formation with dhp **4a** is exergonic in the case of Ph_3_AsO (see SI). Comparison with the phosphorus analogs further corroborates the formation of stronger hydrogen bonds with Ph_3_AsO.

### Competition Experiments

To test the affinity of Ph_3_AsO toward water as a competing hydrogen-bond donor in H_2_O_2_ solutions, the preparation of **3** was also carried out with 30% and 10% aqueous solutions of H_2_O_2_. In both cases, only the formation of product **3** was confirmed, as indicated by IR analysis of the bulk samples (see SI). This indicates that Ph_3_AsO as a hydrogen-bond acceptor preferentially forms hydrogen bonds with H_2_O_2_ even in large excess of H_2_O. Furthermore, this enabled the development of a simplified one-pot synthesis of **3** from Ph_3_As (**1**) and a commercially available H_2_O_2_ solution (see SI).

**Figure 7:**
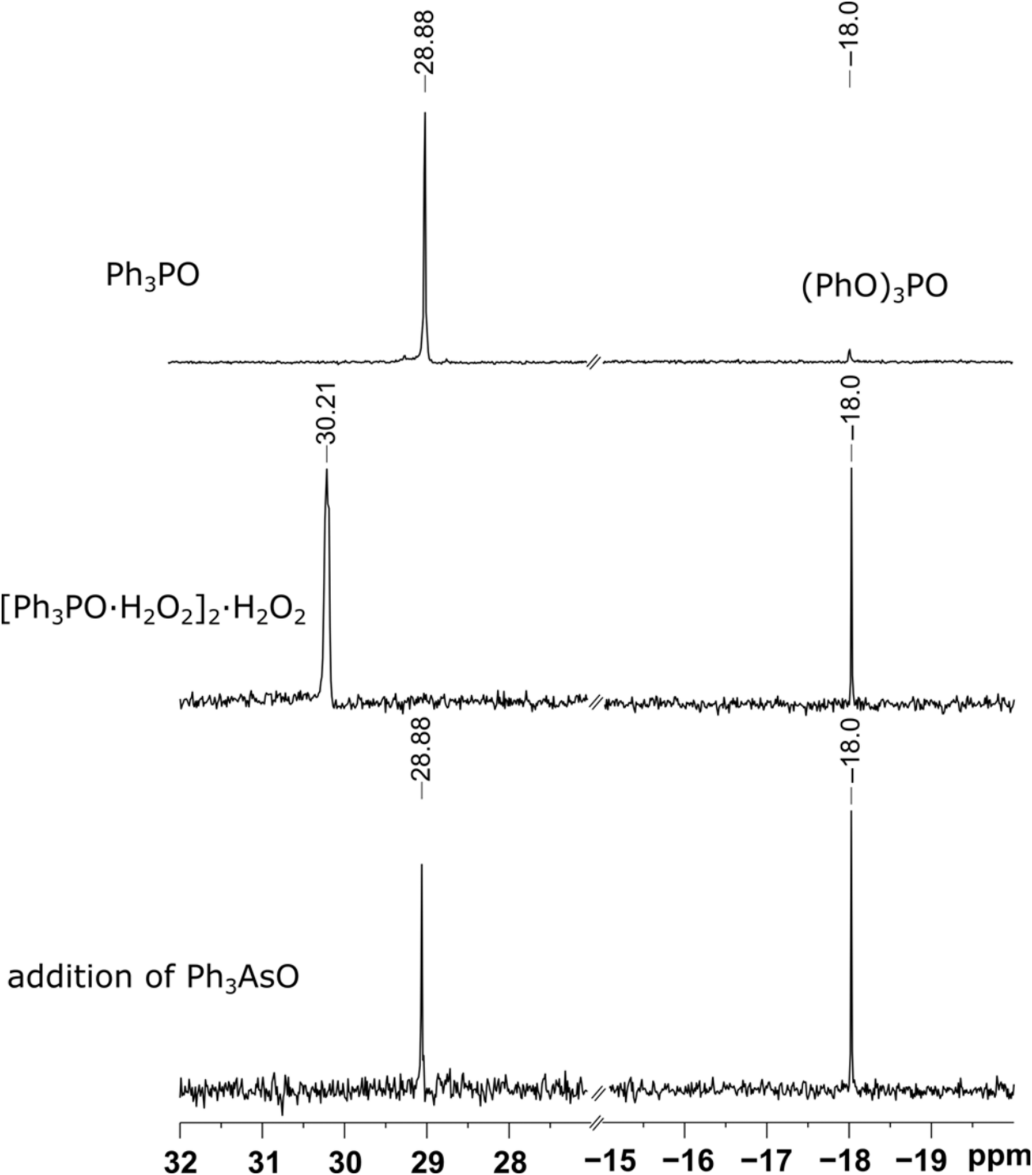
^31^P NMR spectra recorded in CDCl_3_: pure Ph_3_PO (top); solution of [Ph_3_PO·H_2_O_2_]_2_·H_2_O_2_ (middle), and the solution of [Ph_3_PO·H_2_O_2_]_2_·H_2_O_2_ after addition of 1 eq. of Ph_3_AsO (bottom). (PhO)_3_PO in a capillary was used as an external standard.

To further probe the hydrogen-bond acceptor ability of Ph_3_AsO in comparison with Ph_3_PO, a competition experiment was performed. Cocrystals of Ph_3_PO with H_2_O_2_ were prepared according to the previously described procedure (see SI).^4^ The ^31^P NMR chemical shift of [Ph_3_PO·H_2_O_2_]_2_·H_2_O_2_ dissolved in CDCl_3_ differs from free Ph_3_PO by 1.33 ppm. Upon addition of 1 equiv of Ph_3_AsO, the ^31^P resonance shifts back to the value of free Ph_3_PO, indicating the preferential formation of hydrogen bonds with the more basic hydrogen-bond acceptor Ph_3_AsO (Figure 7 and SI). Moreover, when equimolar amounts of Ph_3_PO, Ph_3_AsO and H_2_O_2_ (30 wt %) were combined in CH_2_Cl_2_, ^31^P NMR showed only free Ph_3_PO, suggesting that H_2_O_2_ is hydrogen-bonded solely to a stronger base Ph_3_AsO.

### Stability of [Ph_3_AsO·H_2_O_2_]_2_·H_2_O_2_

The loss of oxidative power of [Ph_3_AsO·H_2_O_2_]_2_·H_2_O_2_ (**3**) was investigated as previously described.^4^ To this end, a polycrystalline sample of **3** was stored at 5 °C and tested in the oxidation reaction with Ph_3_P weekly by ^1^H and ^31^P NMR. The oxidation is fast and selective and was carried out with a slight excess of Ph_3_P, which allowed the calculation of the conversion. After 30 min of reaction time, pure Ph_3_AsO was the only byproduct, and no residual hydrogen peroxide was visible in the ^1^H NMR spectrum. The oxidizing power of **3** remained virtually unchanged for several weeks, indicating that it retains its oxidizing power and is stable for at least two months (see SI). In addition, the thermal stability of the hydrogen-bonded adduct **3** in solution was investigated by heating a toluene solution at 70 °C. Aliquots of this solution were analyzed hourly by ^1^H NMR. Complete loss of H_2_O_2_ was only observed after 24 h, suggesting that the Ph_3_AsO adduct exhibits some resistance to rapid decomposition in solution even at elevated temperatures. In comparison, the phosphorus congener showed a lesser degree of stability under the same conditions, with complete decomposition taking place after 16 h. The stability of **3** together with its solubility in organic solvents and its defined stoichiometry suggests that it could be used as a convenient source of H_2_O_2_ in organic solvents.

## Experimental Section

**Caution!** Although we have encountered no accidents with organic peroxides, care should be exercised when handling these potentially hazardous (explosive) compounds. Acetone solutions of H_2_O_2_ should be handled carefully and disposed of immediately after use.^44^ Additionally, standard precautionary measures should be applied when handling arsenic compounds due to their potential toxicity.

### Synthesis of Ph_3_AsO (**2**)

In a 25 mL round-bottom flask Ph_3_As (**1**, 306.2 mg, 1 mmol, 1 equiv) was weighed and dissolved in 3 mL of CH_2_Cl_2_. Hydrogen peroxide (511 μL, 5 mmol, 5 equiv, 30% aqueous solution) was added dropwise while stirring vigorously. After a few minutes of stirring a spontaneous warming up of the reaction mixture could be observed. After stirring for 30 min the reaction was quenched by addition of a saturated NaHCO_3_ solution and 5% solution of Na_2_S_2_O_3_ and stirred for an additional few minutes. The phases were separated, and the organic layer was washed 2 more times with 5% solution of Na_2_S_2_O_3_. The combined organic layers were dried over anhydrous Na_2_SO_4_. After evaporation of the solvent a white solid was obtained (**2**, 312.1 mg, 97%).

#### ^1^H (600 MHz, acetone-*d*_6_)

δ 7.81–7.76 (m, 6H), 7.66–7.62 (m, 3H), 7.61–7.57 (m, 6H).

### Synthesis of [Ph_3_AsO·H_2_O_2_]_2_·H_2_O_2_ (**3**)

In a 25 mL round-bottom flask Ph_3_AsO (**2**, 524.4 mg, 1.63 mmol, 1 equiv) and neat H_2_O_2_ (191 μL, 8.15 mmol, 5 equiv) were dissolved in 5 mL of CH_2_Cl_2_. After slow evaporation of the solvent colorless plates of **3** formed, which were collected on a glass frit and thoroughly washed with hexane (608 mg, 98%).

#### ^1^H (600 MHz, acetone-*d*_6_)

δ 10.18 (br, *H*_*2*_O_2_), 7.81–7.76 (m, 6H), 7.66–7.62 (m, 3H), 7.61–7.57 (m, 6H).

### General Procedure for the Synthesis of Cocrystals **5a**–**e**

In a 50 mL round-bottom flask equimolar amounts of Ph_3_AsO (**2**) and dhp (**4a**–**e**) were dissolved in 5 mL of CH_2_Cl_2_. Hexane (5 mL) was added and the reaction mixture was slowly concentrated under vacuum at room temperature. When a cloudy white solid began forming, the evaporation was stopped, and the flask was left for additional 15 min in the fumehood. After that colorless crystals of the desired products were collected. Spectroscopic and crystallographic data for cocrystals **5a**–**e** are available in the Supporting Information.

## Conclusions

This work represents the first systematic structural investigation of the hydrogen-bonding propensity of Ph_3_AsO. The newly obtained hydrogen-bonded cocrystals were characterized by SCXRD and IR spectroscopy in the solid state and the hydrogen bonding was studied by NMR in solution. The [Ph_3_AsO·H_2_O_2_]_2_·H_2_O_2_ cocrystals (**3**) form chair-like dimers with an additional bridging H_2_O_2_ molecule. In addition, five new Ph_3_AsO·dhp cocrystals were prepared and fully characterized. Single-crystal X-ray diffraction shows that all cocrystals are held together by relatively short hydrogen bonds. This is reflected in the consistent formation of **3** even from very dilute aqueous solutions of H_2_O_2_, which was further exploited in the development of a direct one-pot synthesis of **3** from Ph_3_As. In addition, preferential hydrogen-bond formation tests confirm the higher Lewis basicity of Ph_3_AsO over Ph_3_PO.

By demonstrating that Ph_3_AsO is a strong hydrogen-bond acceptor and an efficient coformer, this study opens a new avenue of research in the field of crystal engineering. When fine-tuning of the acceptor is required, the substituted arsine oxides (R_3_AsO) could be considered in addition to substituted phosphine oxides (R_3_PO), thus considerably enriching the selection of available coformers. This can be particularly useful in crystal engineering of cocrystals with hydrogen and halogen bonds. Finally, Ph_3_AsO could potentially be used as a sensitive marker for hydrogen peroxide due to its ability to preferentially form hydrogen bonds with H_2_O_2_.

## Supporting Information Available

Additional experimental details, materials, and methods, details of X-ray crystallography including, crystal packing, selected bond distances and angles, hydrogen-bonding parameters, DFT calculations, and CSD analyses (PDF)
