# Supplementary material for: Ph3AsO as a Strong Hydrogen-Bond Acceptor in Cocrystals with Hydrogen Peroxide and gem-Dihydroperoxides
Source: Inorg Chem. 2025 Jan 29;64(5):2329–35. doi: 10.1021/acs.inorgchem.4c04535 (PMC11815835; doi:10.1021/acs.inorgchem.4c04535)
Supplement: Supplementary file 1 — ic4c04535_si_001.pdf [file ic4c04535_si_001.pdf]

SUPPORTING INFORMATION

Ph_3AsO as a Strong Hydrogen-Bond Acceptor in Cocrystals with Hydrogen Peroxide and *gem*-Dihydroperoxides

Ana Siljanovska,[a] Miha Virant,[b,*] Matic Lozinšek,[b] Janez Cerkovnik[a,*]

[a] Faculty of Chemistry and Chemical Technology, University of Ljubljana, Večna pot 113, 1000 Ljubljana, Slovenia

[b] Jožef Stefan Institute, Jamova cesta 39, 1000 Ljubljana, Slovenia

*Email: janez.cerkovnik@fkkt.uni-lj.si and miha.virant@ijs.si

Table of Contents

GENERAL INFORMATION 4
EXPERIMENTAL PART 5
Synthesis of Ph_3AsO (**2**) 5
Scale-up synthesis of Ph_3AsO (**2**) 5
Synthesis of $[Ph_3AsO \cdot H_2O_2]_2 \cdot H_2O_2$ (**3**) 6
One-pot synthesis of **3** 6
Synthesis of **3** with aqueous solutions of H_2O_2 6
General procedure for synthesis of *gem*-di(hydroperoxy)cycloalkanes **4** 9
1,1-Dihydroperoxycyclopentane (**4a**) 9
4-(*tert*-Butyl)-1,1-dihydroperoxycyclohexane (**4b**) 9
1,1-Dihydroperoxycycloheptane (**4c**) 10
1,1-Dihydroperoxycyclododecane (**4d**) 10
2,2-Dihydroperoxyadamantane (**4e**) 10
General procedure for the synthesis of Ph_3AsO·dhp cocrystals **5** 11
Cocrystal $Ph_3AsO \cdot (HOO)_2(c\text{-}C_5H_8)$ (**5a**) 11
Cocrystal $Ph_3AsO \cdot (HOO)_2(4\text{-}^tBu\text{-}c\text{-}C_6H_9)$ (**5b**) 13
Cocrystal $Ph_3AsO \cdot (HOO)_2(c\text{-}C_7H_{12})$ (**5c**) 14
Cocrystal $Ph_3AsO \cdot (HOO)_2(c\text{-}C_{12}H_{22})$ (**5d**) 16
Cocrystal $Ph_3AsO \cdot (HOO)_2$(adm) (**5e**) 17
Miscellaneous experiments 19
Oxidation of triphenylphosphine and monitoring the oxidative ability of **3** 19
Synthesis of $[Ph_3PO \cdot H_2O_2]_2 \cdot H_2O_2$ 19
Competition tests 20
Solubility of selected adducts 21
CRYSTALLOGRAPHIC PART 22
Summary of the crystal data and structure refinements 23
Ph_3AsO (**2**) 25
$[Ph_3AsO \cdot H_2O_2]_2 \cdot H_2O_2$ (**3**) 30
$Ph_3AsO \cdot (HOO)_2(c\text{-}C_5H_8)$ (**5a**) 35
$Ph_3AsO \cdot (HOO)_2(4\text{-}^tBu\text{-}c\text{-}C_6H_9)$ (**5b**) 39
$Ph_3AsO \cdot (HOO)_2(c\text{-}C_7H_{12})$ (**5c**) 43
$Ph_3AsO \cdot (HOO)_2(c\text{-}C_{12}H_{22})$ (**5d**) 47
$Ph_3AsO \cdot (HOO)_2$(adm) (**5e**) 51
Hydrogen-bond geometry in cocrystals **3** and **5a–e** 56
Selected bond distances and angles 57

Literature reports of H_2O_2 crystal structure....63
Statistics of the O−O bond lengths in the CSD database....64
Orientation of the phenyl groups in Ph_3AsO....66
COMPUTATIONAL PART....71
General information....71
Energetics....71
Optimized structures....72
SUPPORTING REFERENCES....81

GENERAL INFORMATION

All reactions were carried out under ambient conditions, if not stated otherwise. Starting materials were used as obtained from commercial sources without further purification (Merck, Fluorochem, abcr). Aqueous H_2O_2 solution (30 and 60 %), which was obtained from Belinka Perkemija, is without any added stabilizers apart from HNO_3, which is always added in its synthesis. Neat H_2O_2 refers to 100% H_2O_2, which was obtained by azeotropic distillation of a 60% solution with dry acetonitrile.[1] Solvents were used as received and were of technical grade, unless specified otherwise. Anhydrous acetonitrile was dried and purified in MBraun SPS 5 Solvent Purifier. Room temperature refers to 23–25 °C.

NMR spectra were recorded with a Bruker Avance Neo 600 MHz NMR spectrometer operating at 600 MHz (^{1}H) and 150 MHz (^{13}C) at 296 K in acetone-d_6 or a Bruker Avance III 500 MHz NMR instrument operating at 500 MHz (^{1}H) and 202 MHz (^{31}P) at 296 K in $CDCl_3$. Proton spectra were referenced to the residual signal of acetone-d_5 (at δ 2.05 ppm) or $CDCl_3$ (at δ 7.26 ppm). Carbon chemical shifts are given against the central line of the solvent signal of acetone-d_6 (at δ 29.84 and 206.26 ppm). A 0.05 M solution of $(PhO)_3PO$ in $CDCl_3$ (δ(^{31}P) = −18 ppm)[2] in a capillary centered in the 5 mm NMR tubes was used for referencing the ^{31}P chemical shifts of dissolved compounds. ^{31}P NMR spectra were acquired with a Bruker ^{31}P composite pulse decoupling (CPD) program. Assignment of proton and carbon resonances was done using 2D NMR techniques (^{1}H–^{1}H *gs*-COSY and ^{1}H–^{13}C *gs*-HSQC). Chemical shifts are given on δ scale (ppm). Coupling constants (*J*) are given in Hertz. Multiplicities are indicated as follows: s (singlet), d (doublet), t (triplet), q (quartet), m (multiplet), and br (broadened).

IR spectra were obtained with Bruker ALPHA FT-IR spectrometer equipped with a Platinum ATR sampling module. Melting points (mp) were determined on a Leica Galen III micro hot stage and were not corrected.

Single-crystal X-ray diffraction measurements were performed on a Rigaku OD XtaLAB Synergy-S diffractometer equipped with dual Ag/Cu micro-focus source and Dectris EIGER2 R CdTe 1M detector.

Caution! Although we have encountered no accidents with organic peroxides, care should be exercised when handling these potentially hazardous (explosive) compounds. Acetone solutions of H_2O_2 should be handled carefully and disposed of immediately after use.[3] Additionally, standard precautionary measures should be applied when handling arsenic compounds due to their potential toxicity.

EXPERIMENTAL PART

Synthesis of Ph_3AsO (**2**)

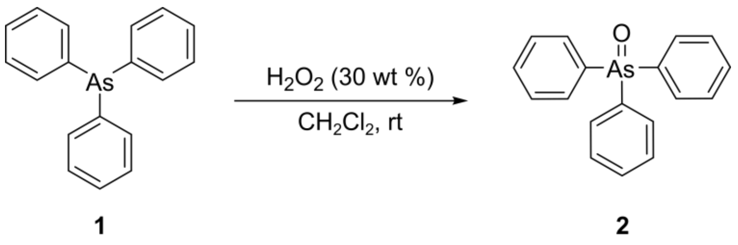

n [mmol]	t [min]	m (**2**) [g]	isolated yield [%]
1	30	0.312	97
25	90	8.42	98

Figure S1. Oxidation of Ph_3As with H_2O_2 on a 1 mmol and 25 mmol scale.

In a 25 mL round bottom flask Ph_3As (**1**, 306.2 mg, 1 mmol, 1 eq.) was weighed and dissolved in 3 mL of CH_2Cl_2. Hydrogen peroxide (511 µL, 5 mmol, 5 eq., 30 wt %) was added dropwise while the solution was vigorously stirred. After a few minutes of stirring a spontaneous warming up of the reaction mixture could be observed. After stirring for 30 min the reaction was quenched by addition of 5 mL of a saturated $NaHCO_3$ solution and 10 mL of 5% solution of $Na_2S_2O_3$ and stirred for an additional few minutes. The phases were separated, and the organic layer was washed 2 more times with 30 mL of a 5% solution of $Na_2S_2O_3$. The combined organic layers were dried over anhydrous Na_2SO_4. After evaporation of the solvent a white solid was obtained (**2**, 312.1 mg, 97%). Slow evaporation of a CH_2Cl_2 solution afforded **2** as colorless crystals suitable for SCXRD analysis.

IR (cm^{-1}): 3051, 1480, 1439, 1324, 1083, 1024, 995, 879, 739, 691.

^{1}H (600 MHz, acetone-*d_6*): δ 7.81–7.76 (m, 6H), 7.66–7.62 (m, 3H), 7.61–7.57 (m, 6H).

^{13}C{^{1}H} (150 MHz, acetone-*d_6*): δ 135.4, 132.7, 132.1, 130.2.

Scale-up synthesis of Ph_3AsO (**2**)

The same procedure as described above was scaled up and performed with Ph_3As (8.174 g, 26.69 mmol, 1 eq.) and H_2O_2 (13.63 mL, 133.5 mmol, 5 eq.) in 80 mL CH_2Cl_2. The reaction time required prolonging to 90 minutes to reach complete conversion. The reaction was quenched by addition of 100 mL of a saturated $NaHCO_3$ solution and 200 mL of 5% solution of $Na_2S_2O_3$ and stirred for an additional few minutes. The phases were separated, and the organic layer was washed 2 more times with 100 mL of a 5% solution of $Na_2S_2O_3$. A white solid was obtained (**2**, 8.42 g, 98%).

Synthesis of $[Ph_3AsO{\cdot}H_2O_2]_2{\cdot}H_2O_2$ (**3**)

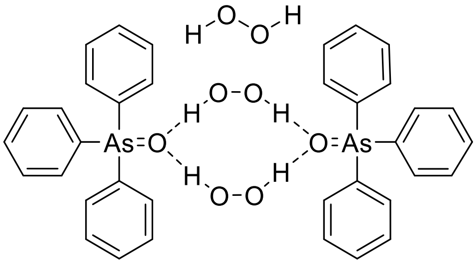

In a 25 mL round bottom flask Ph_3AsO (**2**, 524.4 mg, 1.63 mmol, 1 eq.) and neat H_2O_2 (191 µL, 8.15 mmol, 5 eq.) were dissolved in 5 mL of CH_2Cl_2. After slow evaporation of the solvent colorless plates of **3** were formed, which were collected on a glass frit and thoroughly washed with hexane (608 mg, 98%).

IR (cm^{-1}): 3120, 2829, 1437, 1085, 997, 861, 741, 689.

^{1}H (600 MHz, acetone-*d*$_6$): δ 10.18 (br, H_2O_2), 7.81–7.76 (m, 6H, H-2, H-6), 7.66–7.62 (m, 3H, H-4), 7.61–7.57 (m, 6H, H-3, H-5).

^{13}C{^{1}H} (150 MHz, acetone-*d*$_6$): δ 135.3 (C-1), 132.8 (C-4), 132.1 (C-2, C-6), 130.2 (C-3, C-5).

mp 167.3–172.7 °C

One-pot synthesis of **3**

In a 5 mL round bottom flask Ph_3As (**1**, 41.8 mg, 0.136 mmol, 1 eq.) and neat H_2O_2 (32 µL, 1.36 mmol, 10 eq.) were dissolved in 1 mL of CH_2Cl_2. After slow evaporation of the solvent colorless plates of **3** were formed, which were collected on a glass frit and thoroughly washed with hexane (50 mg, 90%). The spectroscopic data were in agreement with the values reported above.

Synthesis of **3** with aqueous solutions of H_2O_2

In a 5 mL round bottom flask Ph_3AsO (**2**, 41.4 mg, 0.128 mmol, 1 eq.) and H_2O_2 (210 µL, 0.64 mmol, 5 eq., 30 wt %) were mixed in 1 mL of CH_2Cl_2. The phases were separated and after slow evaporation of the solvent colorless plates of **3** formed, which were collected on a glass frit and thoroughly washed with hexane (42 mg, 88%). The spectroscopic data were in agreement with the values reported above.

The bulk product was analyzed by IR spectroscopy, which can be used to distinguish between cocrystals with H_2O and H_2O_2.

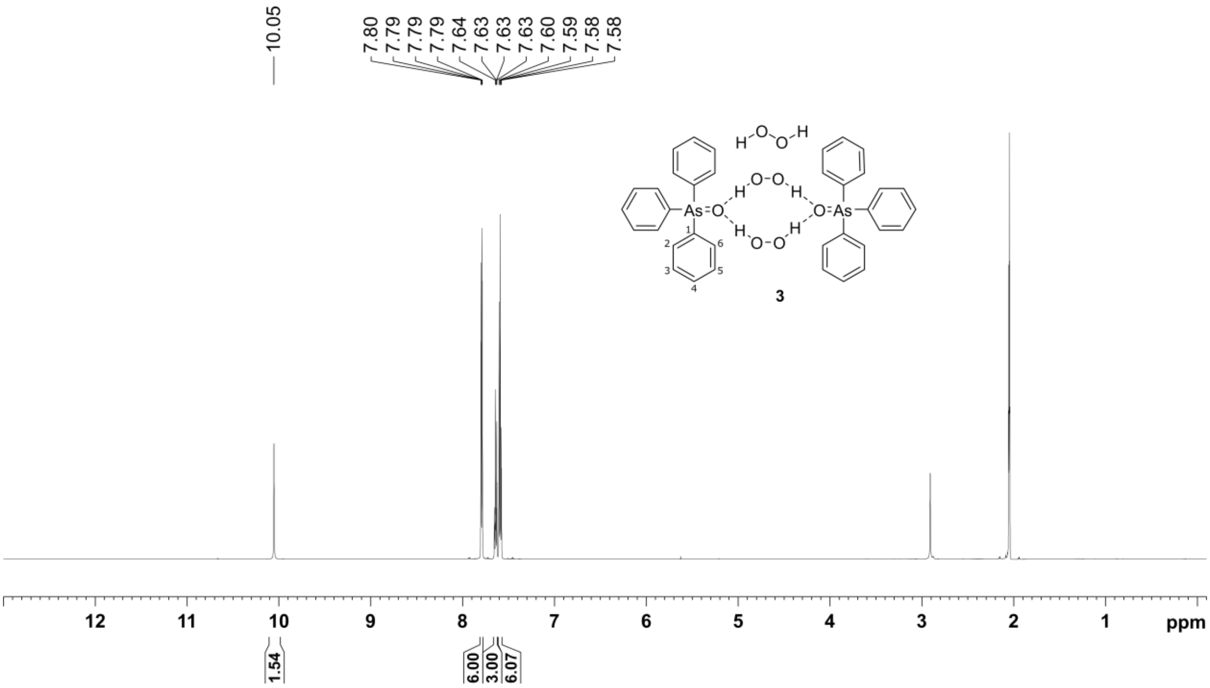

Figure S2. ^{1}H NMR (600 MHz, acetone-d_6) spectrum of **3**.

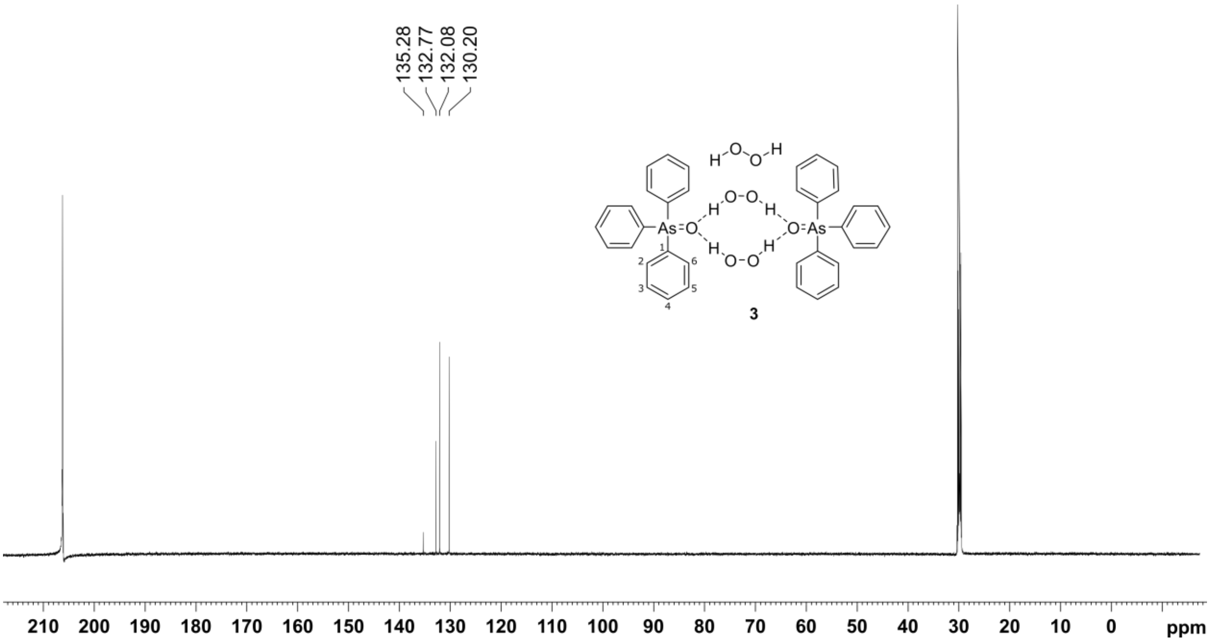

Figure S3. $^{13}C\{^{1}H\}$ NMR (150 MHz, acetone-d_6) spectrum of **3**.

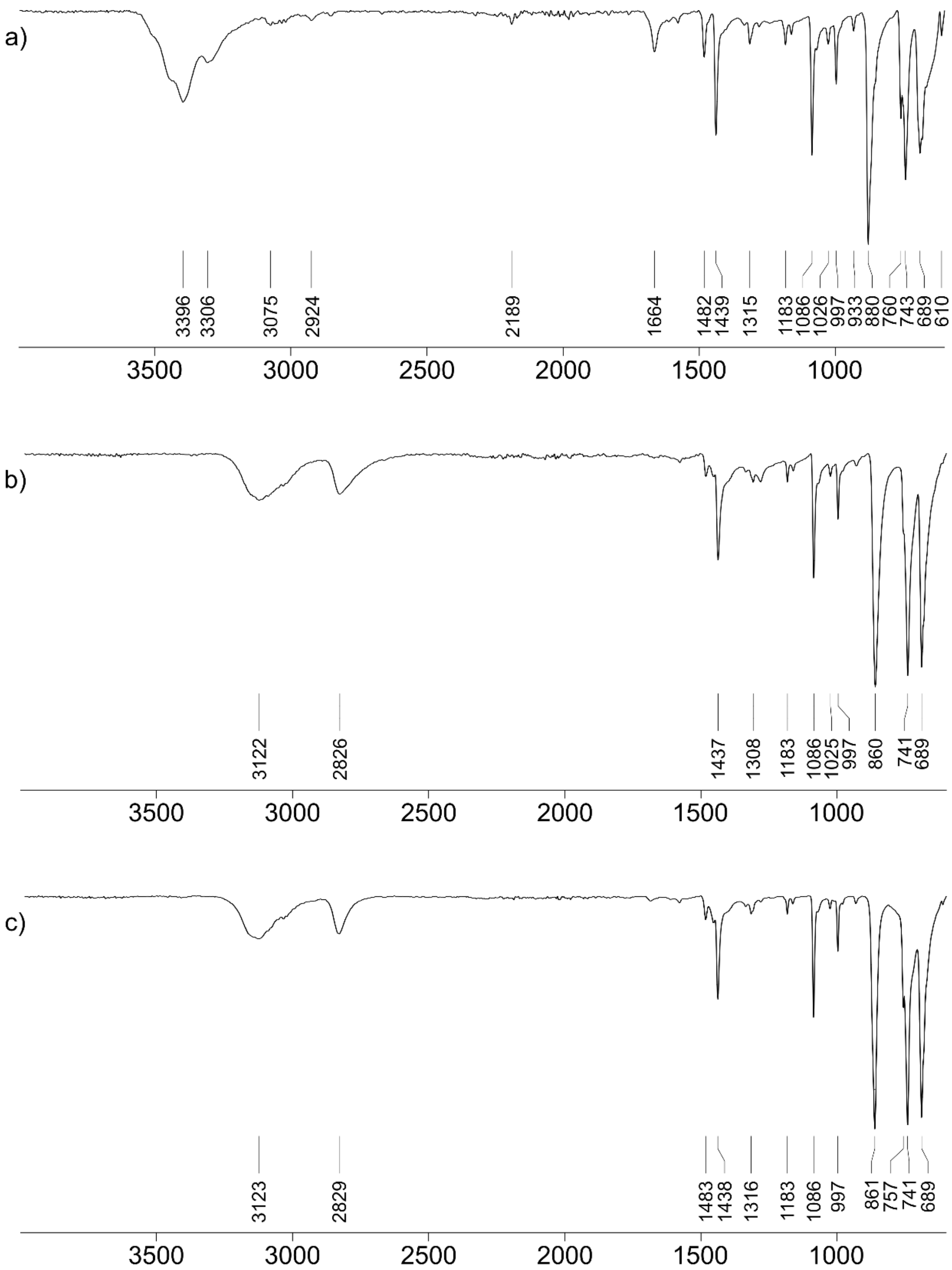

Figure S4. IR spectroscopic analysis of **$Ph_3AsO{\cdot}H_2O$** (a), **$[Ph_3AsO{\cdot}H_2O_2]_2{\cdot}H_2O_2$** prepared using neat H_2O_2 (b), and 30% aqueous solution of H_2O_2 (c).

General procedure for synthesis of *gem*-di(hydroperoxy)cycloalkanes **4**

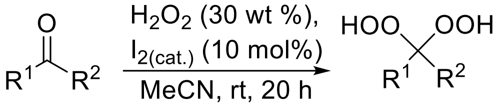

4

A slightly modified literature procedure[4] was used as follows. In a flame dried 25 mL flask, flushed with N_2, iodine (0.1 eq.) and H_2O_2 (4 eq., 30 wt %) were dissolved in dry acetonitrile (5 mL/1 mmol ketone). After addition of the ketone (1 eq.) the reaction mixture was stirred under nitrogen for 20 hours at room temperature. After that the solvent was removed under reduced pressure at room temperature. The crude product was redissolved in CH_2Cl_2 and dried over anhydrous Na_2SO_4. After evaporation of the solvent the desired product was obtained. The yields were determined by ^{1}H NMR with trimethoxybenzene as internal standard. The isolated *gem*-di(hydroperoxy)alkanes were used in the next step without further purification.

1,1-Dihydroperoxycyclopentane (**4a**)

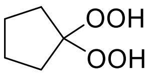

Following the general procedure employing cyclopentanone (177.1 µL, 2 mmol, 1 eq.), H_2O_2 (817 µL, 4 mmol, 4 eq., 30 wt %) and iodine (50.8 mg, 0.1 mmol, 0.1 eq.). 1,1-Dihydroperoxycyclopentane was obtained as a colorless oil (229 mg, 85%).

^{1}H (600 MHz, acetone-*d*$_6$): δ 10.44 (s, 2H), 1.89–1.85 (m, 4H), 1.68–1.64 (m, 4H).

^{13}C{^{1}H} (150 MHz, acetone-*d*$_6$): δ 121.4, 33.8, 25.2.

4-(*tert*-Butyl)-1,1-dihydroperoxycyclohexane (**4b**)

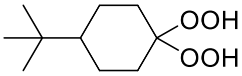

Following the general procedure employing 4-*tert*-butylcyclohexanone (308.5 mg, 2 mmol, 1 eq.), H_2O_2 (817 µL, 8 mmol, 4 eq., 30 wt %) and iodine (50.8 mg, 0.2 mmol, 0.1 eq.). The reaction mixture was stirred for 5 hours. 4-(*tert*-Butyl)-1,1-dihydroperoxycyclohexane was obtained as a white crystalline solid (310 mg, 76 %).

^{1}H (600 MHz, acetone-*d*$_6$): δ 10.14 (s, 1H), 10.06 (s, 1H), 2.27–2.22 (m, 2H), 1.69–1.63 (m, 2H), 1.35 (dt, J = 4.0, 13.3 Hz, 2H), 1.24 (dq, J = 3.3, 12.5, 2H), 1.08 (tt, J = 3.3, 11.9 Hz, 1H), 0.87 (s, 9H).

^{13}C{^{1}H} (150 MHz, acetone-*d*$_6$): δ 109.5, 48.3, 32.8, 30.7, 27.9, 24.2.

1,1-Dihydroperoxycycloheptane (**4c**)

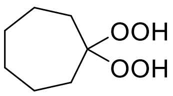

Following the general procedure employing cycloheptanone (236 µL, 2 mmol, 1 eq.), H_2O_2 (817 µL, 8 mmol, 4 eq., 30 wt %) and iodine (50.8 mg, 0.2 mmol, 0.1 eq.). 1,1-Dihydroperoxycycloheptane was obtained as a white solid (251 mg, 77 %).

^{1}H (600 MHz, acetone-*d*$_6$): δ 10.07 (s, 2H), 1.86 (m, 4H), 1.55 (m, 8H).

^{13}C{^{1}H} (150 MHz, acetone-*d*$_6$): δ 114.3, 33.4, 30.8, 23.5.

1,1-Dihydroperoxycyclododecane (**4d**)

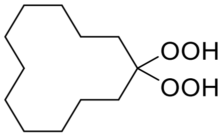

Following the general procedure employing cyclododecanone (364.6 mg, 2 mmol, 1 eq.), H_2O_2 (817 µL, 8 mmol, 4 eq., 30 wt %) and iodine (50.8 mg, 0.2 mmol, 0.1 eq.). 1,1-Dihydroperoxycyclododecane was obtained as a white solid (112 mg, 46 %).

^{1}H (600 MHz, acetone-*d*$_6$): δ 10.07 (s, 2H), 1.63–1.58 (m, 4H), 1.55–1.48 (m, 4H), 1.37 (s, 14H).

^{13}C{^{1}H} (150 MHz, acetone-*d*$_6$): δ 114.0, 26.92, 26.91, 26.7, 22.9, 22.6, 20.0.

2,2-Dihydroperoxyadamantane (**4e**)

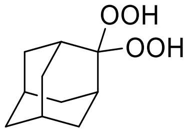

Following the general procedure employing 2-adamantanone (300.4 mg, 2 mmol, 1 eq.), H_2O_2 (817 µL, 8 mmol, 4 eq., 30 wt %) and iodine (50.8 mg, 0.2 mmol, 0.1 eq.), dissolved in 3 mL acetonitrile. The reaction mixture was stirred for 5 hours. 2,2-Dihydroperoxyadamantane was obtained as a white solid (160 mg, 80 %).

^{1}H (600 MHz, acetone-*d*$_6$): δ 9.97 (s, 2H), 2.30 (s, 2H), 1.99 (d, J = 12.5 Hz, 4H), 1.83–1.80 (m, 2H), 1.71–1.68 (m, 2H), 1.64 (d, J = 12.5 Hz, 4H)

^{13}C{^{1}H} (150 MHz, acetone-*d*$_6$): δ 111.4, 37.9, 34.4, 32.1, 28.1.

General procedure for the synthesis of Ph_3AsO·dhp cocrystals **5**

In a 50 mL round bottom flask equimolar amounts of Ph_3AsO (**2**) and dhp (**4**) were dissolved in 5 mL of CH_2Cl_2. Hexane (5 mL) was added and the reaction mixture was slowly concentrated under vacuum at room temperature. When a cloudy white solid began forming, the evaporation was stopped, and the flask was left for additional 15 minutes in the fumehood. After that colorless crystals of the desired products were collected.

Cocrystal $Ph_3AsO\cdot(HOO)_2(c\text{-}C_5H_8)$ (**5a**)

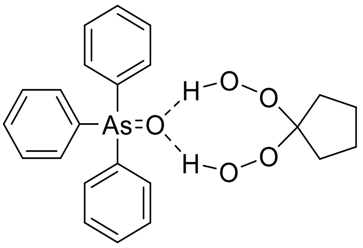

Following the general procedure for the synthesis of Ph_3AsO·dhp cocrystals employing Ph_3AsO (**2**, 119.1 mg, 0.37 mmol, 1 eq.) and 1,1-dihydroperoxycyclopentane (**4a**, 50 mg, 0.37 mmol, 1 eq.). Colorless crystals (168 mg, 88%).

IR (cm^{-1}): 3149, 2853, 1483, 1438, 1324, 1183, 1086, 1024, 997, 971, 867, 738, 689.

^{1}H (600 MHz, acetone-*d*$_6$): δ 11.92 (br, OOH), 7.83–7.79 (m, 6H, H-2, H-6), 7.72–7.68 (m, 3H, H-4), 7.67–7.62 (m, 6H, H-3, H-5), 1.87–1.83 (m, 4H, H-2’, H-5’), 1.67–1.63 (m, 4H, H-3’, H-4’).

^{13}C{^{1}H} (150 MHz, acetone-*d*$_6$): δ 134.2 (C-1), 133.1 (C-4), 132.1 (C-2, C-6), 130.4 (C-3, C-5), 121.0 (C-1’), 34.1 (C-2’, C-5’), 33.9 (C-2’, C-5’), 25.3 (C-3’, C-4’), 25.2 (C-3’, C-4’).

mp 111.6–118.1 °C

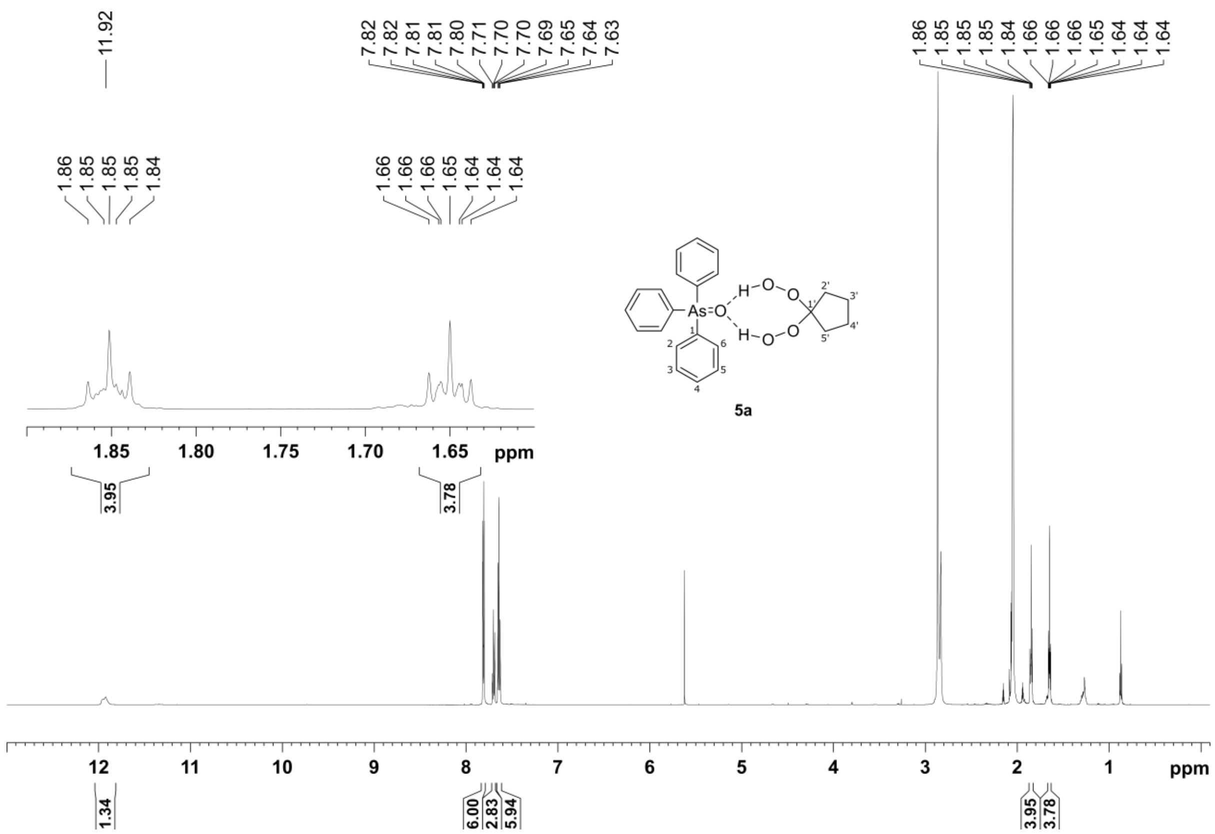

Figure S5. ^{1}H NMR (600 MHz, acetone-d_6) spectrum of **5a**.

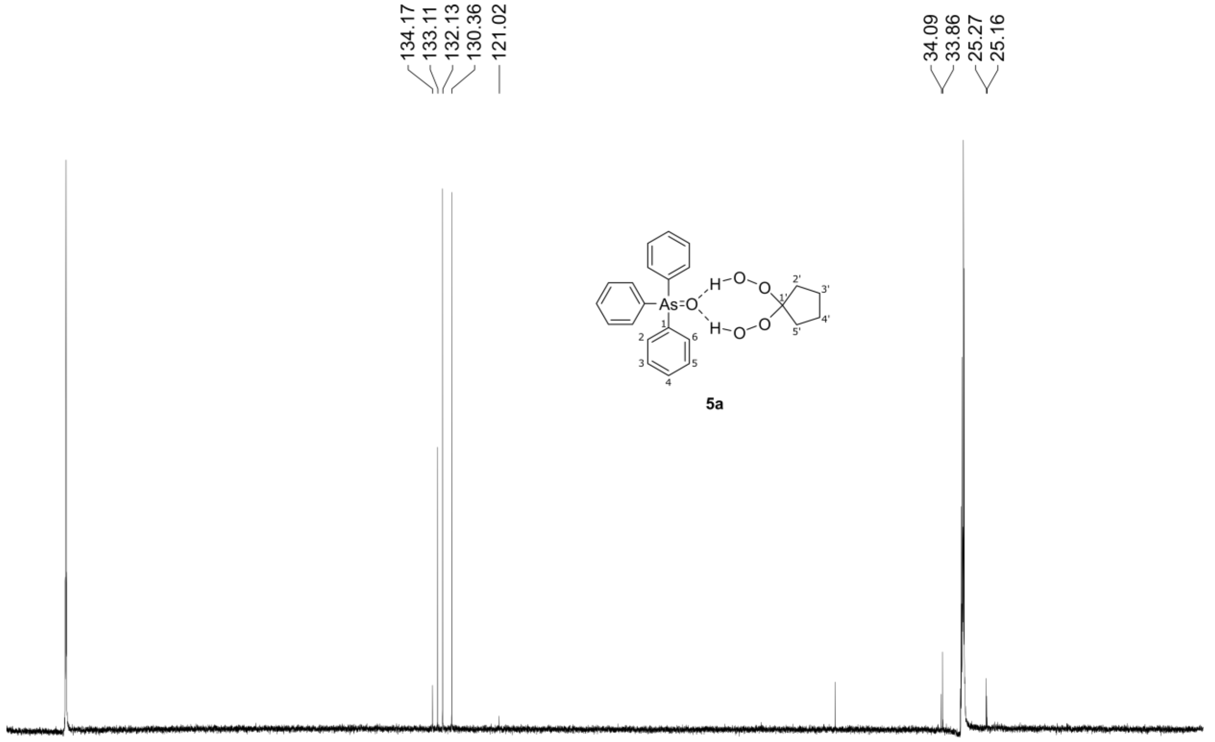

Figure S6. $^{13}C\{^{1}H\}$ NMR (150 MHz, acetone-d_6) spectrum of **5a**.

Cocrystal $Ph_3AsO\cdot(HOO)_2(4\text{-}^tBu\text{-}c\text{-}C_6H_9)$ (**5b**)

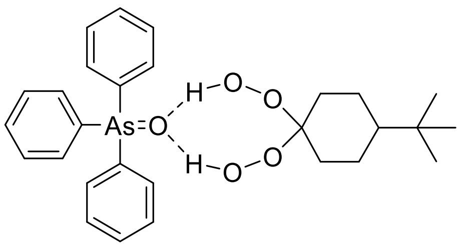

Following the general procedure for the synthesis of Ph_3AsO·dhp cocrystals employing Ph_3AsO (**2**, 39.9 mg, 0.124 mmol, 1 eq.) and 4-(*tert*-butyl)-1,1-dihydroperoxycyclohexane (**4b**, 25.3 mg, 0.124 mmol, 1 eq.). Colorless crystals (55.5 mg, 85%).

IR (cm^{-1}): 3159, 2956, 2857, 1437, 1087, 1058, 872, 741, 689.

^{1}H (600 MHz, acetone-*d*$_6$): δ 11.68 (br, 1H), 11.59 (br, 1H), 7.83–7.79 (m, 6H, H-2, H-6), 7.71–7.66 (m, 3H, H-4), 7.65–7.61 (m, 6H, H-3, H-5), 2.28–2.23 (m, 2H; H-2', H-6'), 1.69–1.62 (m, 2H, H-3', H-5'), 1.37–1.20 (m, 4H, H-2', H-3', H-5', H-6'), 1.11–1.04 (m, 1H, H-4'), 0.87 (s, 9H, H-8').

^{13}C{^{1}H} (150 MHz, acetone-*d*$_6$): δ 133.7 (C-1), 133.4 (C-4), 132.1 (C-2, C-6), 130.5 (C-3, C-5), 109.0 (C-1'), 48.5 (C-4'), 32.8 (C-7'), 30.9 (C-2', C-6'), 28.0 (C-8'), 24.2 (C-3', C-5').

mp 138.1–142.4 °C

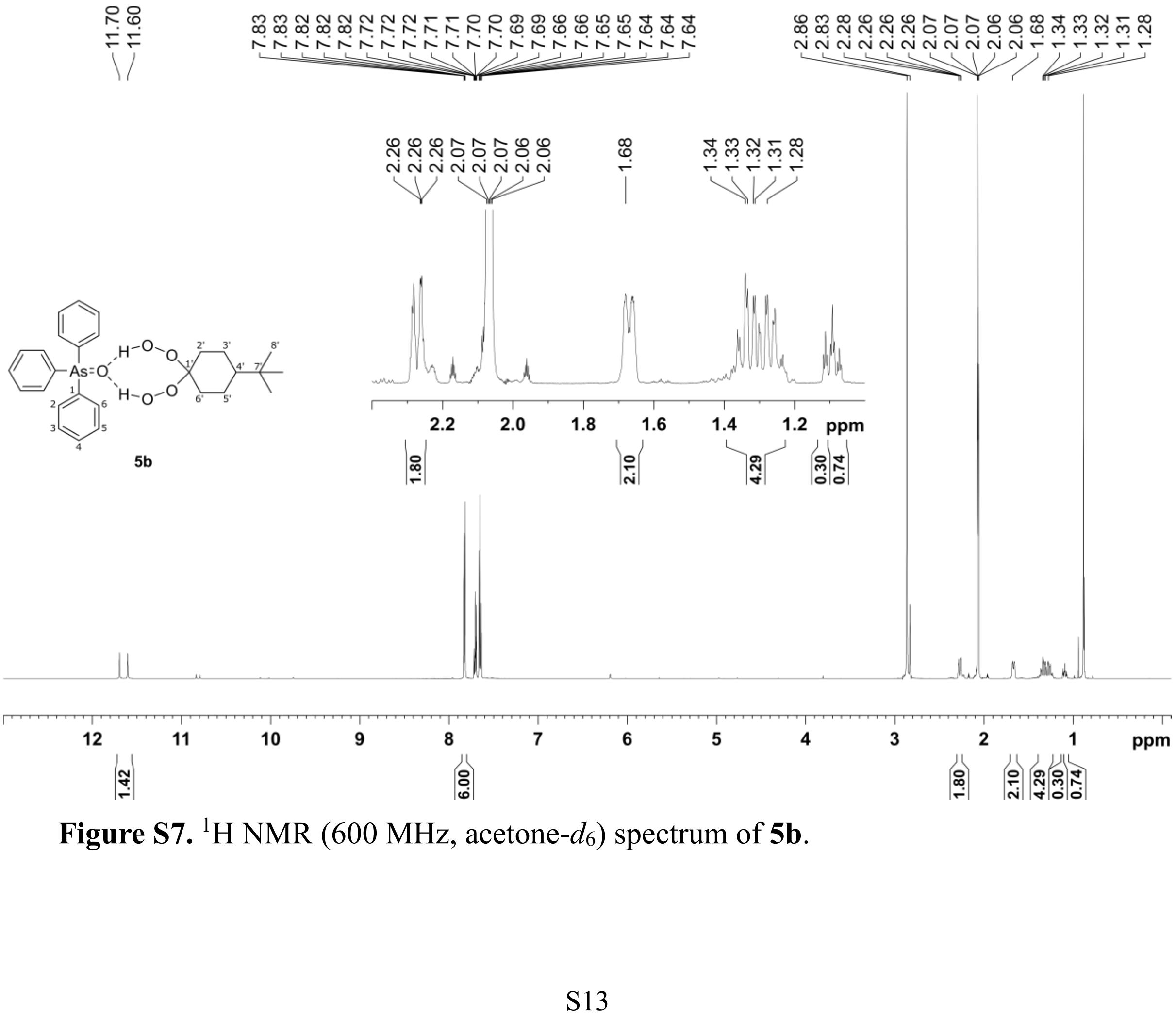

Figure S7. ^{1}H NMR (600 MHz, acetone-*d*$_6$) spectrum of **5b**.

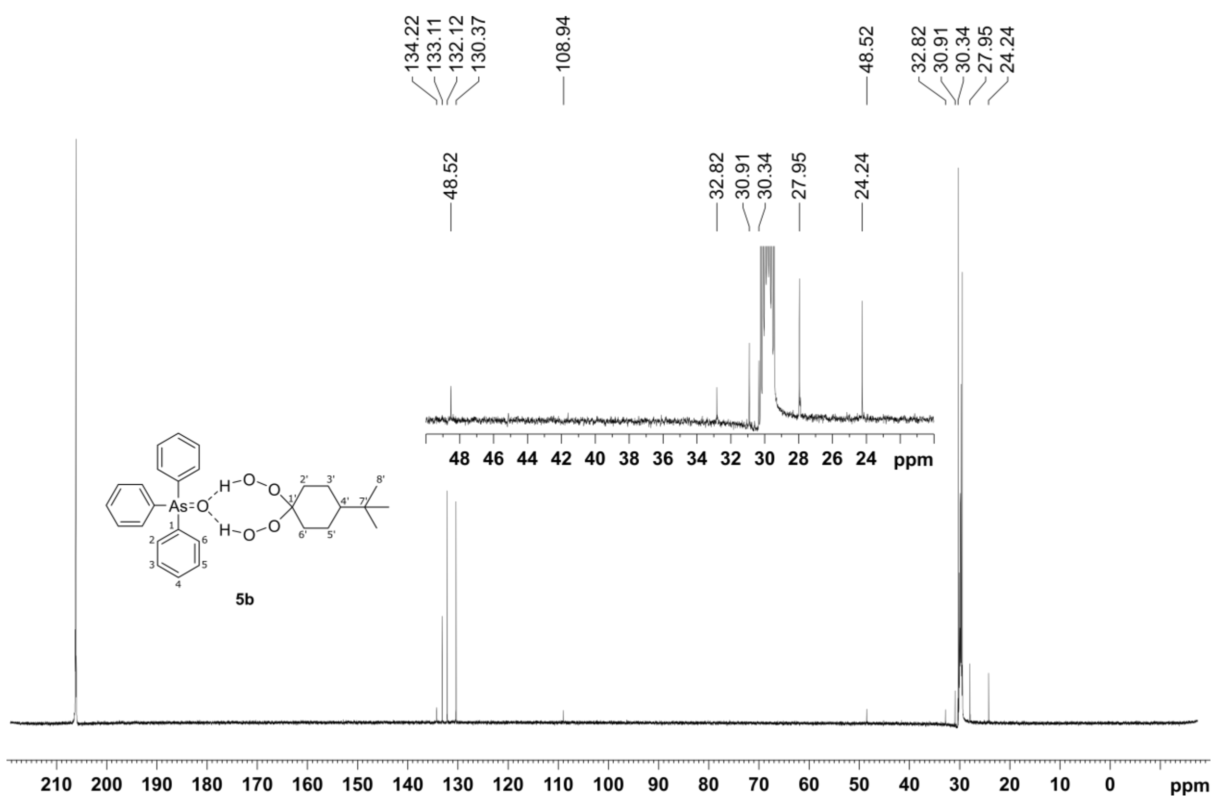

Figure S8. $^{13}C\{^{1}H\}$ NMR (150 MHz, acetone-d_6) spectrum of **5b**.

<u>Cocrystal $Ph_3AsO{\cdot}(HOO)_2(c\text{-}C_7H_{12})$ (**5c**)</u>

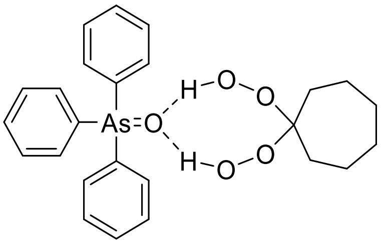

Following the general procedure for the synthesis of Ph_3AsO·dhp cocrystals employing Ph_3AsO (**2**, 45.7 mg, 0.142 mmol, 1 eq.) and 1,1-dihydroperoxycycloheptane (**4c**, 23 mg, 0.142 mmol, 1 eq.). Colorless crystals (51 mg, 74%).

IR (cm^{-1}): 3112, 2927, 2853, 1480, 1437, 1087, 1016, 868, 736, 689.

^{1}H (600 MHz, acetone-*d*$_6$): δ 11.66 (br, OOH), 7.83–7.79 (m, 6H, H-2, H-6), 7.71–7.66 (m, 3H, H-4), 7.65–7.61 (m, 6H, H-3, H-5), 1.91–1.82 (m, 4H, H-2', H-7'), 1.59–1.49 (m, 8H, H-3', H-4', H-5', H-6').

^{13}C{^{1}H} (150 MHz, acetone-*d*$_6$): δ 133.6 (C-1), 133.3 (C-4), 132.1 (C-2, C-6), 130.5 (C-3, C-5), 114.4 (C-1'), 33.2 (C-2', C-7'), 30.9 (C-3', C-6'), 23.6 (C-4', C-5').

mp 109.9–114.4 °C

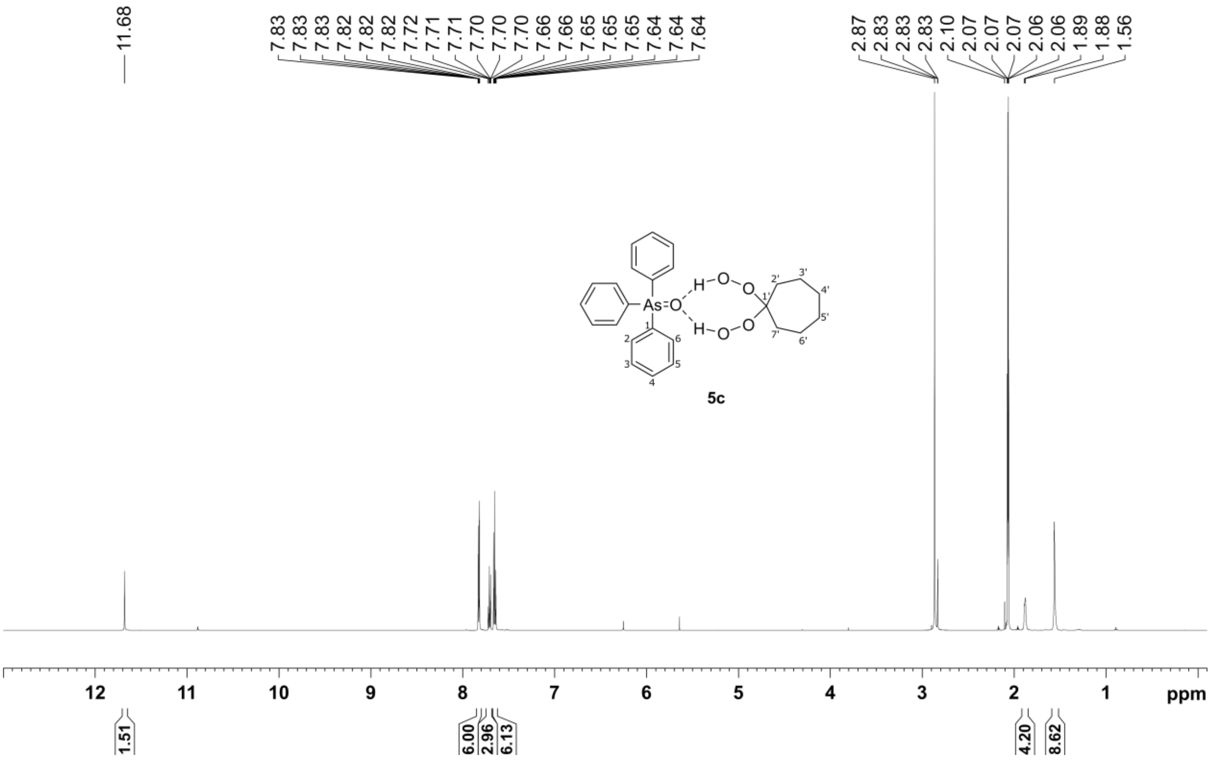

Figure S9. ^{1}H NMR (600 MHz, acetone-d_6) spectrum of **5c**.

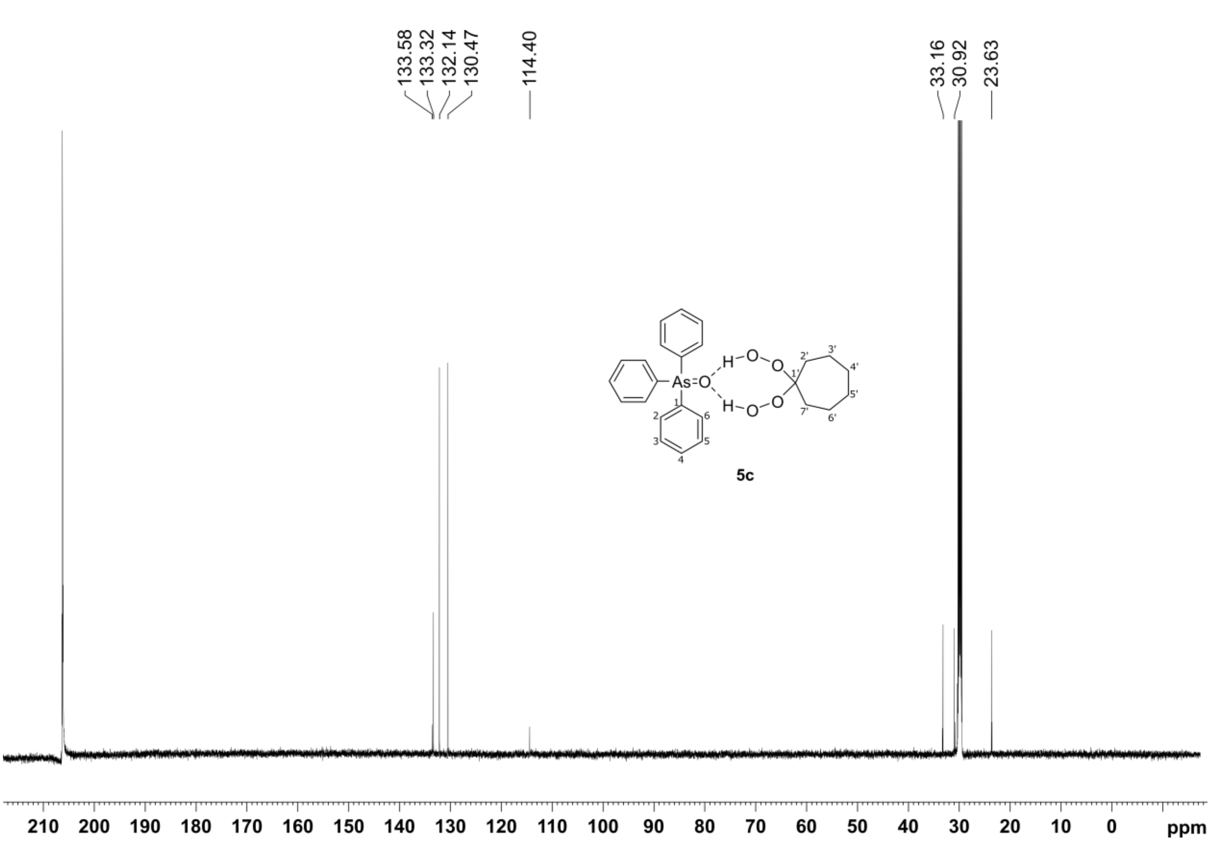

Figure S10. ^{13}C{^{1}H} NMR (150 MHz, acetone-d_6) spectrum of **5c**.

Cocrystal $Ph_3AsO·(HOO)_2(c\text{-}C_{12}H_{22})$ (**5d**)

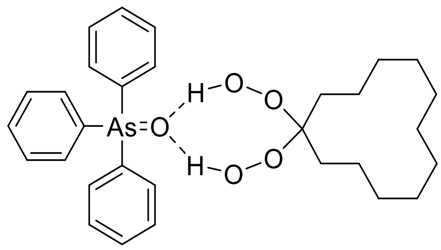

Following the general procedure for the synthesis of Ph_3AsO·dhp cocrystals employing Ph_3AsO **2** (17.4 mg, 0.054 mmol, 1 eq.) and 1,1-dihydroperoxycyclododecane (**4d**,12.5 mg, 0.054 mmol, 1 eq.). Colorless crystals (21.5 mg, 72%).

IR (cm^{-1}): 3122, 2842, 1467, 1437, 1156, 1087, 1055, 998, 867, 738, 688.

^{1}H (600 MHz, acetone-*d*$_6$): δ 11.63 (br, OO*H*), 7.82–7.79 (m, 6H, H-2, H-6), 7.71–7.66 (m, 3H, H-4), 7.65–7.61 (m, 6H, H-3, H-5), 1.64–1.58 (m, 4H, H-2', H-3', H-11', H-12'), 1.54–1.46 (m, 4H, H-2', H-3', H-11', H-12'), 1.37 (s, 14H, H-4'–H-10').

^{13}C{^{1}H} (150 MHz, acetone-*d*$_6$): δ 133.7 (C-1), 133.3 (C-4), 132.1 (C-2, C-6), 130.5 (C-3, C-5), 113.6 (C-1'), 26.9 (C-2', C-12'/C-4'–C-10'), 26.7 (C-2', C-12'/C-4'–C-10'), 22.9 (C-4'–C-10'), 22.6 (C-4'– C-10'), 20.0 (C-3', C-11').

mp 138.4–143.7 °C

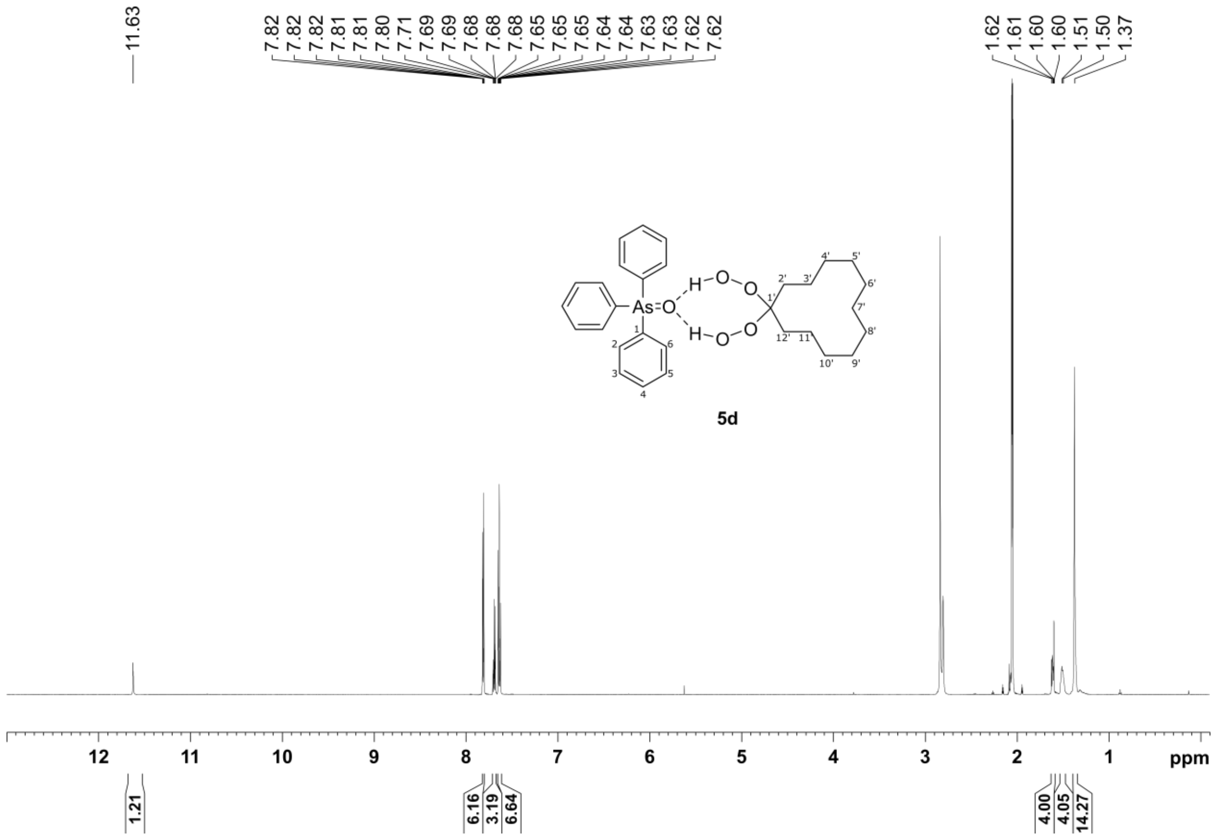

Figure S11. ^{1}H NMR (600 MHz, acetone-*d*$_6$) spectrum of **5d**.

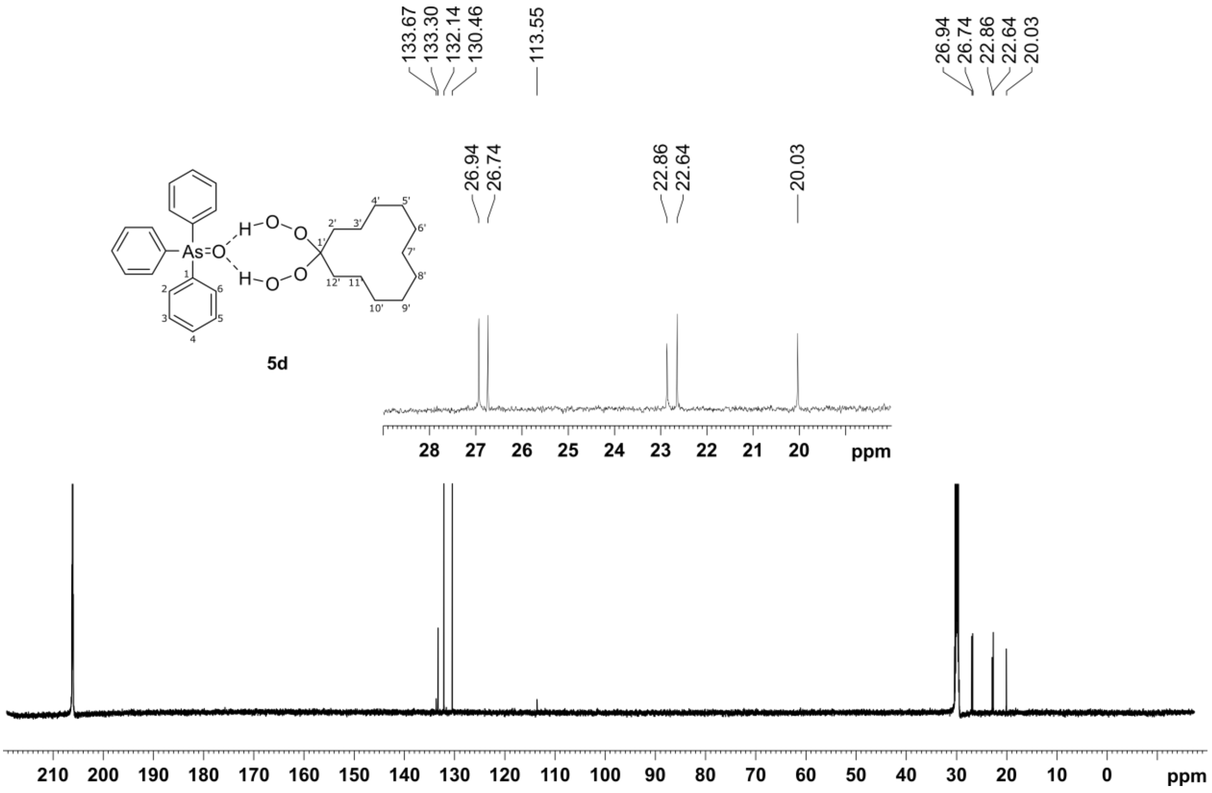

Figure S12. $^{13}C\{^{1}H\}$ NMR (150 MHz, acetone-d_6) spectrum of **5d**.

<u>Cocrystal $Ph_3AsO\cdot(HOO)_2(adm)$ (**5e**)</u>

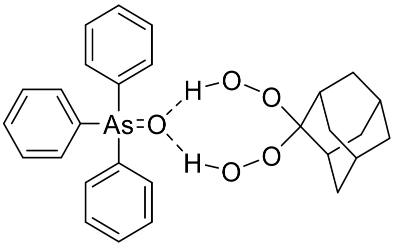

Following the general procedure for the synthesis of Ph_3AsO·dhp cocrystals employing Ph_3AsO (**2**, 41.9 mg, 0.13 mmol, 1 eq.) and 2,2-dihydroperoxyadamantane (**4e**, 26.1 mg, 0.13 mmol, 1 eq.). Colorless crystals (34.1 mg, 51%).

IR (cm^{-1}): 3148, 2910, 2849, 1716, 1457, 1087, 995, 922, 864, 739, 689.

^{1}H (600 MHz, acetone-*d*$_6$): δ 11.92 (br, OOH), 7.83–7.78 (m, 6H, H-2, H-6), 7.70–7.65 (m, 3H, H-4), 7.64–7.59 (m, 6H, H-3, H-5), 2.29 (s, 2H, H-2', H-8'), 2.01 (d, 4H, J = 12.3 Hz, H-3', H-7'), 1.82–1.78 (m, 2H, H-4'. H-6'), 1.71–1.68 (m, 2H, H-10'), 1.62 (d, 4H, J = 12.3 Hz, H-5', H-9').

$^{13}C\{^{1}H\}$ (150 MHz, acetone-*d*$_6$): δ 133.4 (C-1), 133.2 (C-4), 132.2 (C-2, C-6), 130.5 (C-3, C-5), 110.8 (C-1'), 38.1 (C-10'), 34.5 (C-3', C-5', C-7, C-9'), 32.3 (C-2', C-8'), 28.5 (C-4', C-6').

mp 136.9–139.3 °C

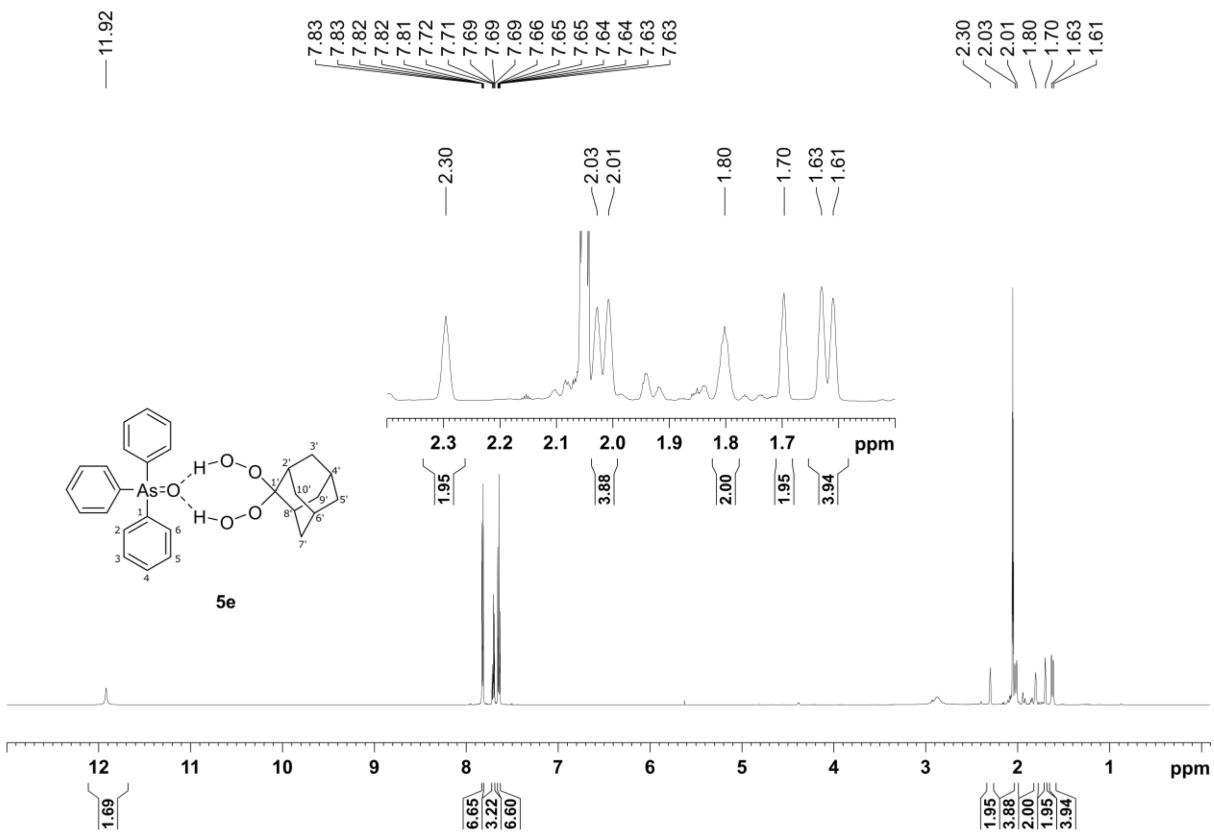

Figure S13. ^{1}H NMR (600 MHz, acetone-d_6) spectrum of **5e**.

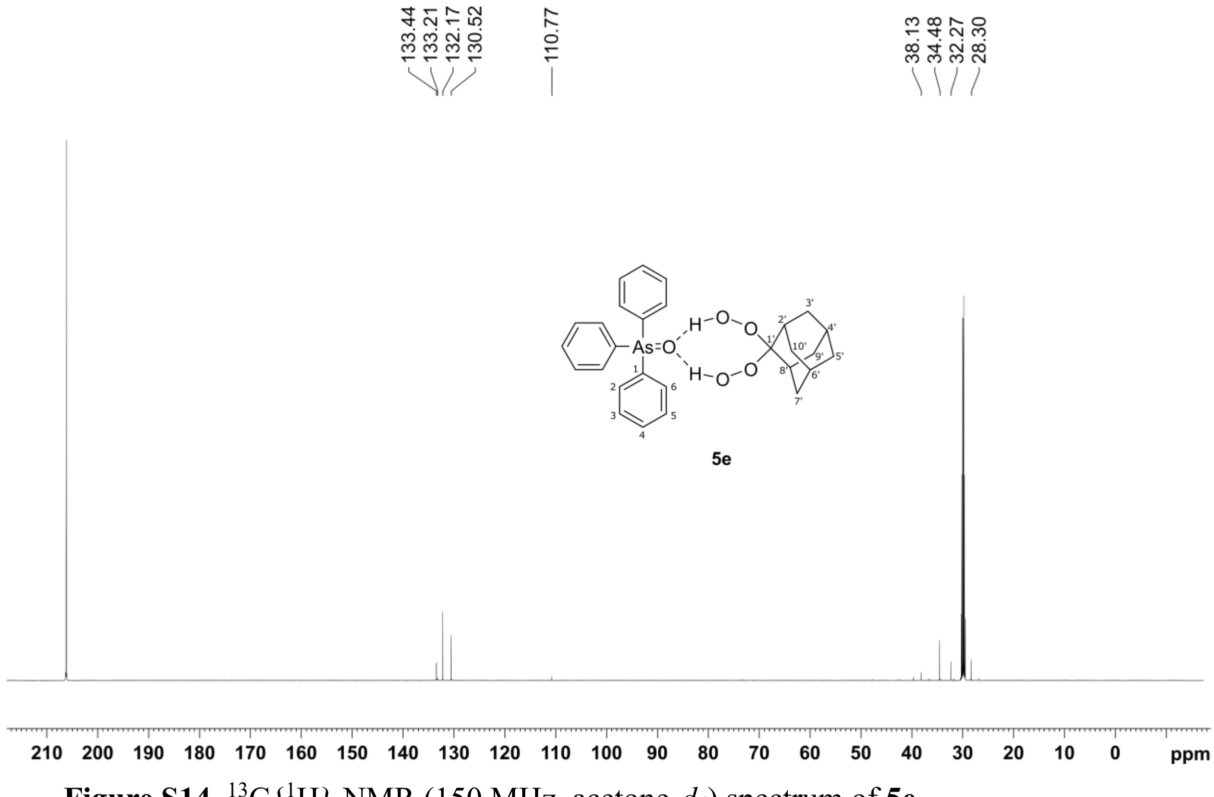

Figure S14. $^{13}C\{^{1}H\}$ NMR (150 MHz, acetone-d_6) spectrum of **5e**.

Miscellaneous experiments

Oxidation of triphenylphosphine and monitoring the oxidative ability of **3**

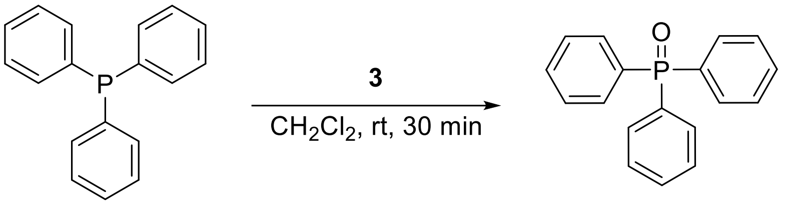

In a 10 mL round bottom flask triphenylphosphine (20.0 mg, 0.076 mmol, 1.5 eq.) and **3** (19.0 mg, 0.051 mmol, 1 eq., refrigerated at 5 °C) were weighed and dissolved in 1 mL of CH_2Cl_2. The reaction mixture was stirred at room temperature for 30 minutes. The solvent was removed under reduced pressure. About 5 mg of this crude product was dissolved in 0.6 mL acetone-d_6 and used for NMR analysis. The reaction yield and oxidative ability of **3** were calculated from the ^{1}H integral ratios of excess triphenylphosphine and its oxide.

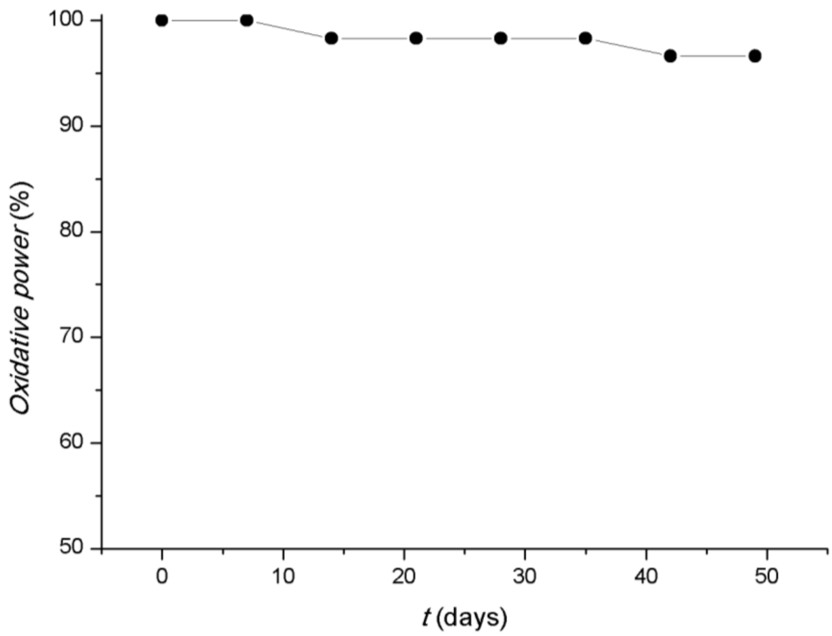

Figure S15. Stability of **3** over longer time period.

Synthesis of $[Ph_3PO·H_2O_2]_2·H_2O_2$

$[Ph_3PO·H_2O_2]_2·H_2O_2$ was prepared according to a known procedure.[5] In a 25 mL round bottom flask Ph_3PO (304.5 mg, 1.09 mmol, 1 eq.) and H_2O_2 (557 µL, 5.45 mmol, 5 eq., 30 wt %) were dissolved in 5 mL of acetone. After slow evaporation of the solvent colorless crystals were formed, which were collected on a glass frit and thoroughly washed with hexane (250 mg, 70%). The spectroscopic data agree well with the literature.[5]

^{1}H (500 MHz, $CDCl_3$): δ 11.26 (br, 2H), 8.43 (br, 1H), 7.69–7.61 (m, 6H), 7.60–7.54 (m, 3H), 7.51–7.44 (m, 6H).

^{31}P (202 MHz, $CDCl_3$): δ 30.21.

Competition tests

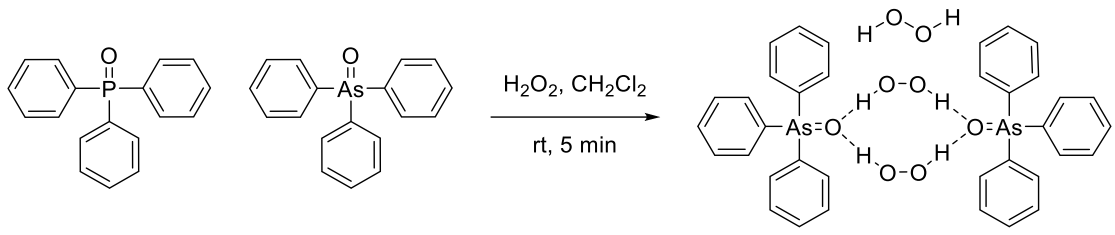

Scheme S1. Competition test between Ph_3PO and Ph_3AsO with H_2O_2.

In a 10 mL round bottom flask Ph_3PO (58.2 mg, 0.209 mmol, 1 eq.) and Ph_3AsO (67.2 mg, 0.209 mmol, 1 eq.) were weighed and dissolved in 5 mL of CH_2Cl_2. H_2O_2 (832 μL, 0.209 mmol, 1 eq., 30 wt %) was added. The reaction mixture was stirred at room temperature for 5 minutes and dried over anhydrous Na_2SO_4. The solvent was removed under reduced pressure. About 5 mg of this crude product was dissolved in 0.6 mL $CDCl_3$ and used for ^1H and ^{31}P NMR analysis.

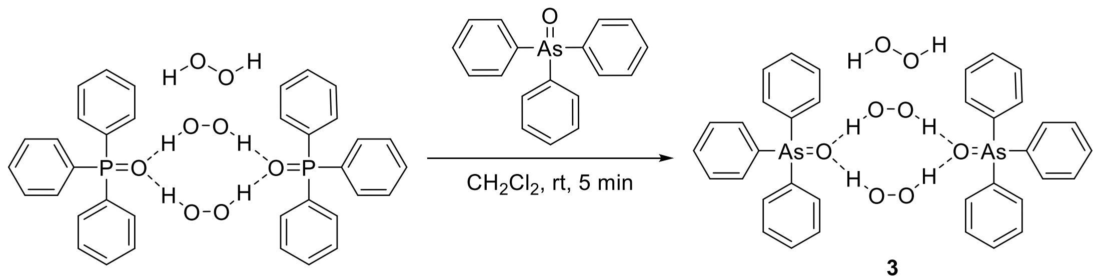

3

Scheme S2. Competition test between $[Ph_3PO{\cdot}H_2O_2]_2{\cdot}H_2O_2$ and Ph_3AsO.

To a 5 mL solution of $[Ph_3PO{\cdot}H_2O_2]_2{\cdot}H_2O_2$ (51.9 mg, 0.079 mmol, 1 eq.) in CH_2Cl_2 Ph_3AsO was added (25.4 mg, 0.079 mmol, 1 eq.). The reaction mixture was stirred for 5 minutes, after which the solvent was removed under reduced pressure. About 5 mg of this crude product was dissolved in 0.6 mL $CDCl_3$ and used for ^1H and ^{31}P NMR analysis.

Solubility of selected adducts

The solubility of adducts **3** and **5d** was determined according to the literature procedure.[6] About 15 mg of the adduct was weighed in a tared vial. The solvent was added dropwise, while shaking the vial in between additions. After all solids were dissolved, the vial was weighed, and the added volume was calculated. The solubility was determined as the average of three measurements.

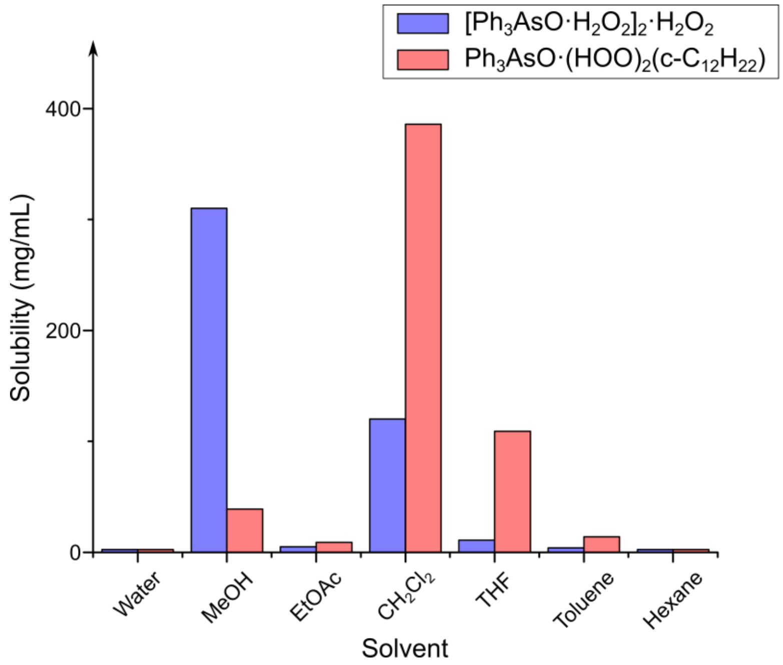

solvent	3	5d
water	<1	<1
MeOH	310±4	39±5
EtOAc	5±0.3	9±0.5
CH_2Cl_2	120±7	386±9
THF	11±1	109±7
Toluene	3±0.3	14±0.4
Hexane	<1	<1

Figure S16. Solubility of **3** and **5d** in several solvents at room temperature.

CRYSTALLOGRAPHIC PART

Crystal structures were measured on a Rigaku OD XtaLAB Synergy-S diffractometer equipped with Cu and Ag PhotonJet micro-focus sealed X-ray tubes and an Eiger2 R CdTe 1M hybrid pixel detector. In a typical measurement the datasets were collected with Cu Kα radiation at 100 K. Crystals of **5c** and **5d** undergo a destructive phase transition at low temperature and were therefore measured at 200 K. *CrysAlis^{Pro}* software[7] was used for data collection and reduction. Crystal structures were solved by *olex2.solve* and refined by *ShelXL* program[8] within the *Olex2* (v. 1.5) software.[9] *Diamond* program was used for visualization of crystallographic results.[10]

Summary of the crystal data and structure refinements

Table S1. Summary of the crystal data and structure refinements.

Compound	AsPh3O_1	**3**	**5a**
Formula	$C_{18}H_{15}A O$	$C_{18}H_{15}AsO \cdot 1.5(H_2O_2)$	$C_{18}H_{15}AsO \cdot C_5H_{10}O_4$
F_w	322.22	373.24	456.35
T [K]	100.00(1)	100.0(1)	100.0(1)
Crystal system	monoclinic	monoclinic	triclinic
Space group	$P2_1/n$	$P2_1/n$	$P{-}1$
a [Å]	6.26691(3)	9.72060(7)	9.43630(19)
b [Å]	27.75385(11)	16.81364(9)	9.7839(2)
c [Å]	8.50410(4)	10.96472(7)	12.3892(3)
α [°]	90	90	106.770(2)
β [°]	104.2014(5)	111.8330(8)	103.1680(18)
γ [°]	90	90	102.8829(18)
V [Å^3]	1433.922(12)	1663.52(2)	1013.96(4)
Z	4	4	2
ρ_{calc} [g cm^{-3}]	1.493	1.490	1.495
Crystal size [mm]	0.278 × 0.242 × 0.193	0.362 × 0.234 × 0.036	0.235 × 0.134 × 0.038
Radiation type	Cu Kα	Cu Kα	Cu Kα
λ [Å]	1.54184	1.54184	1.54184
μ [mm^{-1}]	3.130	2.918	2.543
F(000)	656	764	472
θ_{max} [°]	76.036	76.063	76.210
Index ranges	$-7 \le h \le 7$<br>$-34 \le k \le 34$<br>$-10 \le l \le 10$	$-12 \le h \le 10$<br>$-21 \le k \le 21$<br>$-13 \le l \le 13$	$-11 \le h \le 11$<br>$-12 \le k \le 12$<br>$-15 \le l \le 15$
Reflections collected	48813	61278	25090
Independent reflections	2988	3466	4110
Reflections with [$I > 2\sigma(I)$]	2987	3379	3754
R_{int}	0.0305	0.0423	0.0470
R_{sigma}	0.0093	0.0115	0.0244
Data/restraints/parameters	2988/10/263	3466/0/280	4110/0/271
S	1.124	1.084	1.085
R_1, wR_2 [$I > 2\sigma(I)$]	0.0225, 0.0595	0.0287, 0.0746	0.0462, 0.1229
R_1, wR_2 [all data]	0.0225, 0.0595	0.0292, 0.0749	0.0510, 0.1256
$\Delta\rho_{min}$, $\Delta\rho_{max}$ [eÅ^{-3}]	−0.468, 0.368	−0.580, 0.377	−0.782, 1.488
CCDC Deposition number[a]	2389149	2389150	2389148

[a] The supplementary crystallographic data for this paper can be obtained free of charge from The Cambridge Crystallographic Data Centre (CCDC) via www.ccdc.cam.ac.uk/structures.

Table S1. Summary of the crystal data and structure refinements. *(continued)*

Compound	**5b**	**5c**	**5d**	**5e**
Formula	$C_{18}H_{15}AsO{\cdot}C_{10}H_{20}O_4$	$C_{18}H_{15}AsO{\cdot}C_7H_{14}O_4$	$C_{18}H_{15}AsO{\cdot}C_{12}H_{24}O_4$	$C_{18}H_{15}AsO{\cdot}C_{10}H_{16}O_4$
F_w	526.48	484.40	554.53	522.45
T [K]	100.0(1)	200.0(1)	200.0(1)	100.00(1)
Crystal system	monoclinic	monoclinic	monoclinic	monoclinic
Space group	$P2_1/c$	$P2_1/c$	$P2_1/n$	$P2_1/n$
a [Å]	18.12858(11)	9.91983(6)	8.98385(10)	9.44919(13)
b [Å]	11.78225(7)	10.86421(7)	29.7970(3)	16.4260(3)
c [Å]	12.53933(8)	22.13734(14)	10.69261(9)	32.4169(5)
α [°]	90	90	90	90
β [°]	97.6566(6)	96.8967(6)	98.2370(9)	96.1481(13)
γ [°]	90	90	90	90
V [Å^3]	2654.46(3)	2368.50(3)	2832.79(5)	5002.58(13)
Z	4	4	4	8
ρ_{calc} [g cm^{-3}]	1.317	1.358	1.300	1.387
Crystal size [mm]	0.256 × 0.24 × 0.041	0.255 × 0.109 × 0.025	0.321 × 0.28 × 0.054	0.315 × 0.141 × 0.02
Radiation type	Cu Kα	Cu Kα	Cu Kα	Cu Kα
λ [Å]	1.54184	1.54184	1.54184	1.54184
μ [mm^{-1}]	2.012	2.208	1.911	2.135
F(000)	1104	1008	1168	2176
θ_{max} [°]	76.119	76.211	76.307	68.249
Index ranges	$-22 \leq h \leq 22$<br>$-14 \leq k \leq 14$<br>$-15 \leq l \leq 15$	$-12 \leq h \leq 12$<br>$-13 \leq k \leq 12$<br>$-27 \leq l \leq 27$	$-9 \leq h \leq 10$<br>$-37 \leq k \leq 37$<br>$-13 \leq l \leq 13$	$-11 \leq h \leq 11$<br>$-18 \leq k \leq 19$<br>$-39 \leq l \leq 39$
Reflections collected	104050	85124	93015	82652
Independent reflections	5536	4951	5861	9173
Reflections with [$I > 2\sigma(I)$]	5306	4535	5421	7971
R_{int}	0.0482	0.0465	0.0518	0.0673
R_{sigma}	0.0134	0.0157	0.0169	0.0275
Data/restraints/parameters	5536/0/447	4951/2/288	5861/2/334	9173/24/731
S	1.060	1.084	1.058	1.037
R_1, wR_2 [$I > 2\sigma(I)$]	0.0288, 0.0777	0.0321, 0.0884	0.0346, 0.0895	0.0390, 0.1009
R_1, wR_2 [all data]	0.0297, 0.0783	0.0344, 0.0901	0.0373, 0.0912	0.0460, 0.1053
$\Delta\rho_{min}$, $\Delta\rho_{max}$ [eÅ^{-3}]	−0.564, 0.537	−0.449, 0.634	−0.494, 0.338	−0.578, 0.845
CCDC Deposition number[a]	2389154	2389151	2389153	2389152

[a] The supplementary crystallographic data for this paper can be obtained free of charge from The Cambridge Crystallographic Data Centre (CCDC) via www.ccdc.cam.ac.uk/structures.

Ph$_3$AsO (**2**)

The crystal structure of **2** was previously determined (CSD refcode: BOTMEC[11]) at room temperature and has now been redetermined at low temperature. The space group at 100 K is $P2_1/n$, while the previously reported structure is isostructural but described in the $P2_1/c$ setting. The low-temperature crystal structure exhibits a rotational disorder of one Ph ring about its C^{ipso}–C^{para} axis with partial occupancies of 0.547(16) and 0.453(16). As−O distance was determined as 1.6527(11) Å. The hydrogen atoms were freely refined (positions and isotropic displacement parameters) with the exception of the hydrogens in the disordered part, where they were placed in calculated positions using the standard riding model (AFIX 43).

The phenyl groups are virtually parallel to the As=O bond. This is due to the presence of weak C−H···O hydrogen bonds (Table S4, page S57).

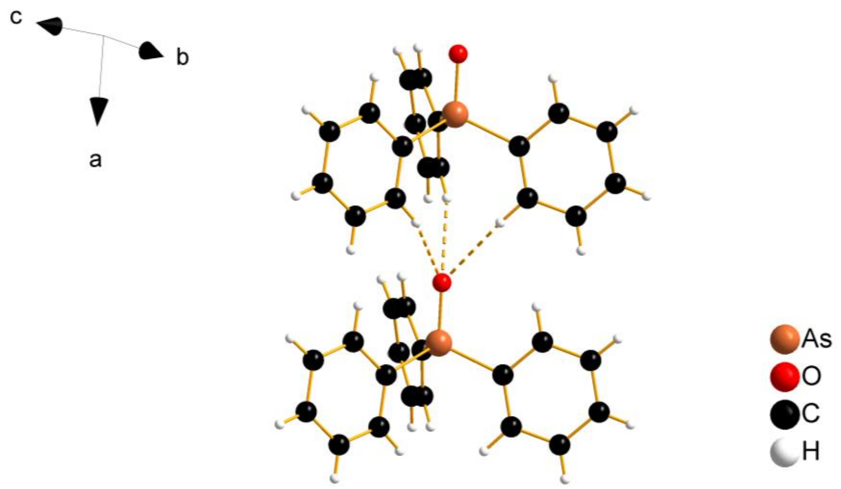

Figure S17. A stacking motif of Ph$_3$AsO molecules in the crystal structure of **2**.

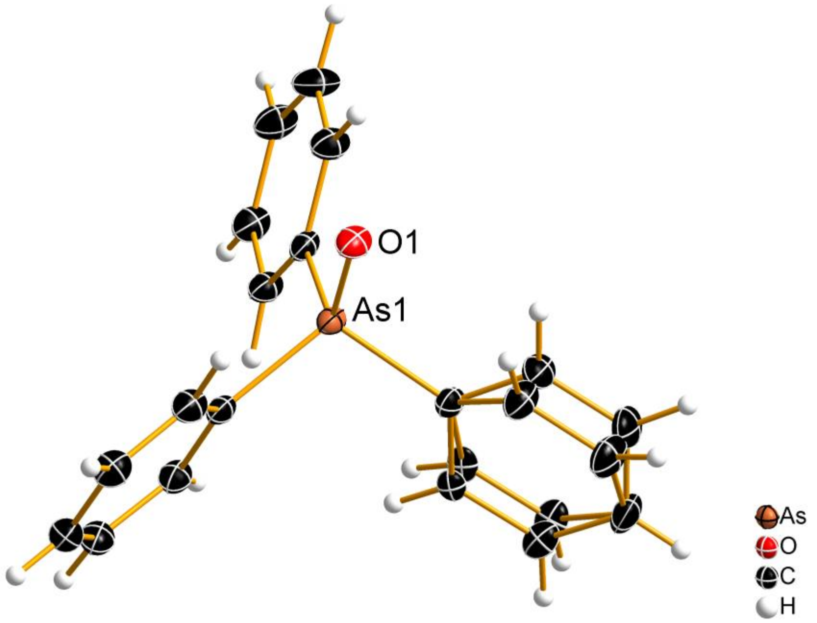

Figure S18. The asymmetric unit and selected atom labels of the Ph_3AsO (**2**) crystal structure with displayed disordered atoms. Displacement ellipsoids are depicted at the 50% probability level and hydrogen atoms are shown as small spheres of arbitrary radius.

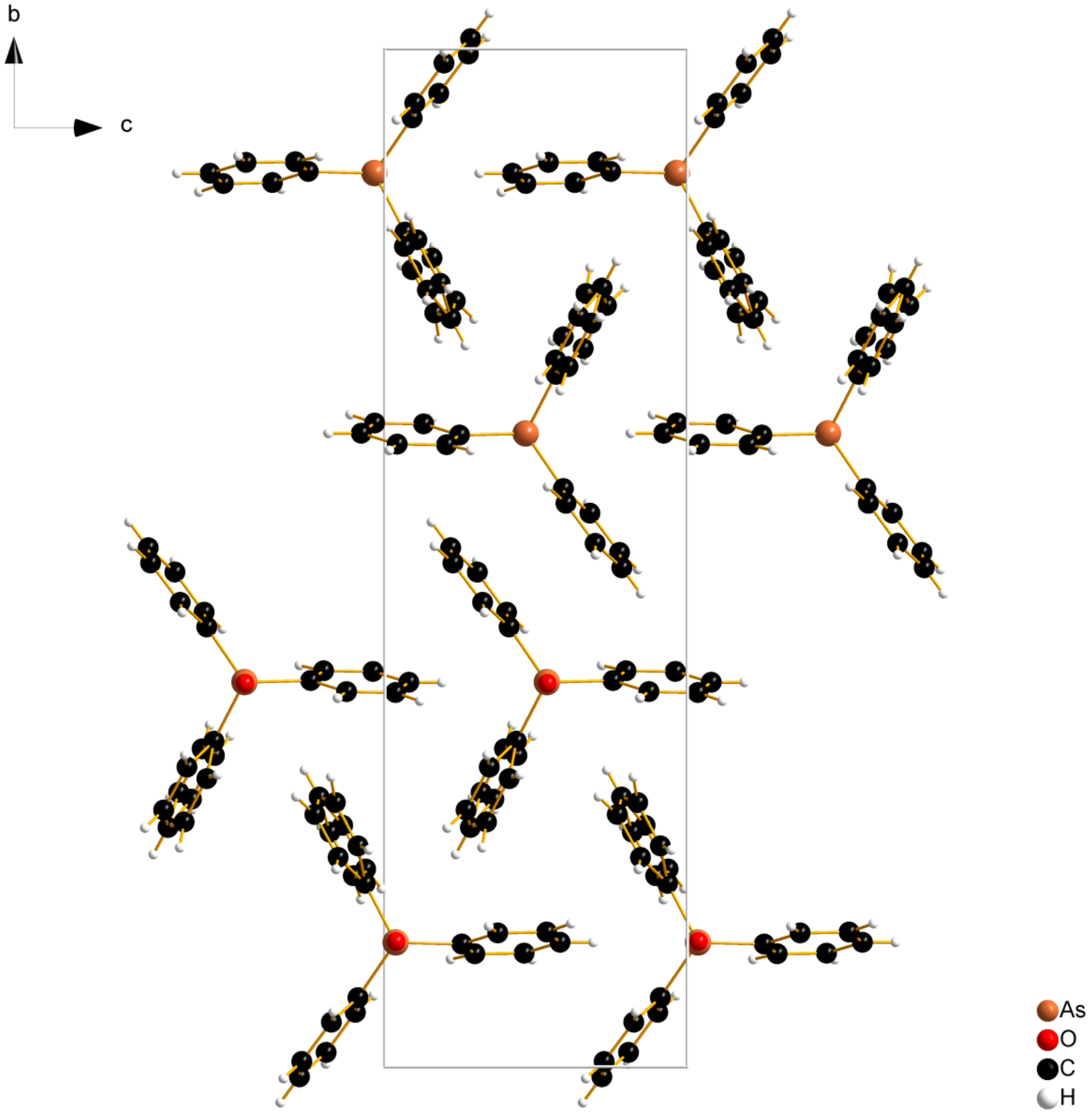

Figure S19. The crystal packing and the unit cell of the Ph_3AsO (**2**) crystal structure viewed along the *a*-crystallographic axis.

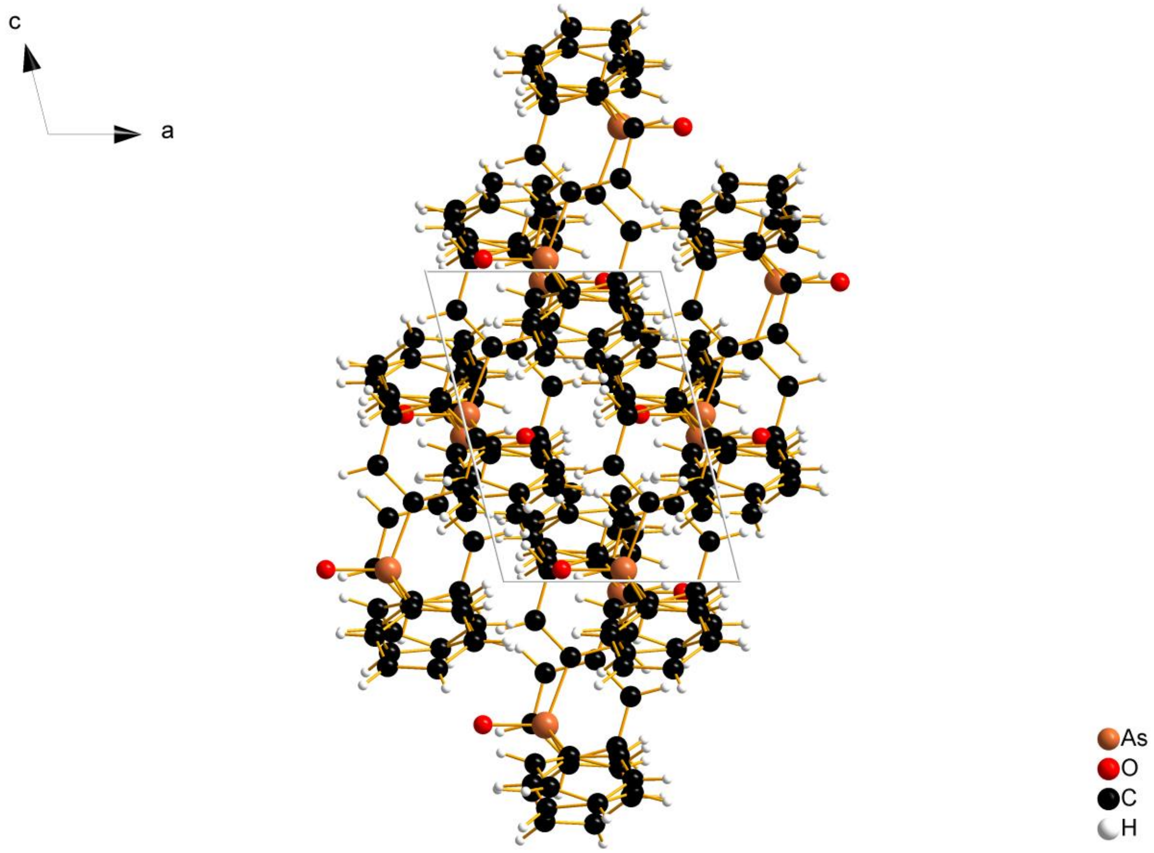

Figure S20. The crystal packing and the unit cell of the Ph_3AsO (**2**) crystal structure viewed along the *b*-crystallographic axis.

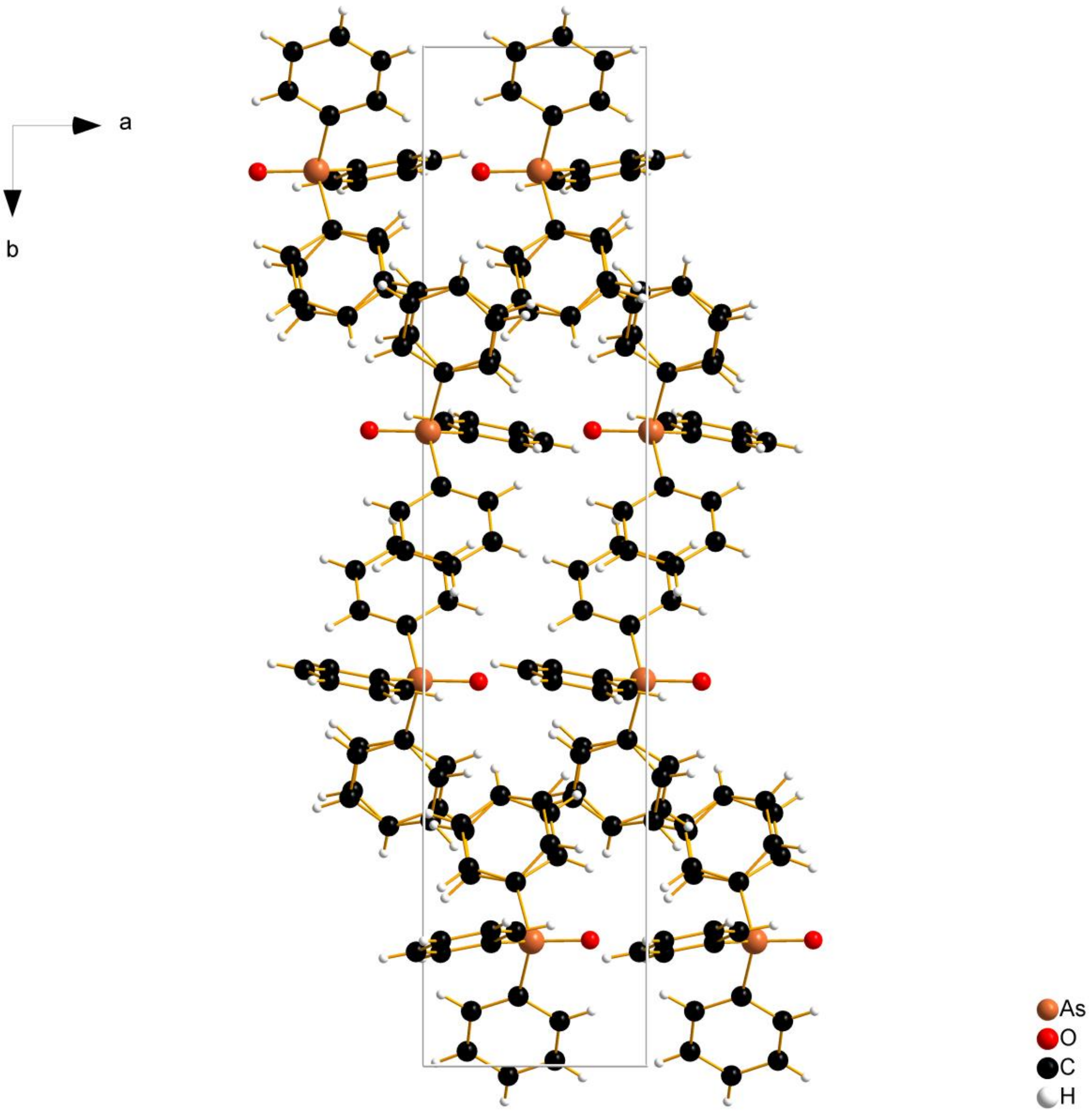

Figure S21. The crystal packing and the unit cell of the Ph_3AsO (**2**) crystal structure viewed along the *c*-crystallographic axis.

[$Ph_3AsO{\cdot}H_2O_2]_2{\cdot}H_2O_2$ (**3**)

The crystal structure was measured at 100 K and it was determined that the compound crystallizes in the $P2_1/n$ space group. The high quality of the data allowed for the free refinement of positions and isotropic displacement parameters of all hydrogen atoms, the detailed hydrogen-bonds parameters are provided below (Table S2, page S56).

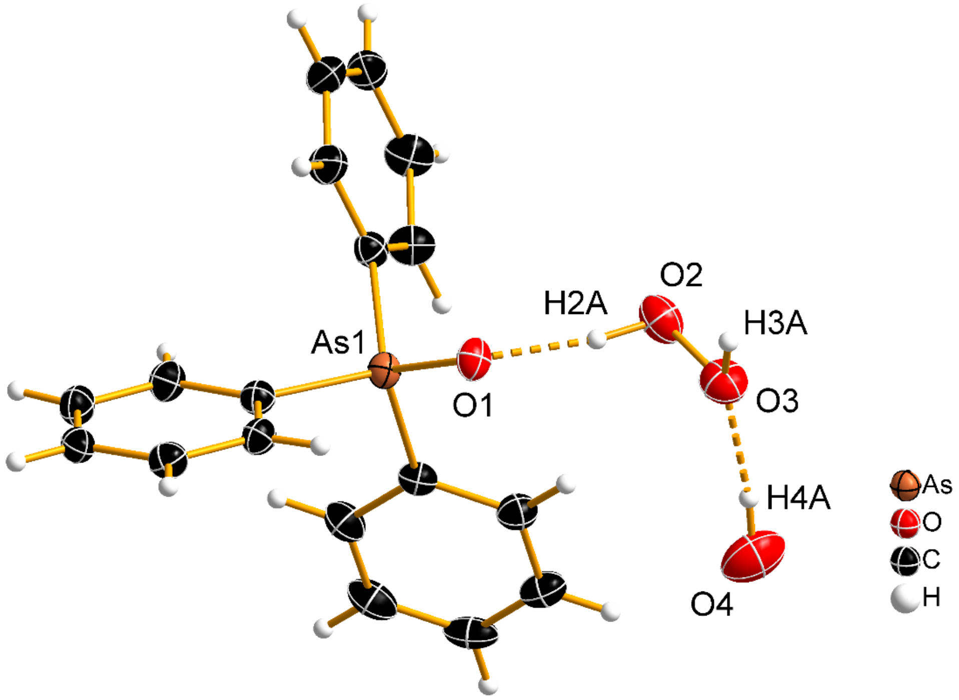

Figure S22. The asymmetric unit and selected atom labels of the H_2O_2 cocrystal with Ph_3AsO (**3**) crystal structure. Displacement ellipsoids are depicted at the 50% probability level and hydrogen atoms are shown as small spheres of arbitrary radius.

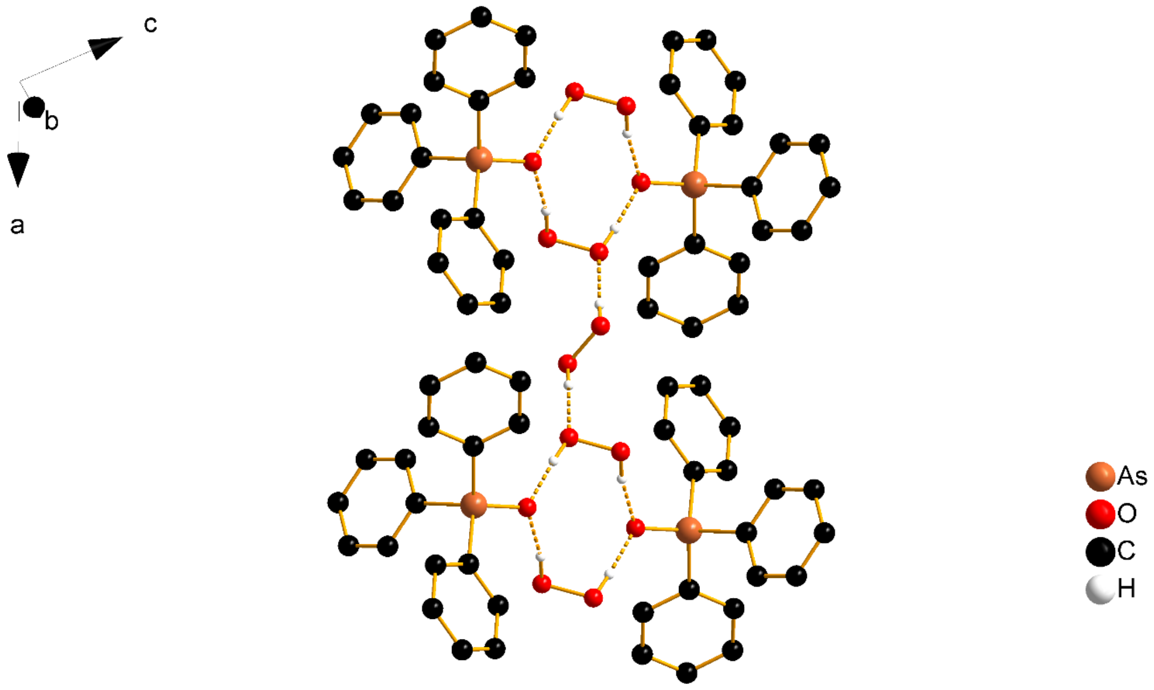

Figure S23. The periodic motif of the H_2O_2 cocrystal with Ph_3AsO (**3**) crystal structure. Hydrogen atoms are omitted for clarity, except for hydrogen atoms involved in hydrogen-bonding.

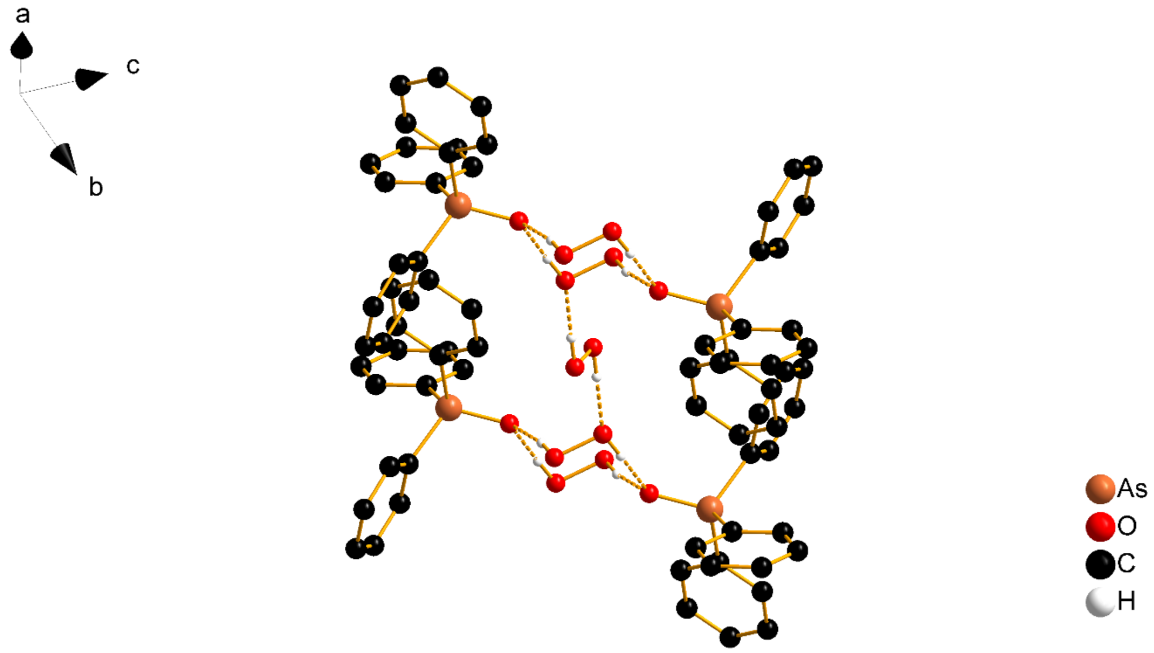

Figure S24. The periodic motif of the H_2O_2 cocrystal with Ph_3AsO (**3**) in a different orientation, showcasing the chair conformation formed by oxygens. Hydrogen atoms are omitted for clarity, except for hydrogen atoms involved in hydrogen-bonding.

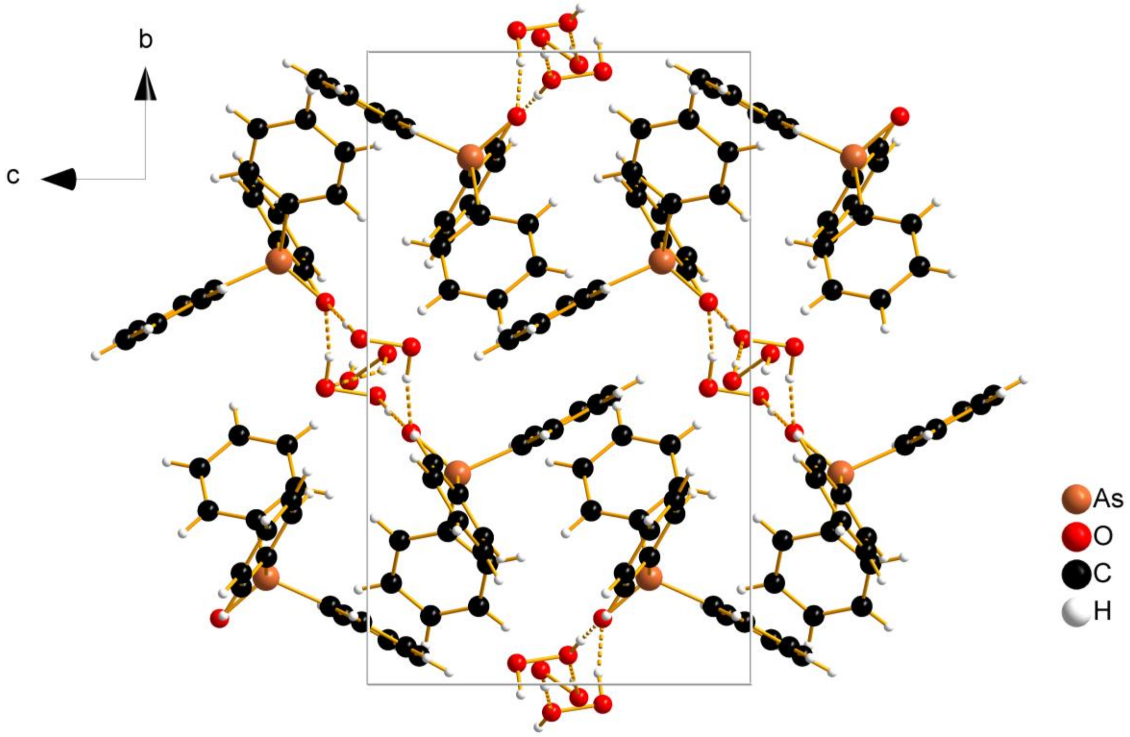

Figure S25. The crystal packing and the unit cell of the H_2O_2 cocrystal with Ph_3AsO (**3**) crystal structure viewed along the *a*-crystallographic axis.

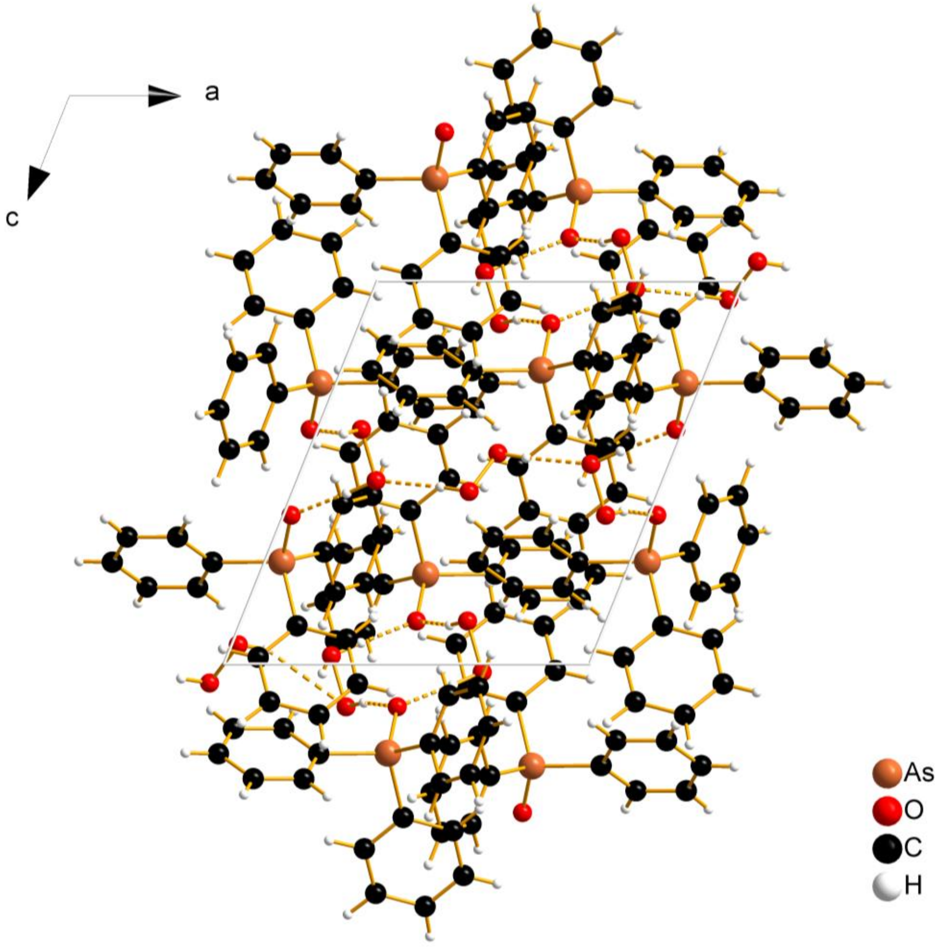

Figure S26. The crystal packing and the unit cell of the H_2O_2 cocrystal with Ph_3AsO **(3)** crystal structure viewed along the *b*-crystallographic axis.

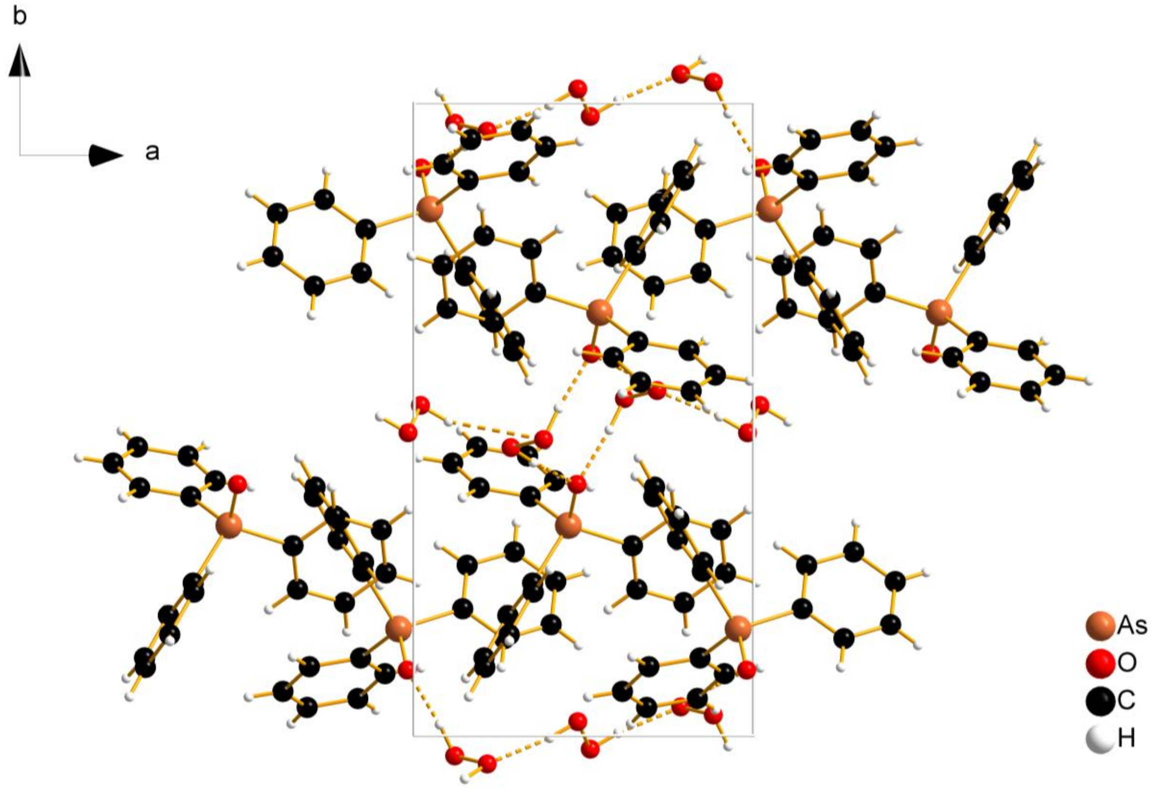

Figure S27. The crystal packing and the unit cell of the H_2O_2 cocrystal with Ph_3AsO (**3)** crystal structure viewed along the *c*-crystallographic axis.

Ph$_3$AsO·(HOO)$_2$(c-C$_5$H$_8$) (**5a**)

The crystal structure was measured at 100 K and it was determined that the compound crystallizes in the *P*–1 space group. The hydrogen atoms were placed at the calculated positions with the exception of hydrogen atoms involved in the hydrogen bonds, which were freely refined (positions and isotropic displacement parameters). The detailed hydrogen bonds parameters are provided below (Table S2, page S56).

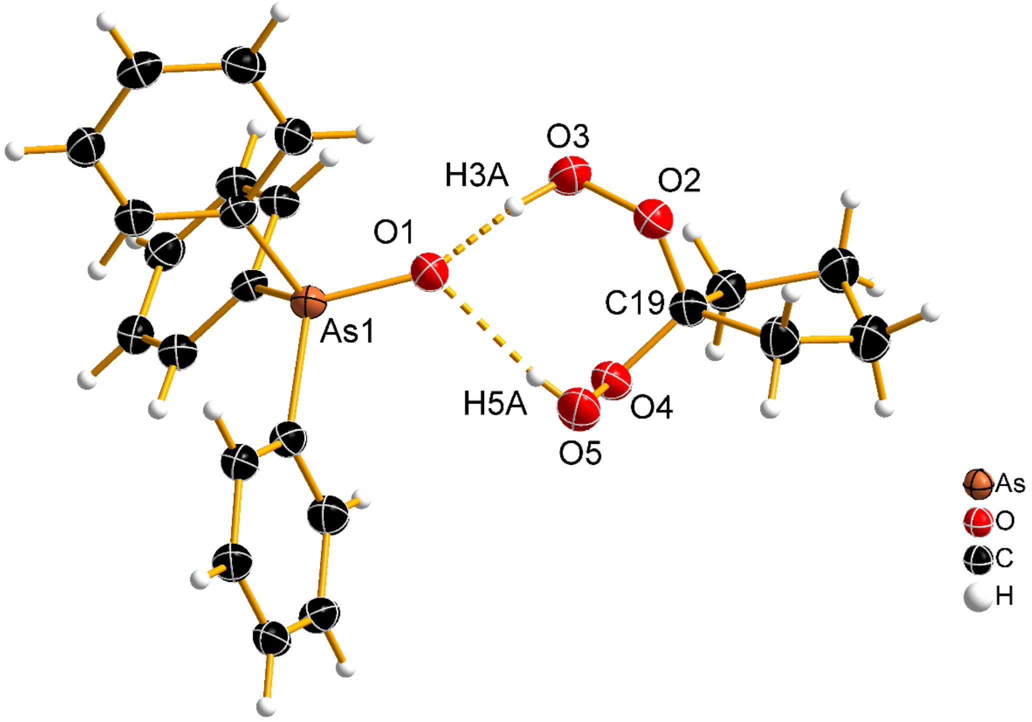

Figure S28. The asymmetric unit and selected atom labels of the **5a** crystal structure. Displacement ellipsoids are depicted at the 50% probability level and hydrogen atoms are shown as small spheres of arbitrary radius.

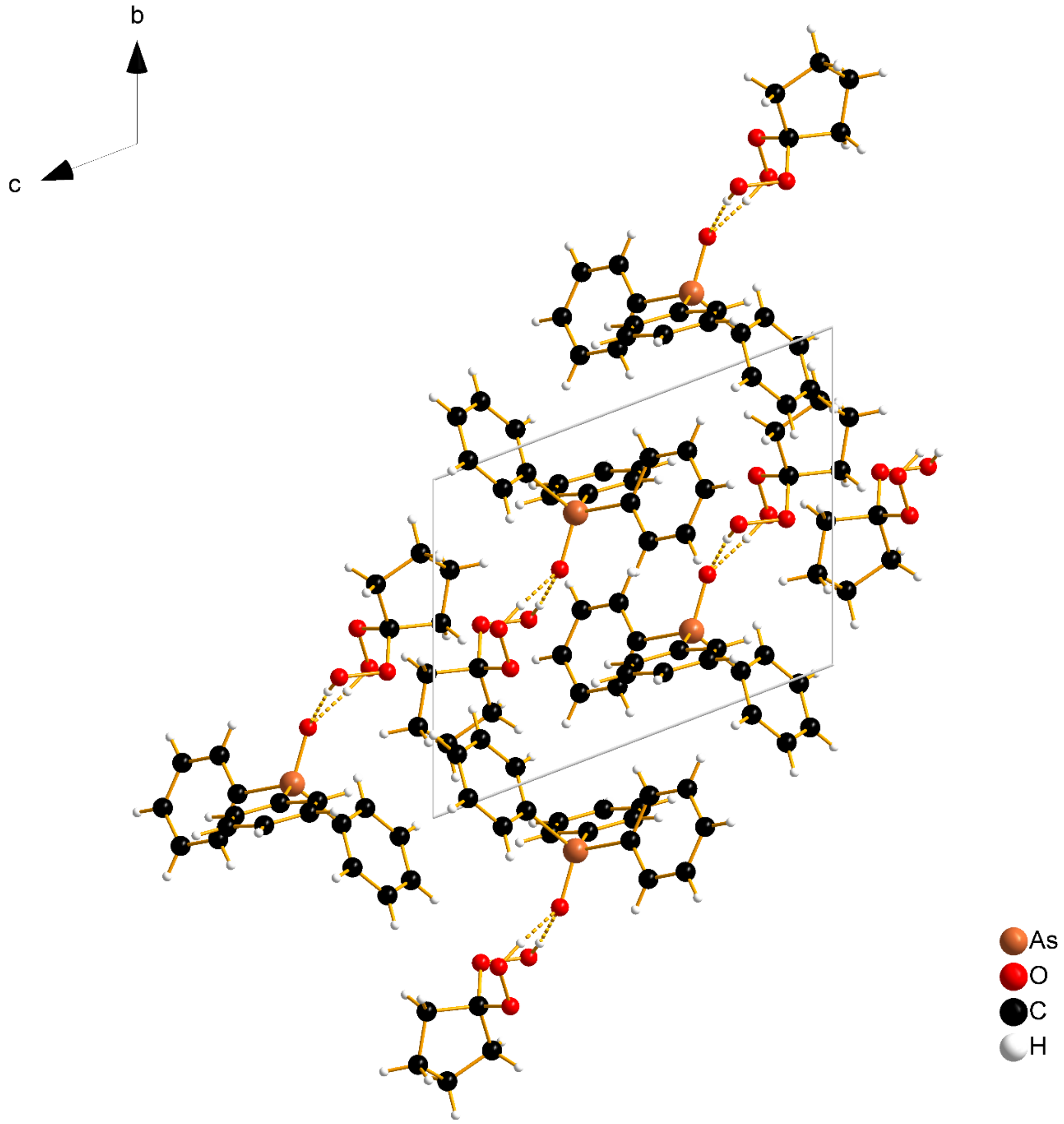

Figure S29. The crystal packing and the unit cell of the **5a** crystal structure viewed along the *a*-crystallographic axis.

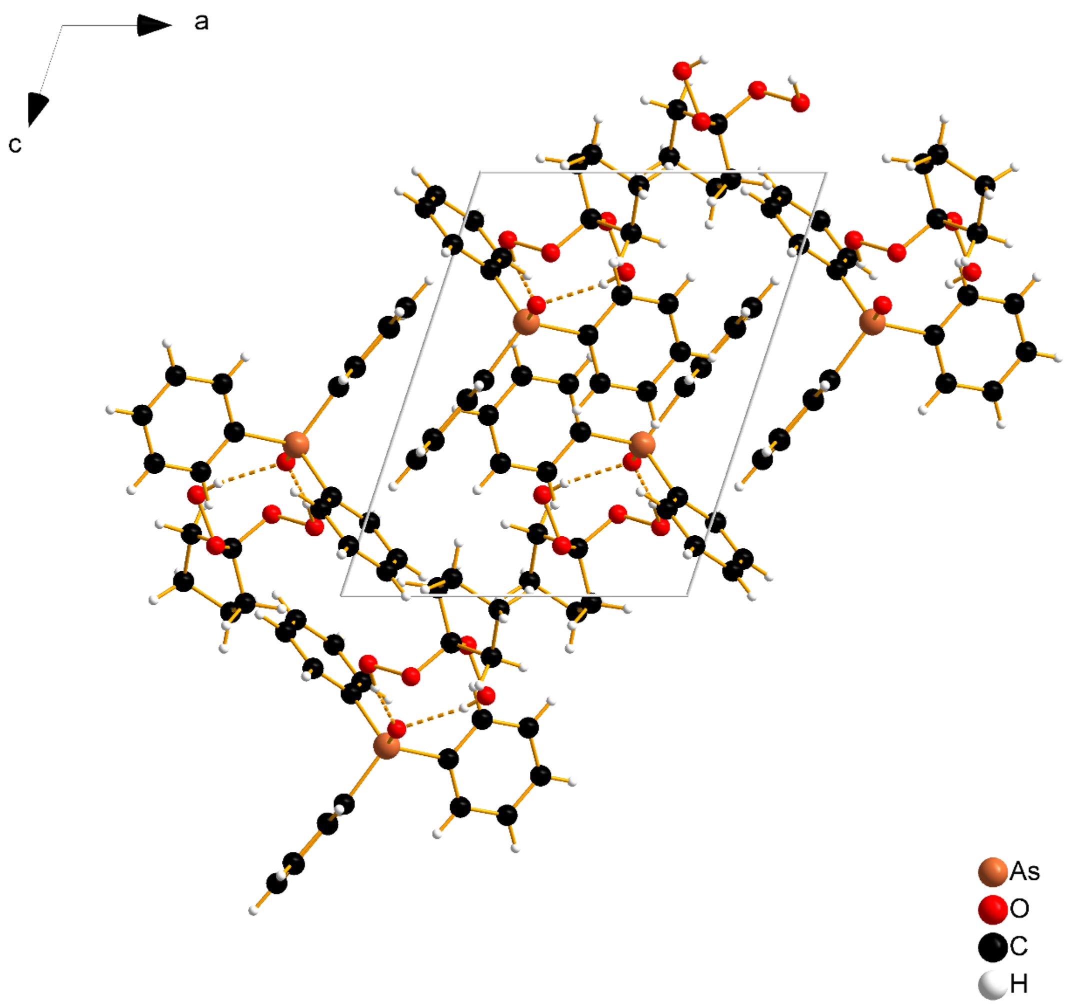

Figure S30. The crystal packing and the unit cell of the **5a** crystal structure viewed along the *b*-crystallographic axis.

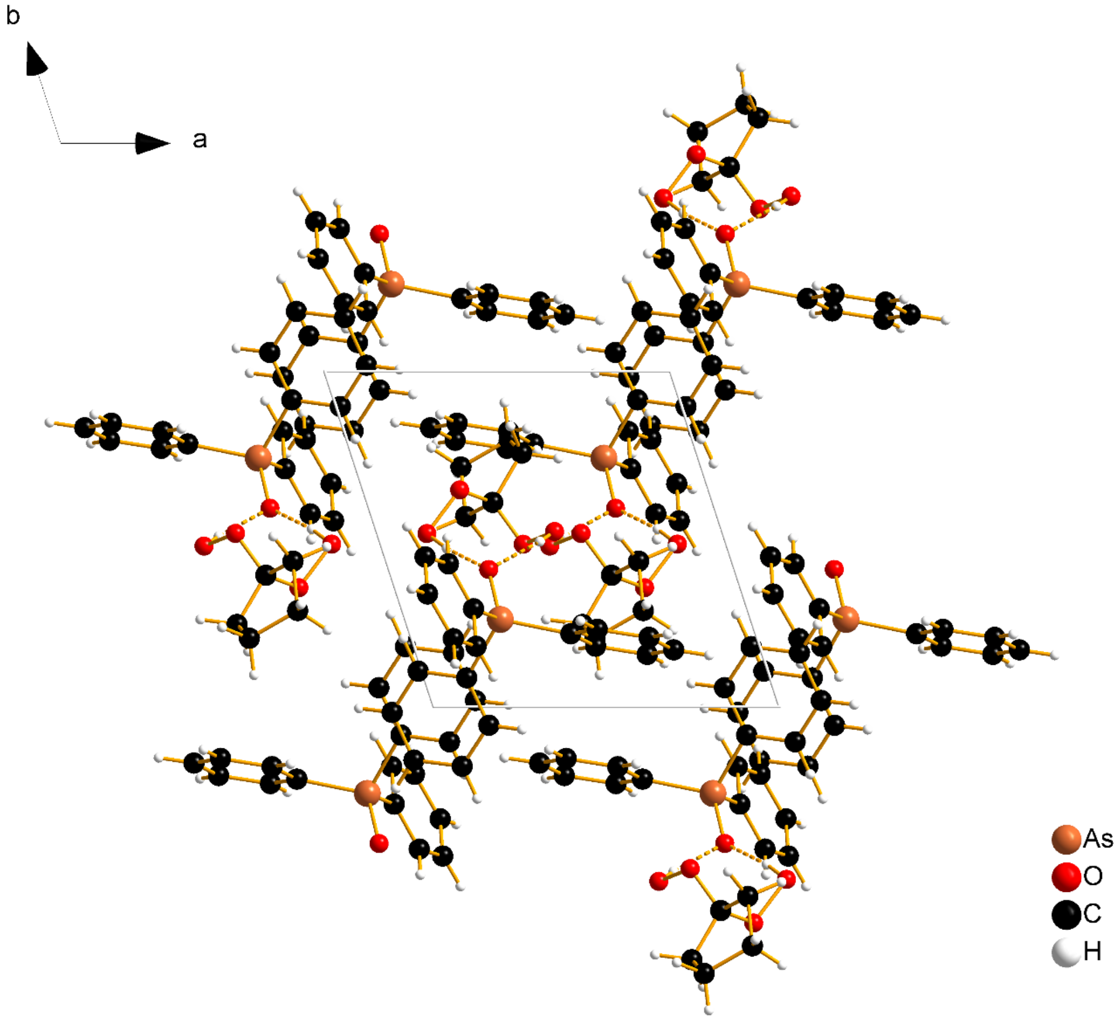

Figure S31. The crystal packing and the unit cell of the **5a** crystal structure viewed along the *c*-crystallographic axis.

$Ph_3AsO \cdot (HOO)_2(4\text{-}^t Bu\text{-}c\text{-}C_6H_9)$ (**5b**)

The crystal structure was measured at 100 K and it was determined that the compound crystallizes in the $P2_1/c$ space group. The high quality of the data allowed for the free refinement of all hydrogen atoms (positions and isotropic displacement parameters); the detailed hydrogen-bond parameters are provided below (Table S2, page S56).

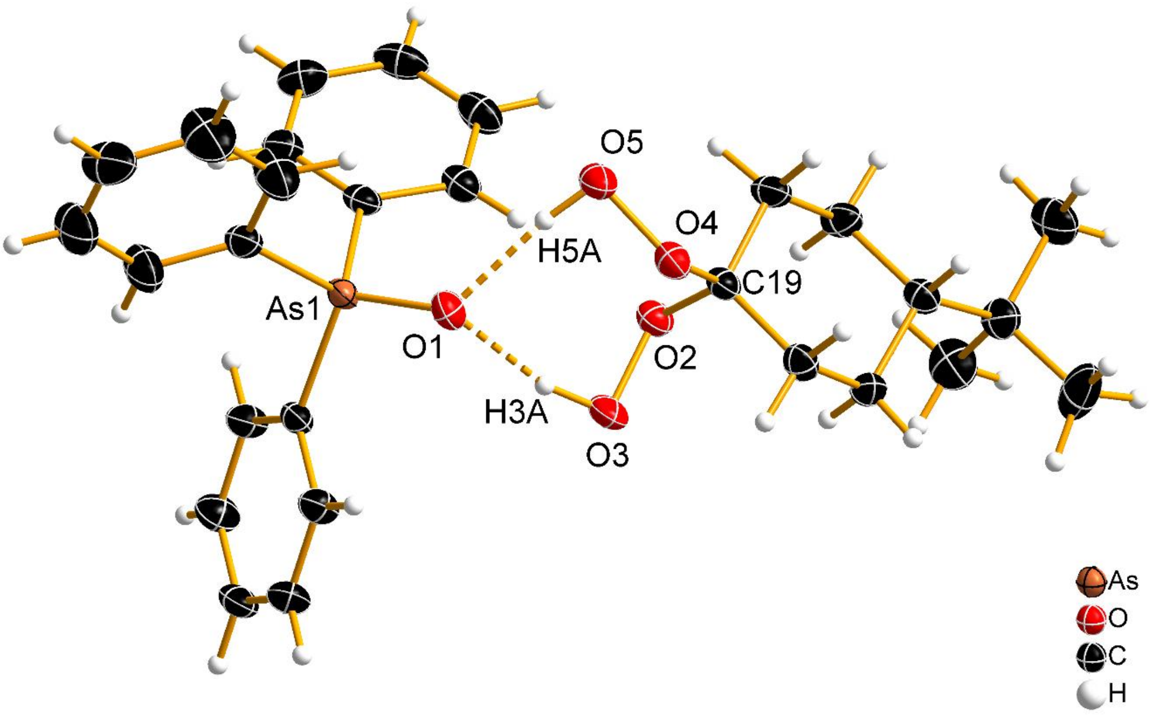

Figure S32. The asymmetric unit and selected atom labels of the **5b** crystal structure. Displacement ellipsoids are depicted at the 50% probability level and hydrogen atoms are shown as small spheres of arbitrary radius.

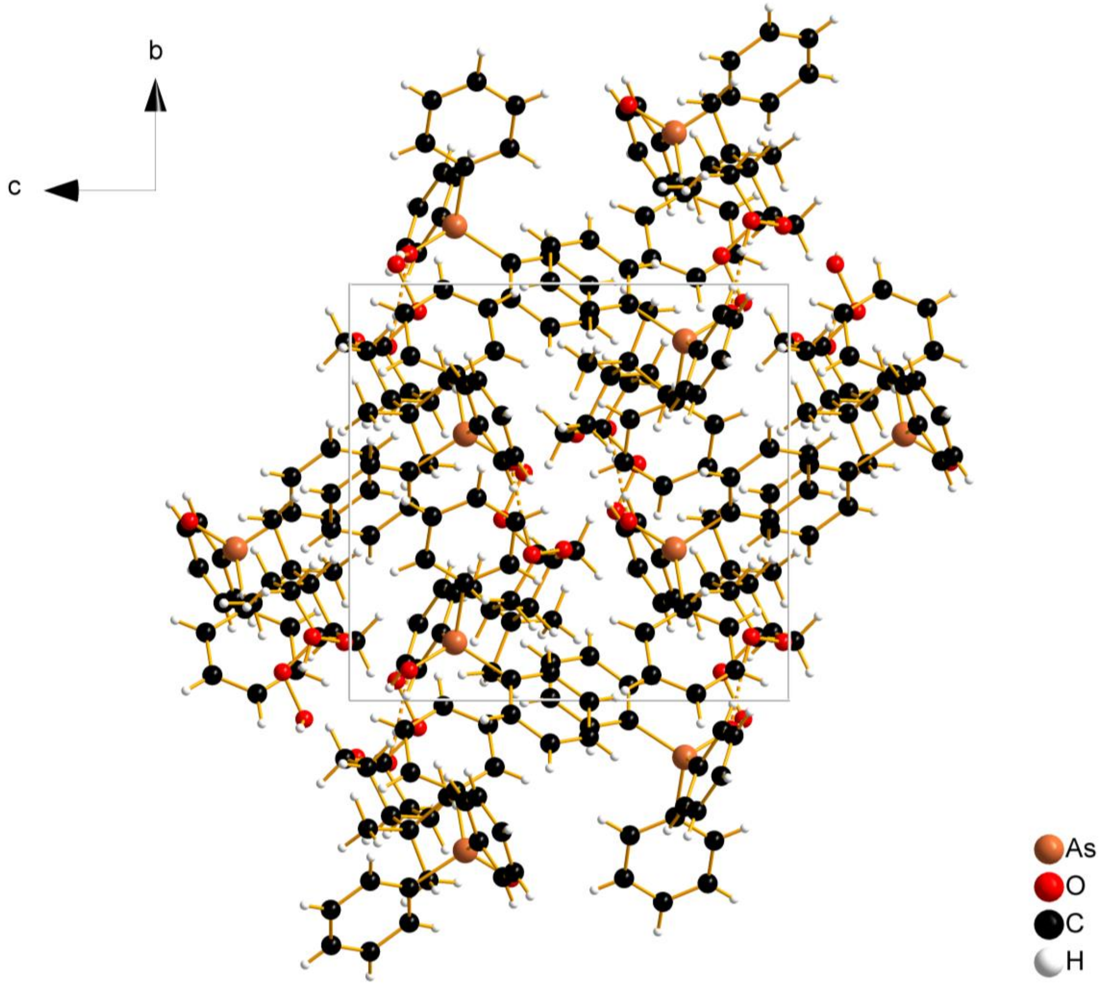

Figure S33. The crystal packing and the unit cell of the **5b** crystal structure viewed along the *a*-crystallographic axis.

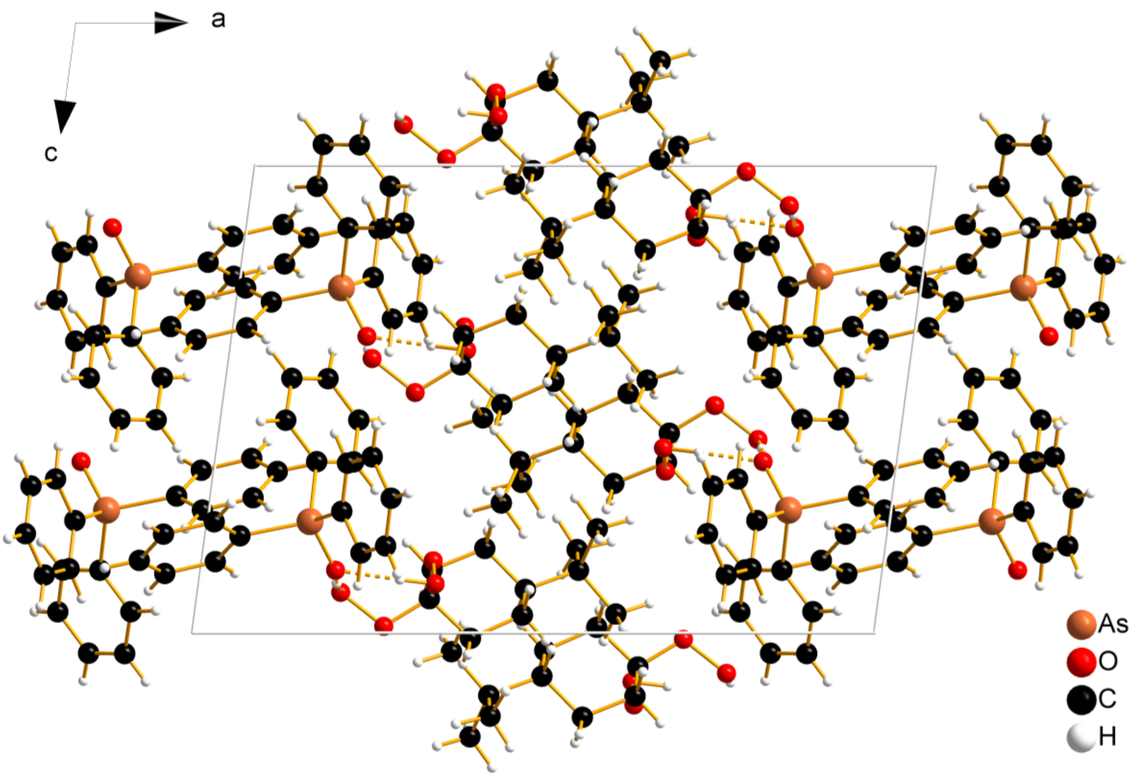

Figure S34. The crystal packing and the unit cell of the **5b** crystal structure viewed along the *b*-crystallographic axis.

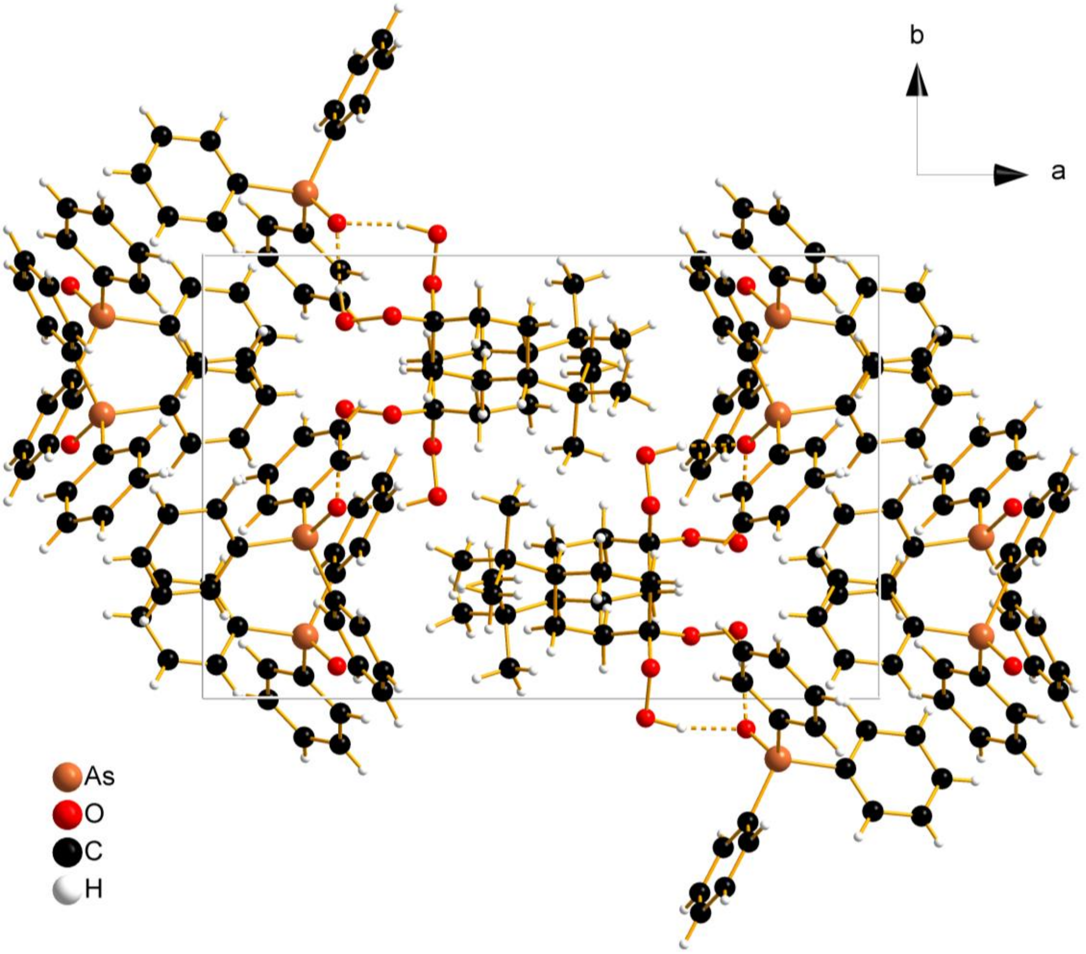

Figure S35. The crystal packing and the unit cell of the **5b** crystal structure viewed along the *c*-crystallographic axis.

$Ph_3AsO·(HOO)_2(c\text{-}C_7H_{12})$ (**5c**)

The crystal structure was measured at 200 K due to the destructive phase transition when cooling to 100 K. The compound crystallizes in the $P2_1/c$ space group. The hydrogen atoms were placed at the calculated positions with the exception of hydrogen atoms involved in the hydrogen bonds, which were freely refined (positions and isotropic displacement parameters). The detailed hydrogen bonds parameters are provided below (Table S2, page S56).

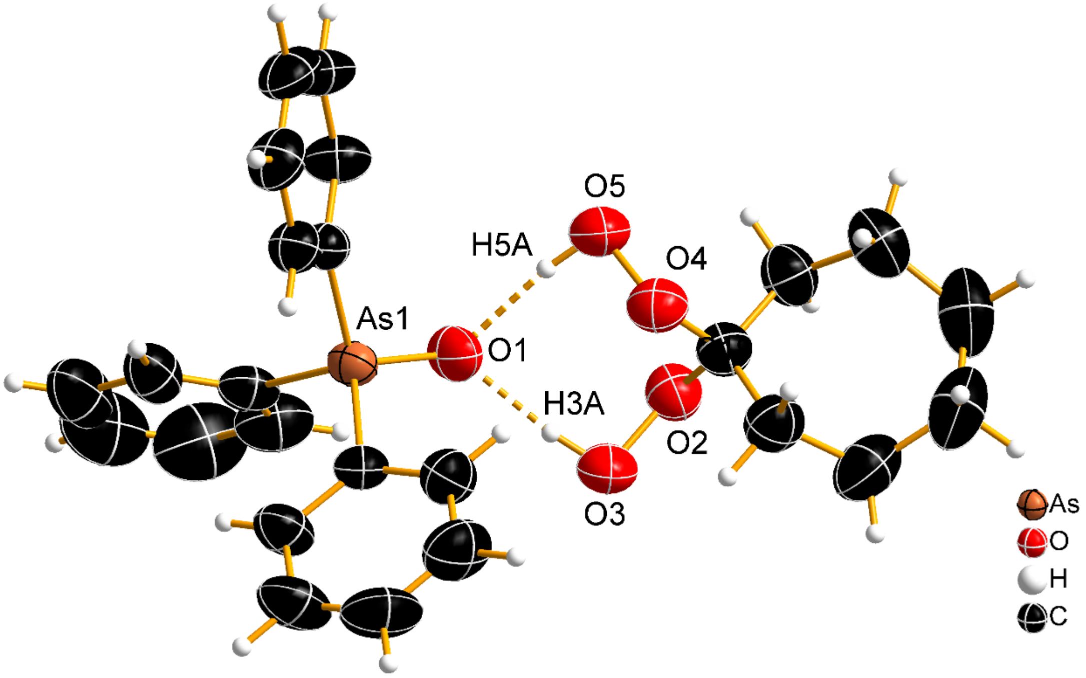

Figure S36. The asymmetric unit and selected atom labels of the **5c** crystal structure. Displacement ellipsoids are depicted at the 50% probability level and hydrogen atoms are shown as small spheres of arbitrary radius.

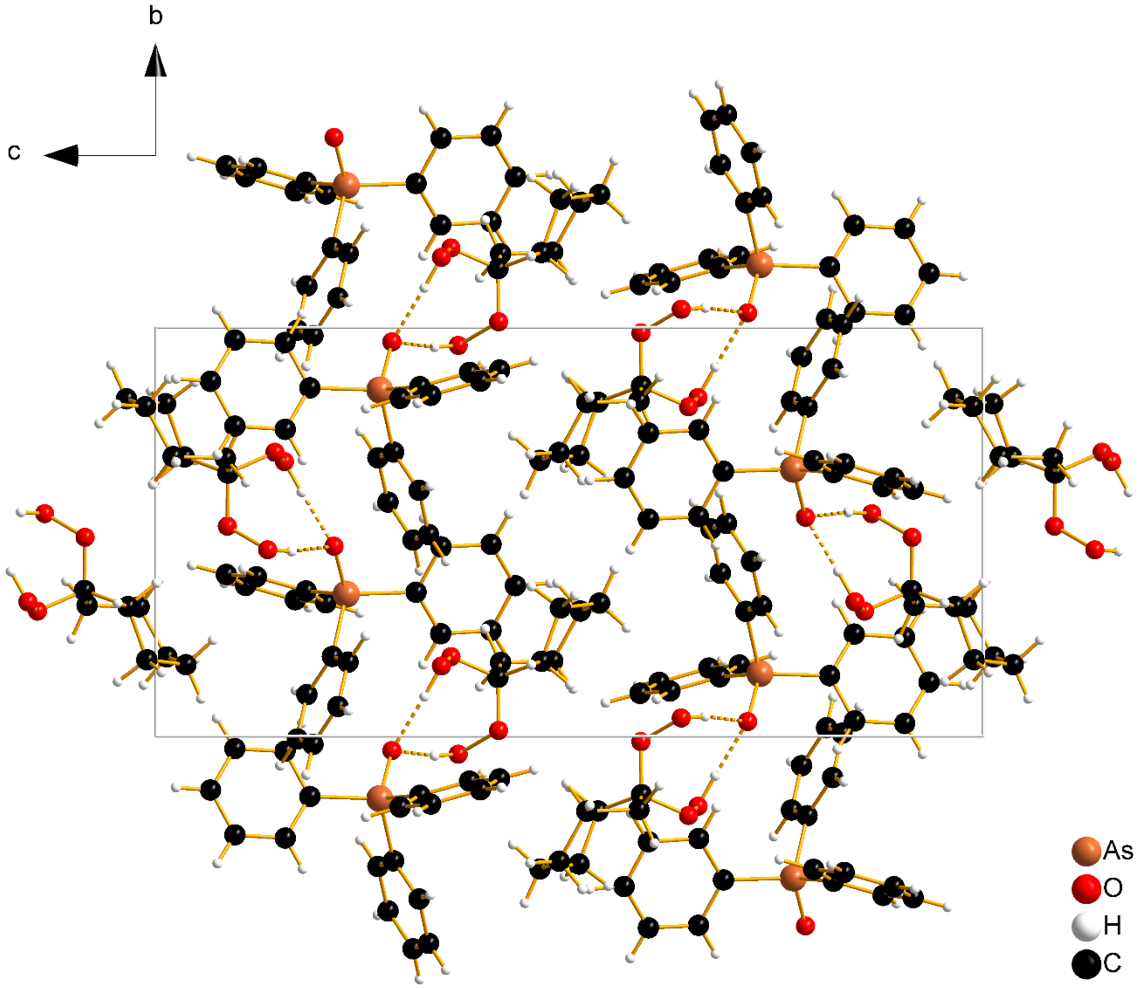

Figure S37. The crystal packing and the unit cell of the **5c** crystal structure viewed along the *a*-crystallographic axis.

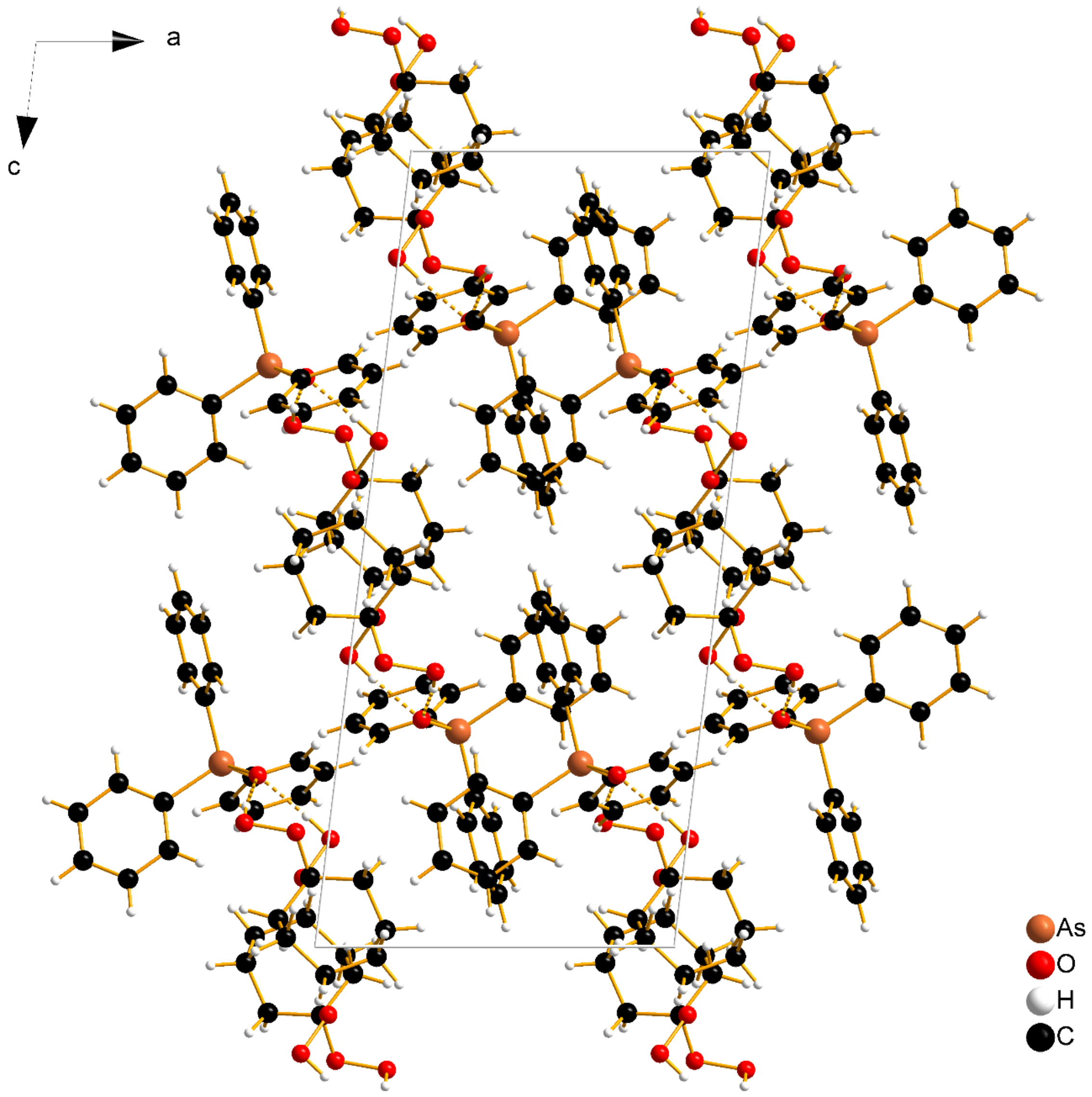

Figure S38. The crystal packing and the unit cell of the **5c** crystal structure viewed along the *b*-crystallographic axis.

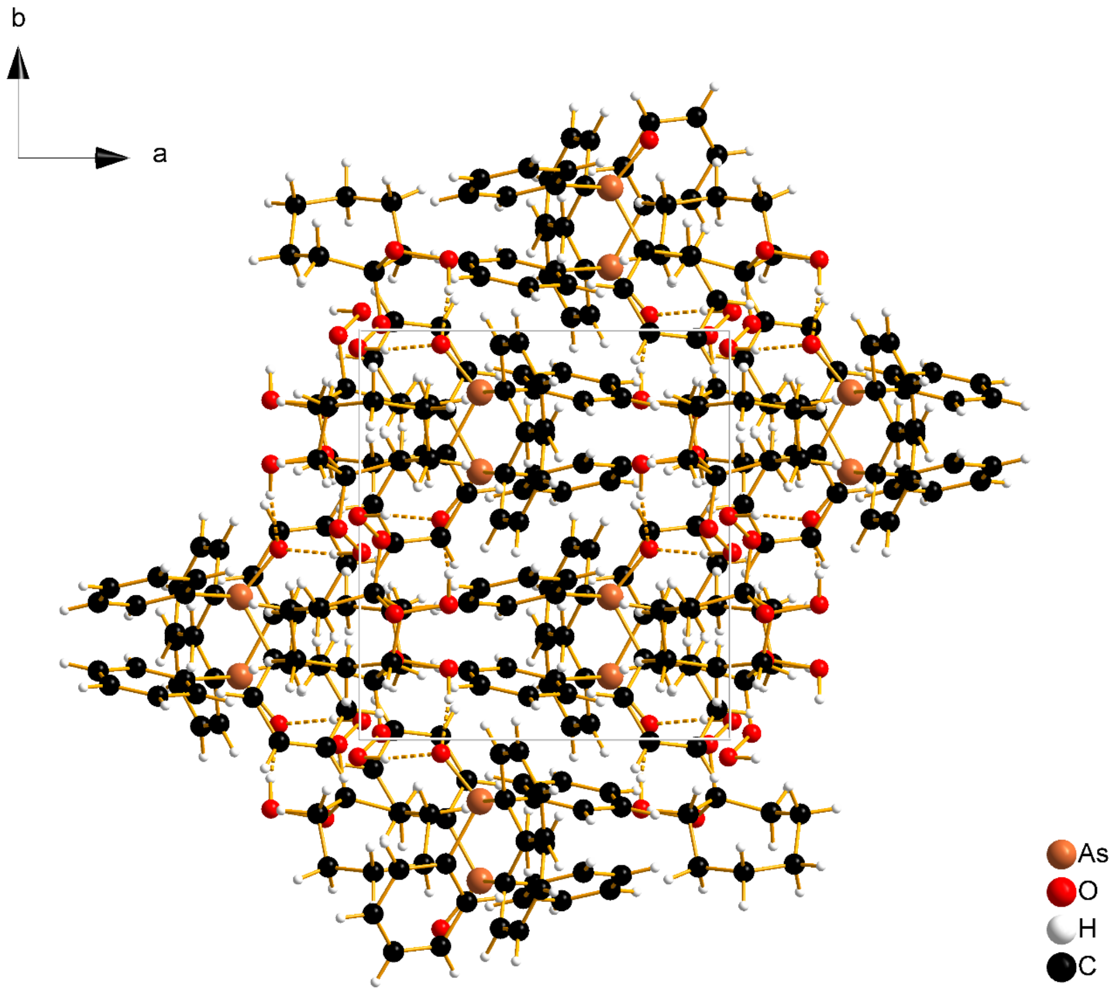

Figure S39. The crystal packing and the unit cell of the **5c** crystal structure viewed along the *c*-crystallographic axis.

Ph$_3$AsO·(HOO)$_2$(c-C$_{12}$H$_{22}$) (**5d**)

The crystal structure was measured at 200 K due to the destructive phase transition when cooling to 100 K. The compound crystallizes in the $P2_1/n$ space group. The hydrogen atoms were placed at the calculated positions with the exception of hydrogen atoms involved in the hydrogen bonds, which were freely refined (positions and isotropic displacement parameters). The detailed hydrogen-bond parameters are provided below (Table S2, page S56).

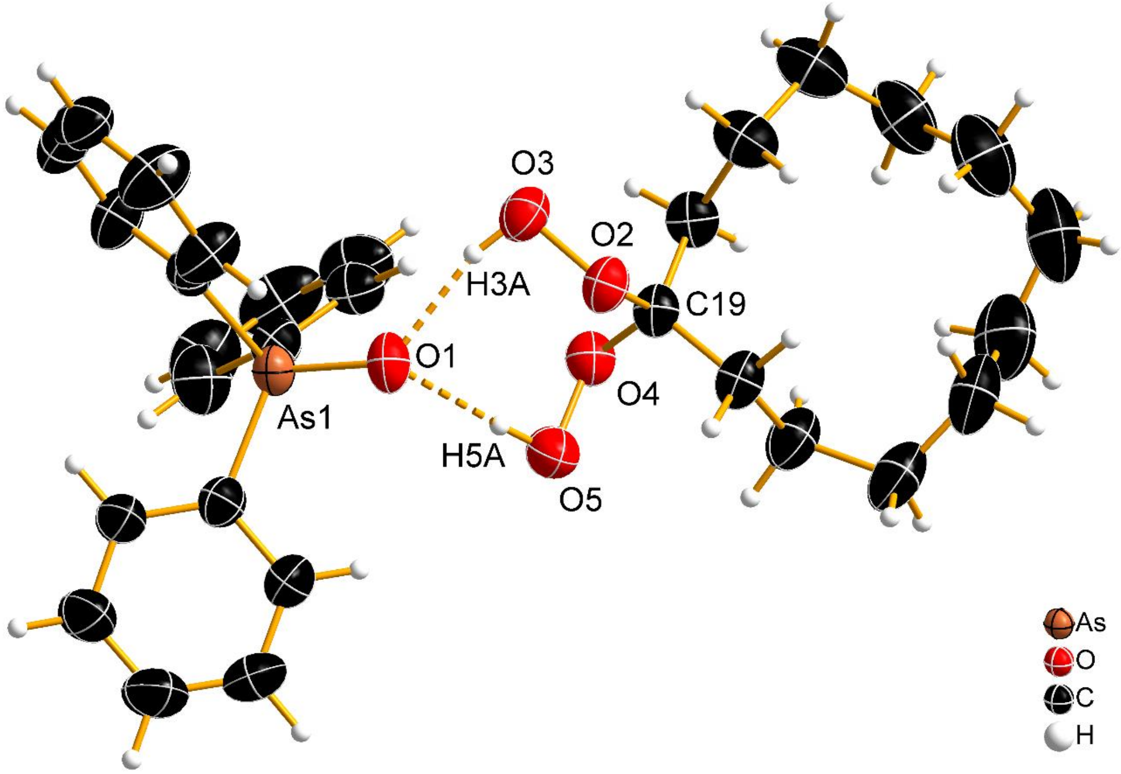

Figure S40. The asymmetric unit and selected atom labels of the **5d** crystal structure. Displacement ellipsoids are depicted at the 50% probability level and hydrogen atoms are shown as small spheres of arbitrary radius.

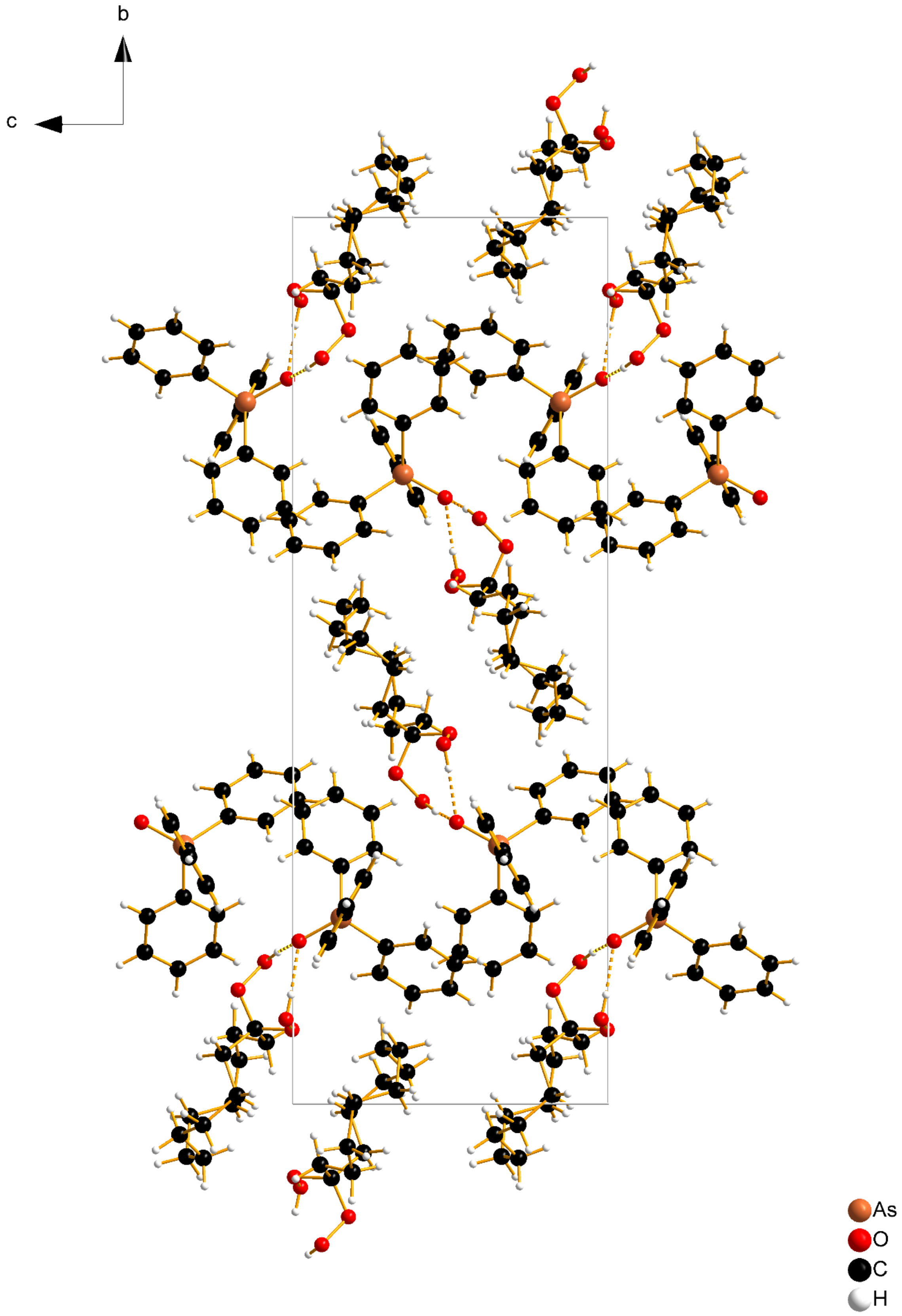

Figure S41. The crystal packing and the unit cell of the **5d** crystal structure viewed along the *a*-crystallographic axis.

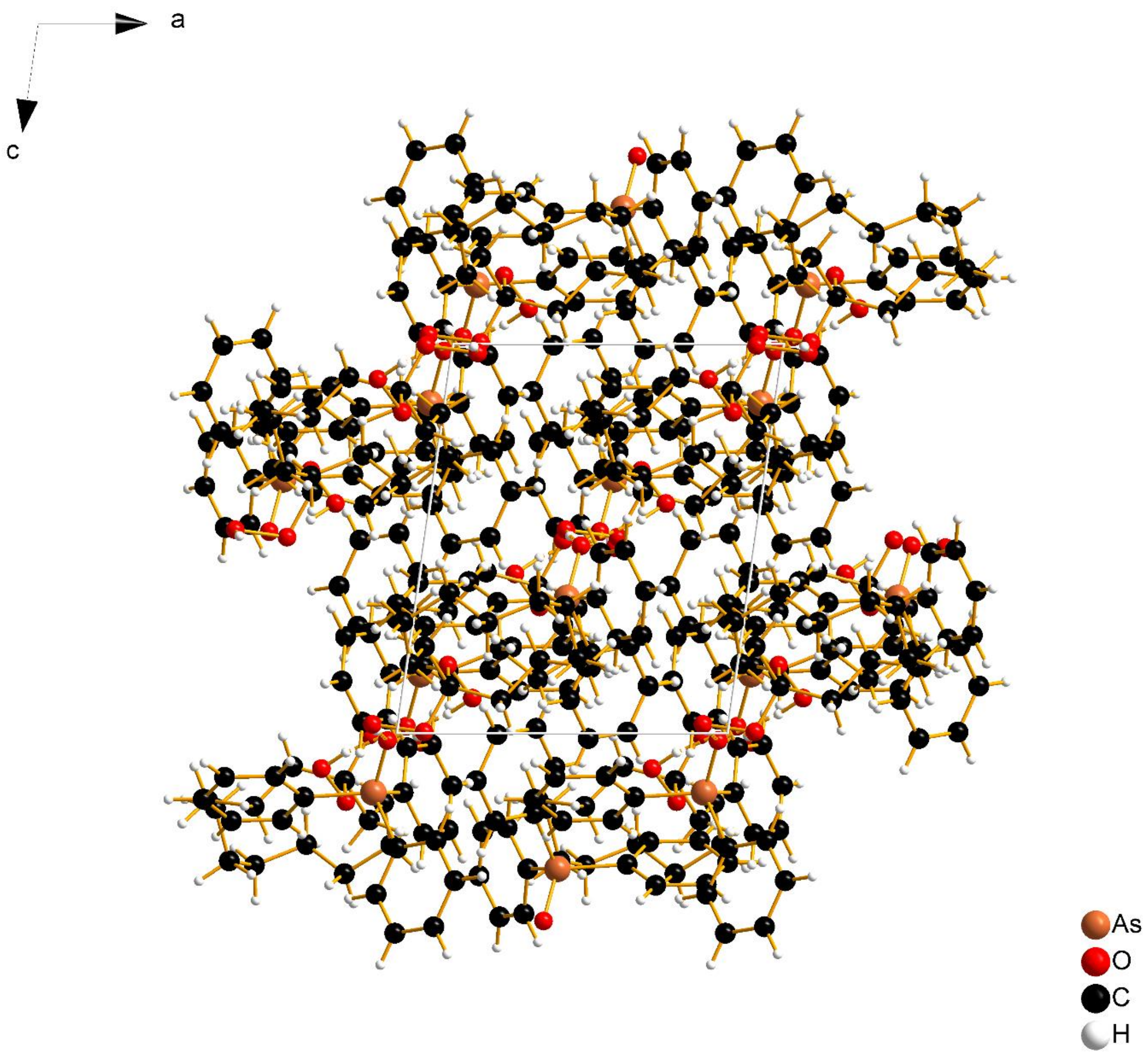

Figure S42. The crystal packing and the unit cell of the **5d** crystal structure viewed along the *b*-crystallographic axis.

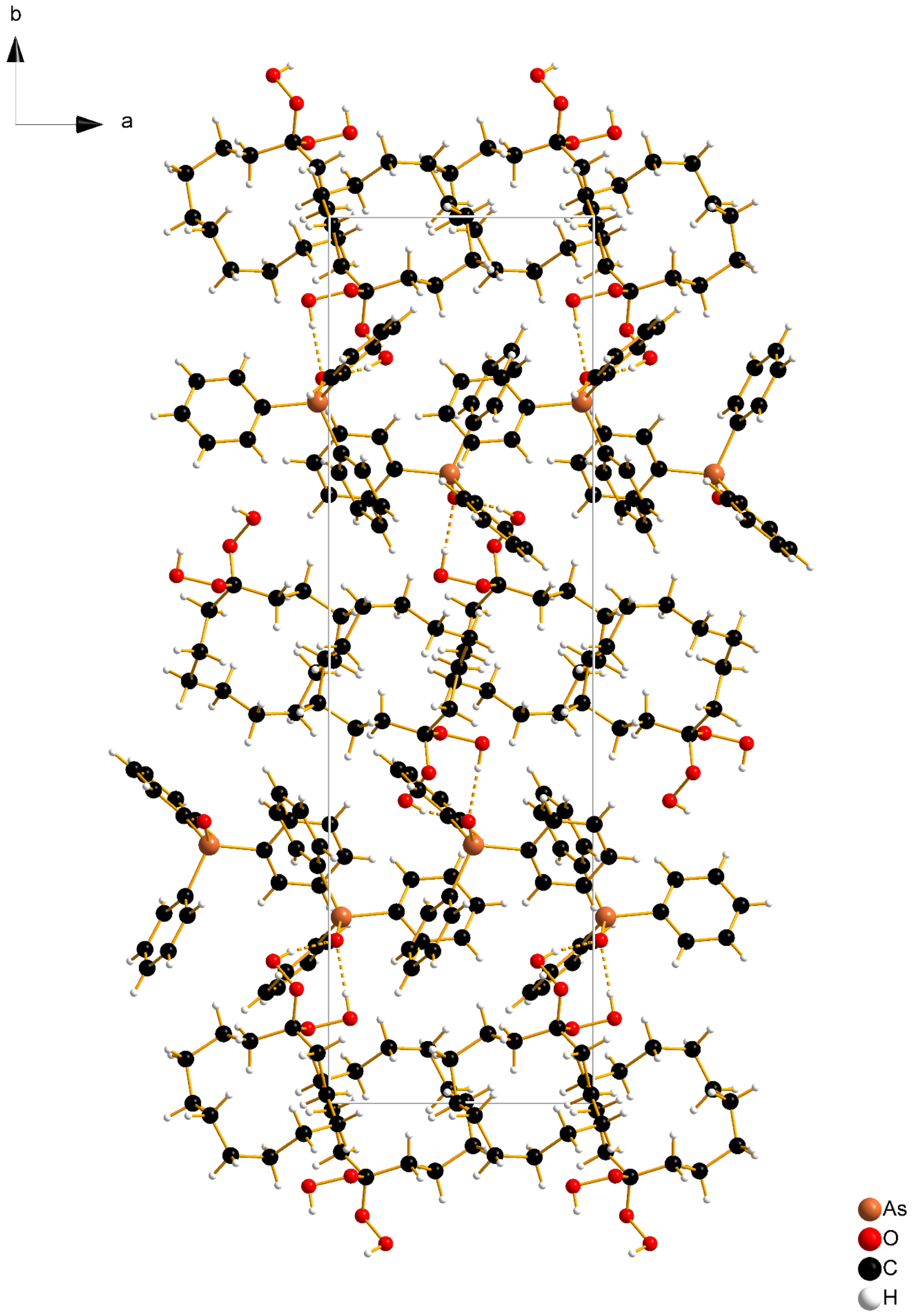

Figure S43. The crystal packing and the unit cell of the **5d** crystal structure viewed along the *c*-crystallographic axis.

Ph$_3$AsO·(HOO)$_2$(adm) (**5e**)

The crystal structure was measured at 100 K. The compound crystallizes in the $P2_1/n$ space group. There are two hydrogen-bonded adducts in the asymmetric unit. There is rotational disorder of one of the dhp molecules in the asymmetric unit with partial occupancies of 0.794(3) and 0.206(3). The hydrogen atoms were placed at the calculated positions with the exception of hydrogen atoms in main domains involved in the hydrogen bonds, which were freely refined (positions and isotropic displacement parameters). The refinement of the hydrogen atoms of a minor domain that participate in the hydrogen bonding resulted in unstable refinement. The detailed hydrogen-bond parameters are provided below (Table S2, page S56).

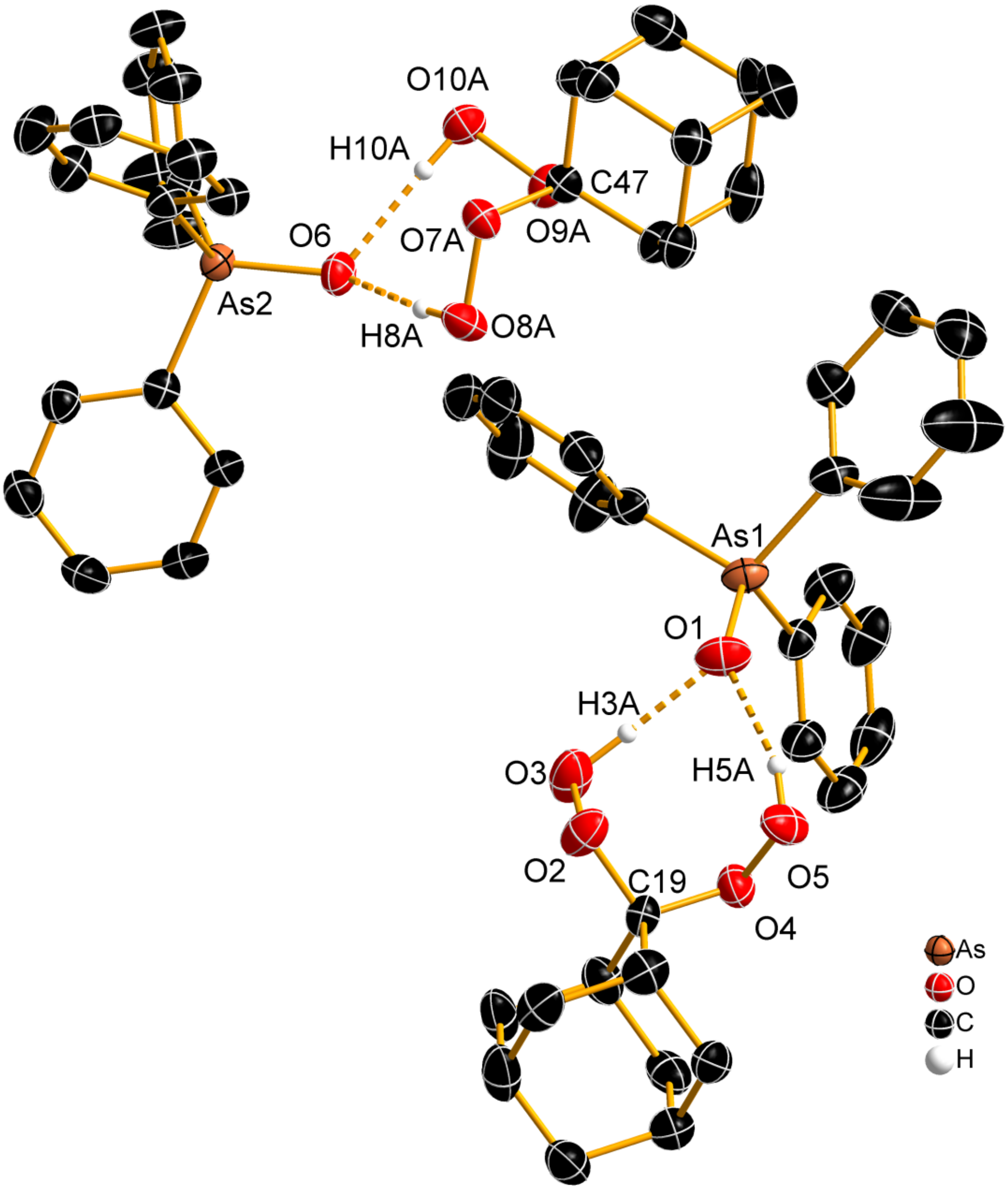

Figure S44. The asymmetric unit and selected atom labels of the **5e** crystal structure. Displacement ellipsoids are depicted at the 50% probability level. Hydrogen atoms are omitted for clarity, except for hydrogen atoms involved in hydrogen-bonding, which are shown as small spheres of arbitrary radius. Disordered atoms are omitted for clarity, only the main domain is shown.

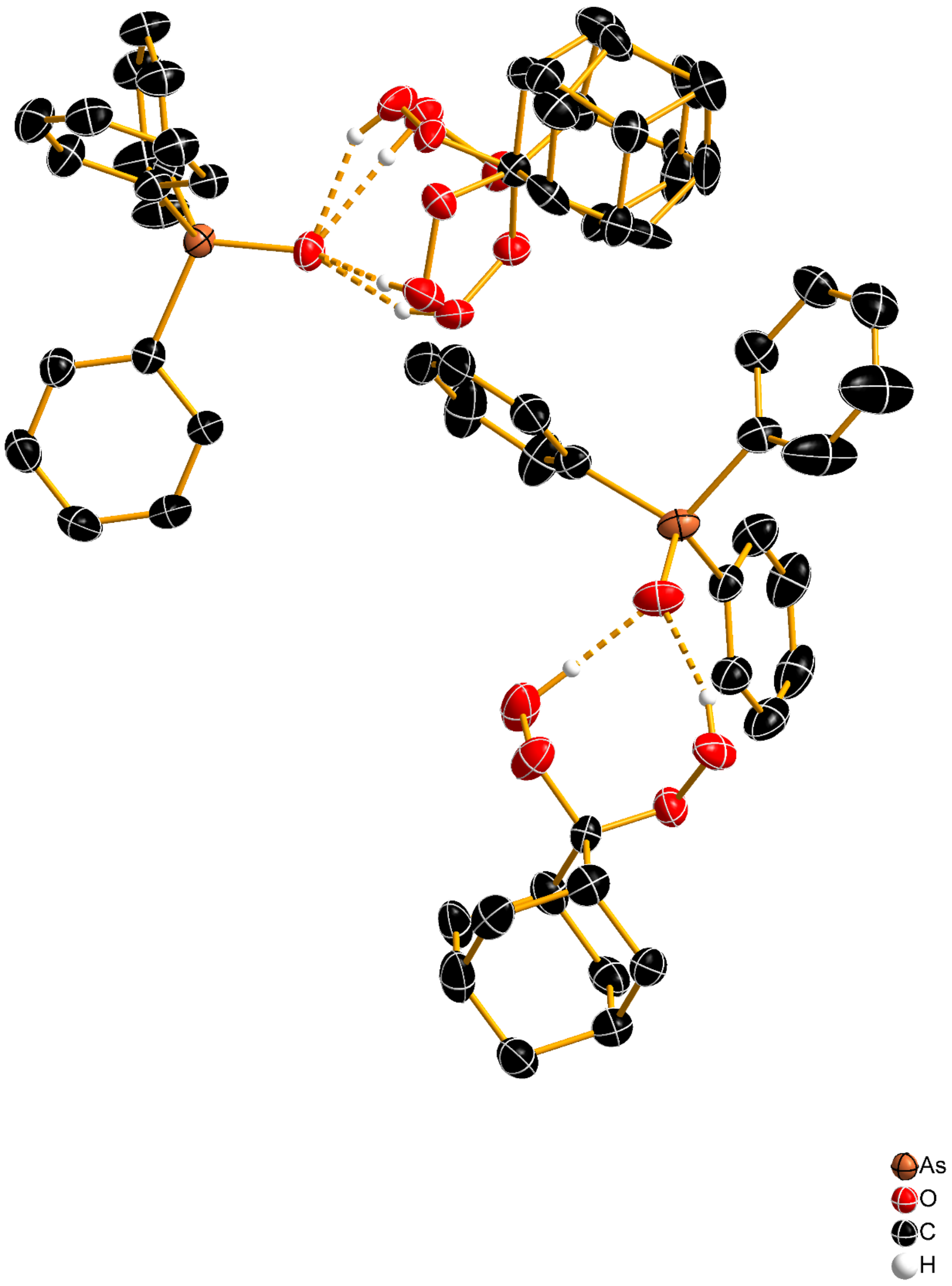

Figure S45. The asymmetric unit of the **5e** crystal structure with displayed disordered atoms. Displacement ellipsoids are depicted at the 50% probability level. Hydrogen atoms are omitted for clarity, except for hydrogen atoms involved in hydrogen-bonding, which are shown as small spheres of arbitrary radius.

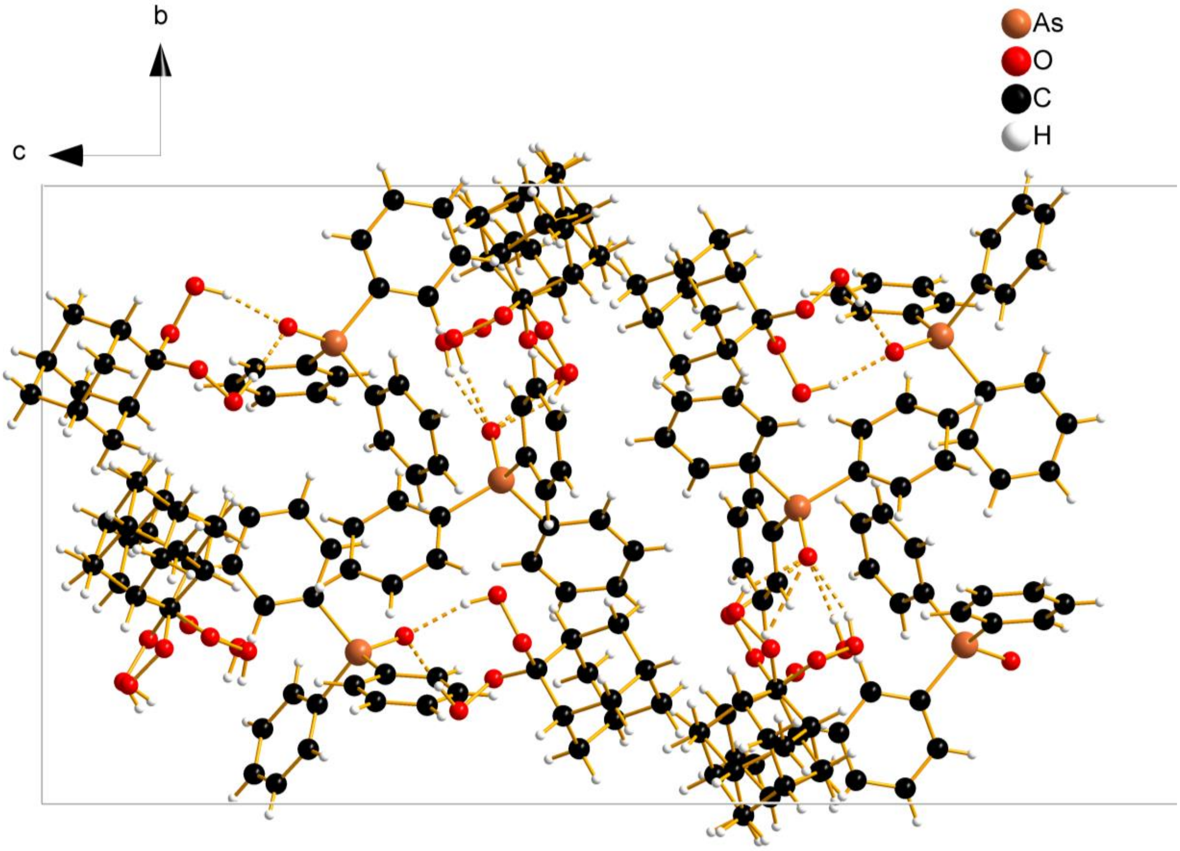

Figure S46. The crystal packing and the unit cell of the **5e** crystal structure viewed along the *a*-crystallographic axis. Atoms are shown as small spheres of arbitrary radius.

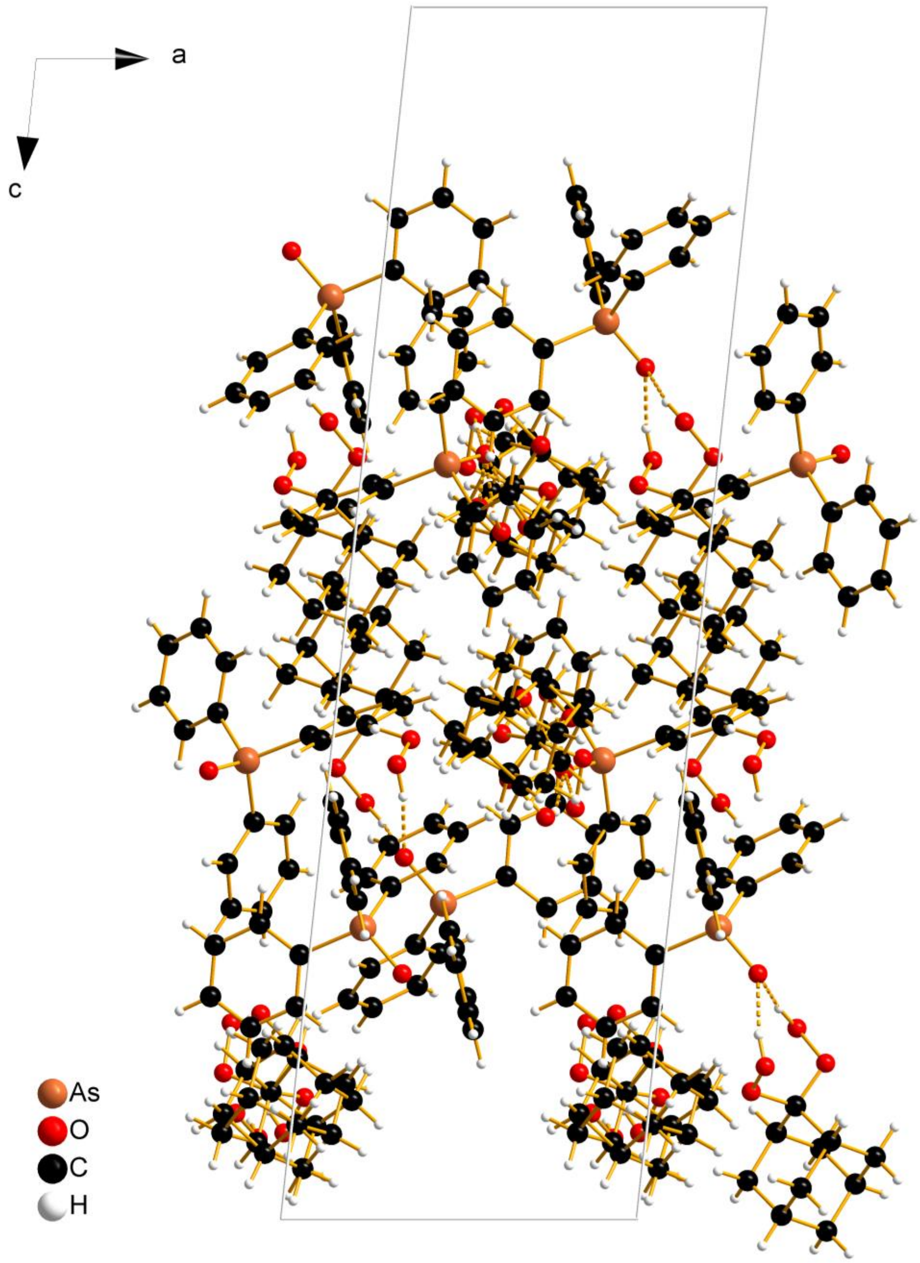

Figure S47. The crystal packing and the unit cell of the **5e** crystal structure viewed along the *b*-crystallographic axis.

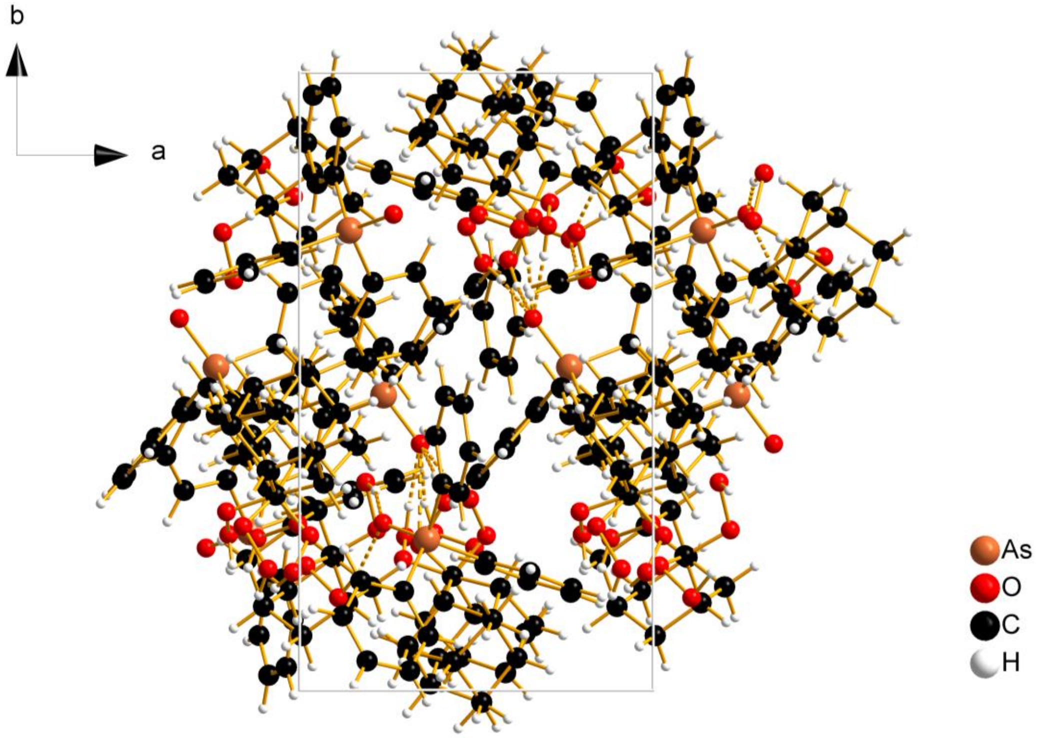

Figure S48. The crystal packing and the unit cell of the **5e** crystal structure viewed along the *c*-crystallographic axis.

Hydrogen-bond geometry in cocrystals **3** and **5a–e**

Table S2. Hydrogen-bond lengths and angles in cocrystals of Ph_3AsO with H_2O_2 and dhp.

	D−H···A	D−H [Å]	H···A [Å]	D···A [Å]	D−H···A [°]
**3**	O2−H2A···O1	0.96(3)	1.71(3)	2.655(2)	170(3)
	O3−H3A···O1	0.94(3)	1.69(3)	2.627(2)	175(3)
	O4−H4A···O2[a]	0.88(4)	1.86(4)	2.733(3)	171(4)
**5a**	O3−H3A···O1	0.98(6)	1.71(6)	2.681(4)	172(5)
	O5−H5A···O1	0.72(8)	1.98(8)	2.691(4)	169(9)
**5b**	O3−H3A···O1	0.98(3)	1.73(3)	2.6885(16)	166(3)
	O5−H5A···O1	0.90(3)	1.79(3)	2.6850(16)	173(2)
**5c**	O3−H3A···O1	0.925(19)	1.71(2)	2.631(2)	172(4)
	O5−H5A···O1	0.919(18)	1.762(19)	2.675(2)	172(4)
**5d**	O3−H3A···O1	0.857(17)	1.833(19)	2.672(2)	166(3)
	O5−H5A···O1	0.849(18)	1.851(18)	2.699(2)	177(3)
**5e**	O3−H3A···O1	0.96(5)	1.71(5)	2.662(3)	169(4)
	O5−H5A···O1	0.89(4)	1.84(4)	2.706(3)	163(4)
	O8A−H8A···O6	0.86(5)	1.82(5)	2.678(3)	175(4)
	O10A−H10A···O6	0.84(5)	1.83(5)	2.665(4)	172(5)
	O8B−H8B···O6	0.84	1.94	2.718(10)	154.1
	O10B−H10B···O6	0.84	1.87	2.618(16)	148.0

[a] Bridging H_2O_2.

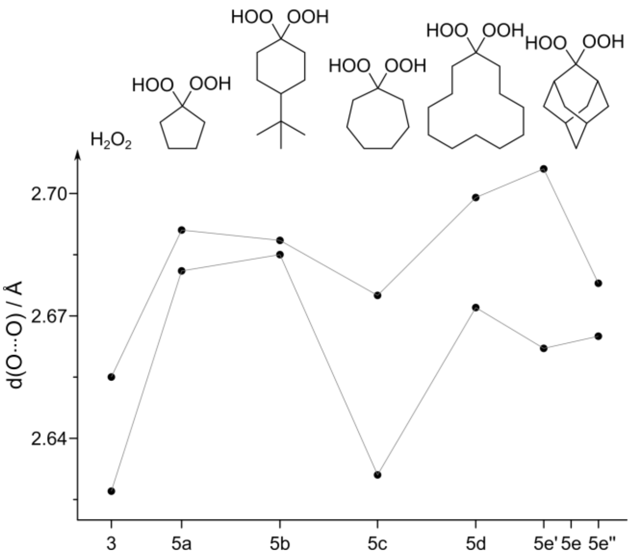

Figure S49. The O···O distances in hydrogen-bonded cocrystals.

Selected bond distances and angles

Table S3. Selected bond distances and angles in the crystal structure of Ph_3AsO (**2**).

Bond	Value [Å]
As1−O1	1.6527(11)
As1−C1	1.9273(16)
As1−C7	1.9263(16)
As1−C13	1.9272(15)

Angle	Value [°]
O1−As1−C1	110.08(6)
O1−As1−C7	110.19(6)
O1−As1−C13	109.12(6)
C1−As1−C13	107.68(7)
C7−As1−C1	111.55(6)
C7−As1−C13	108.12(7)
C2−C1−As1	114.47(12)
C6−C1−As1	125.03(12)

Table S4. Hydrogen-bond geometry in the crystal structure of **2**.

D−H···A	D−H [Å]	H···A [Å]	D···A [Å]	D−H···A [°]
C14A−H14A···O1	0.95	2.46	3.379(7)	163.1
C14B−H14B···O1	0.95	2.49	3.404(9)	161.7
C6−H6···O1	0.96(2)	2.48(2)	3.396(2)	158.8(16)
C12−H12···O1	0.99(2)	2.51(2)	3.464(2)	163.3(16)

Table S5. Selected bond distances and angles in the crystal structure of **3**.

Bond	Value [Å]
As1−O1	1.6670(12)
As1−C1	1.9165(18)
As1−C7	1.9174(18)
As1−C13	1.9156(18)
O2−H2A	0.96(3)
O2−O3	1.429(2)
O3−H3A	0.94(3)
O4−O4	1.464(4)
O4−H4A	0.88(4)

Angle	Value [°]
O1−As1−C1	109.65(7)
O1−As1−C7	109.41(7)
O1−As1−C13	111.32(7)
C1−As1−C7	109.93(8)
C13−As1−C1	108.40(8)
C13−As1−C7	108.11(8)
C2−C1−As1	117.99(14)
C6−C1−As1	121.03(14)
C8−C7−As1	120.56(15)
C12−C7−As1	118.50(14)
C14−C13−As1	119.76(15)
C18−C13−As1	119.75(15)
O3−O2−H2A	98.8(18)
O2−O3−H3A	100.3(18)
O4−O4−H4A	96(2)

Table S6. Hydrogen-bond geometry in the crystal structure of **3**.

D−H···A	D−H [Å]	H···A [Å]	D···A [Å]	D−H···A [°]
O2−H2A···O1	0.96(3)	1.71(3)	2.655(2)	170(3)
O3−H3A···O1	0.94(3)	1.69(3)	2.627(2)	175(3)
O4−H4A···O3	0.88(4)	1.86(4)	2.733(3)	171(4)

Table S7. Selected bond distances and angles in the crystal structure of **5a**.

Bond	Value [Å]
As1−O1	1.657(2)
As1−C1	1.925(3)
As1−C7	1.914(3)
As1−C13	1.916(3)
O2−O3	1.477(4)
O2−C19	1.422(4)
O3−H3A	0.98(6)
O4−O5	1.463(4)
O4−C19	1.412(4)
O5−H5A	0.72(8)

Angle	Value [°]
O1−As1−C1	110.45(13)
O1−As1−C7	111.79(13)
O1−As1−C13	109.25(13)
C7−As1−C1	108.22(14)
C7−As1−C13	110.46(14)
C13−As1−C1	106.53(14)
C2−C1−As1	121.9(3)
C6−C1−As1	117.4(3)
C8−C7−As1	117.8(3)
C12−C7−As1	121.4(3)
C14−C13−As1	120.9(3)
C18−C13−As1	118.0(3)
C19−O2−O3	109.3(2)
O2−O3−H3A	98(3)
C19−O4−O5	109.0(3)
O4−O5−H5A	102(7)
O2−C19−C20	113.5(3)
O2−C19−C23	104.1(3)
O4−C19−O2	111.5(3)
O4−C19−C20	105.8(3)
O4−C19−C23	115.1(3)

Table S8. Hydrogen-bond geometry in the crystal structure of **5a**.

D−H···A	D−H [Å]	H···A [Å]	D···A [Å]	D−H···A [°]
O3−H3A···O1	0.98(6)	1.71(6)	2.681(4)	172(5)
O5−H5A···O1	0.72(8)	1.98(8)	2.691(4)	169(9)

Table S9. Selected bond distances and angles in the crystal structure of **5b**.

Bond	Value [Å]
As1−O1	1.6602(11)
As1−C1	1.9066(14)
As1−C7	1.9158(15)
As1−C13	1.9175(16)
O2−O3	1.4637(15)
O2−C19	1.4164(18)
O3−H3A	0.98(3)
O4−O5	1.4657(17)
O4−C19	1.4246(18)
O5−H5A	0.90(3)

Angle	Value [°]
O1−As1−C1	110.72(6)
O1−As1−C7	110.34(6)
O1−As1−C13	107.94(6)
C1−As1−C7	108.91(6)
C1−As1−C13	109.86(6)
C7−As1−C13	109.05(6)
C2−C1−As1	120.93(11)
C6−C1−As1	118.34(11)
C8−C7−As1	122.04(12)
C12−C7−As1	116.84(12)
C14−C13−As1	117.35(13)
C18−C13−As1	122.67(13)
C19−O2−O3	109.74(10)
O2−O3−H3A	98.8(17)
C19−O4−O5	109.68(11)
O4−O5−H5A	99.2(16)
O2−C19−O4	111.08(11)
O2−C19−C20	113.01(12)
O2−C19−C24	103.70(12)
O4−C19−C20	104.26(12)
O4−C19−C24	112.86(12)

Table S10. Hydrogen-bond geometry in the crystal structure of **5b**.

D−H···A	D−H [Å]	H···A [Å]	D···A [Å]	D−H···A [°]
O3−H3A···O1	0.98(3)	1.73(3)	2.6885(16)	166(3)
O5−H5A···O1	0.90(3)	1.79(3)	2.6850(16)	173(2)

Table S11. Selected bond distances and angles in the crystal structure of **5c**.

Bond	Value [Å]
As1−O1	1.6584(14)
As1−C1	1.9024(19)
As1−C7	1.9177(19)
As1−C13	1.910(2)
O2−O3	1.464(2)
O2−C19	1.421(3)
O3−H3A	0.925(19)
O4−O5	1.455(2)
O4−C19	1.426(3)
O5−H5A	0.919(18)

Angle	Value [°]
O1−As1−C1	110.82(8)
O1−As1−C7	111.18(8)
O1−As1−C13	108.06(9)
C1−As1−C7	108.05(8)
C1−As1−C13	108.63(9)
C13−As1−C7	110.08(9)
C19−O2−O3	110.87(16)
O2−O3−H3A	101(3)
C19−O4−O5	110.47(16)
O4−O5−H5A	99(2)
C2−C1−As1	117.93(16)
C6−C1−As1	121.55(15)
C8−C7−As1	121.62(16)
C12−C7−As1	117.17(16)
C14−C13−As1	121.64(17)
C18−C13−As1	117.0(2)
O2−C19−O4	110.80(18)
O2−C19−C20	101.92(18)
O2−C19−C25	113.50(19)
O4−C19−C20	112.83(19)
O4−C19−C25	101.14(18)

Table S12. Hydrogen-bond geometry in the crystal structure of **5c**.

D−H···A	D−H [Å]	H···A [Å]	D···A [Å]	D−H···A [°]
O3−H3A···O1	0.925(19)	1.71(2)	2.631(2)	172(4)
O5−H5A···O1	0.919(18)	1.762(19)	2.675(2)	172(4)

Table S13. Selected bond distances and angles in the crystal structure of **5d**.

Bond	Value [Å]
As1−O1	1.6598(13)
As1−C1	1.9143(19)
As1−C7	1.908(2)
As1−C13	1.904(2)
O2−O3	1.4614(19)
O2−C19	1.423(2)
O3−H3A	0.857(17)
O4−O5	1.464(2)
O4−C19	1.418(2)
O5−H5A	0.849(18)

Angle	Value [°]
O1−As1−C1	109.27(8)
O1−As1−C7	110.75(8)
O1−As1−C13	109.07(8)
C7−As1−C1	108.86(8)
C13−As1−C1	109.52(8)
C13−As1−C7	109.35(9)
C2−C1−As1	117.00(15)
C6−C1−As1	123.05(16)
C8−C7−As1	121.28(17)
C12−C7−As1	117.73(16)
C14−C13−As1	117.61(16)
C18−C13−As1	121.82(15)
C19−O2−O3	109.29(13)
O2−O3−H3A	102(2)
C19−O4−O5	109.62(14)
O4−O5−H5A	94(2)
O2−C19−C20	102.32(15)
O2−C19−C30	113.13(16)
O4−C19−O2	111.15(15)
O4−C19−C20	113.11(16)
O4−C19−C30	102.68(15)

Table S14. Hydrogen-bond geometry in the crystal structure of **5d**.

D−H···A	D−H [Å]	H···A [Å]	D···A [Å]	D−H···A [°]
O3−H3A···O1	0.857(17)	1.833(19)	2.672(2)	166(3)
O5−H5A···O1	0.849(18)	1.851(18)	2.699(2)	177(4)

Table S15. Selected bond distances and angles in the crystal structure of **5e**.

Bond	Value [Å]
As1−O1	1.6631(19)
As1−C1	1.915(3)
As1−C7	1.911(3)
As1−C13	1.909(2)
As2−O6	1.6649(17)
As2−C29	1.916(2)
As2−C35	1.907(2)
As2−C41	1.909(3)
O2−O3	1.471(3)
O2−C19	1.416(3)
O3−H3A	0.96(5)
O4−O5	1.477(3)
O4−C19	1.416(3)
O5−H5A	0.89(4)
O7A−O8A	1.468(4)
O8A−H8A	0.86(5)
O9A−O10A	1.461(4)
O10A−H10A	0.84(5)
O7B−O8B	1.447(14)
O8B−H8B	0.8400
O9B−O10B	1.44(2)
O10B−H10B	0.8400

Angle	Value [°]
O1−As1−C1	110.90(11)
O1−As1−C7	111.26(11)
O1−As1−C13	110.47(11)
C7−As1−C1	104.91(12)
C13−As1−C1	109.61(12)
C13−As1−C7	109.54(11)
C2−C1−As1	120.5(2)
C2−C1−C6	120.3(3)
C6−C1−As1	119.2(2)
C8−C7−As1	117.0(2)
C12−C7−As1	123.2(2)
C14−C13−As1	117.5(2)
C18−C13−As1	121.3(2)
O6−As2−C29	109.71(10)
O6−As2−C35	110.80(10)
O6−As2−C41	108.87(10)
C35−As2−C29	109.20(10)
C35−As2−C41	106.74(10)
C41−As2−C29	111.48(10)
C30−C29−As2	117.64(19)
C34−C29−As2	121.68(19)
C36−C35−As2	121.3(2)
C40−C35−As2	118.55(19)
C42−C41−As2	121.68(19)
C46−C41−As2	118.2(2)
C19−O2−O3	108.87(18)

Angle	Value [°]
O2−O3−H3A	102(3)
C19−O4−O5	109.20(18)
O4−O5−H5A	99(2)
O2−C19−O4	110.0(2)
O2−C19−C20	114.1(2)
O2−C19−C26	103.9(2)
O4−C19−C20	104.20(19)
O4−C19−C26	114.7(2)
O7A−C47−C48A	114.0(2)
O7A−C47−C54A	103.1(2)
O9A−C47−O7A	111.1(2)
O9A−C47−C48A	106.1(2)
O9A−C47−C54A	112.7(2)
O7B−C47−C48B	110.5(7)
O7B−C47−C54B	99.2(6)
O9B−C47−O7B	107.8(5)
O9B−C47−C48B	110.5(8)
O9B−C47−C54B	114.3(7)
C47−O7A−O8A	108.8(2)
O7A−O8A−H8A	96(3)
C47−O9A−O10A	110.0(2)
O9A−O10A−H10A	94(3)
O8B−O7B−C47	107.9(7)
O7B−O8B−H8B	109.5
C47−O9B−O10B	112.5(11)
O9B−O10B−H10B	109.5

Table S16. Hydrogen-bond geometry in the crystal structure of **5e**.

D−H···A	D−H [Å]	H···A [Å]	D···A [Å]	D−H···A [°]
O3−H3A···O1	0.96(5)	1.71(5)	2.662(3)	169(4)
O5−H5A···O1	0.89(4)	1.84(4)	2.706(3)	163(4)
O8A−H8A···O6	0.86(5)	1.82(5)	2.678(3)	175(4)
O10A−H10A···O6	0.84(5)	1.83(5)	2.665(4)	172(5)
O8B−H8B···O6	0.84	1.94	2.718(10)	154.1
O10B−H10B···O6	0.84	1.87	2.618(16)	148.0

Table S17. Comparison of As=O bond lengths in crystals **2**, **3**, and **5**.

Compound	*d*(As−O) [Å]	elongation [Å]
**2**	1.6528(12)	/
**3**	1.6670(12)	0.0142
**5a**	1.657(2)	0.0042
**5b**	1.6602(11)	0.0074
**5c**	1.6584(14)	0.0056
**5d**	1.6598(13)	0.007
**5e**	1.6631(19)<br>1.6649(17)	0.0103<br>0.0121

Table S18. Comparison of O−O bond lengths in crystals **3** and **5**.

Compound	*d*(O−O) [Å]
**3**	1.429(2)<br>1.464(4)
**5a**	1.477(4)<br>1.463(4)
**5b**	1.4637(15)<br>1.4657(17)
**5c**	1.464(2)<br>1.455(2)
**5d**	1.4614(19)<br>1.464(2)
**5e**	1.471(3)<br>1.477(3)<br>1.468(4)<br>1.461(4)<br>1.447(14)<br>1.44(2)

Literature reports of H_2O_2 crystal structure

Table S19. Geometrical parameters in the crystal structures of H_2O_2.

H_2O_2	XRD (110 K)[12]	Neutron (100 K)[12]	Neutron (253 K)[13]	XRD (253 K)[14]
**O−O [Å]**	1.461(3)	1.458(4)	1.453(7)	1.49(2)
**O−H [Å]**	0.77(9)	0.988(3)	0.988(5)	
**O···H [Å]**	1.98(10)	1.786(5)	1.825(6)	
**O···O [Å]**	2.758(6)	2.761(5)	2.799(8)	2.78
**∠O−O−H [°]**	99.5(18)	101.9(1)	102.7(3)	
**∠O−O···O [°]**	94.4(5)	94.5(3)	94.8(2)	93.9(15)
**torsion(H−O−O−H) [°]**	93(2)	90.2(4)	90.2(6)	

Statistics of the O−O bond lengths in the CSD database

Analysis of reported crystal structures in CSD database was performed.[15,16] Initially the compounds containing hydrogen peroxide were analyzed. The analysis of O−O bond lengths revealed the average value of 1.45(2) Å in 269 fragments of 160 crystal structures.

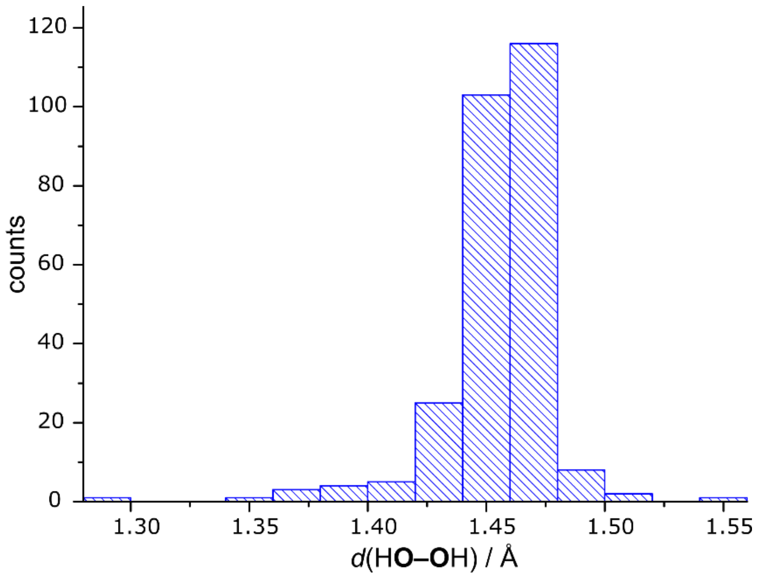

Bin center	Counts
1.29	1
1.31	0
1.33	0
1.35	1
1.37	3
1.39	4
1.41	5
1.43	25
1.45	103
1.47	116
1.49	8
1.51	2
1.53	0
1.55	1
1.57	0

Figure S50. Statistical analysis of H**O−O**H bonds in reported crystals structures in CSD containing hydrogen peroxide.

List of the crystal structure refcodes in CSD database that contain hydrogen peroxide:

ZOJWUU	ZOKLAQ	ZOKLEU	ZOKLIY	AMOXPH10	ANIMUH
ANINAO	ANINES	AVUDAZ	AYAQEZ	AYAQID	AZAMIZ
AZAMIZ01	BABGUJ	BAFGOH	BAFJUQ	BAMXAR	BIPHIV
BIPHOB	BIPXUX	BIPXUX01	BIPXUX02	BIPXUX03	BIPXUX04
BIPXUX05	BIPXUX06	BIPXUX07	BIPYIM	BIPYOS	BIPYUY
BIPZAF	BIPZEJ	BIPZIN	BIPZOT	BIPZUZ	BOHLOC
BONGES	BONGIW	CAZHAN	CAZHAN01	CAZHAN02	CAZHER
CAZHIV	CAZHOB	CAZHUH	CAZJAP	CELNIS	CEYXUZ
DATHIQ	DEKMIT	DOJMIZ	EDEDOK	EDEDUQ	FURFIH
GADMUU	GADOXP10	GAVDAM	GAVRUU	GAVSAB	GAVSEF
GAVSIJ	GAWKOI	GAWKUO	GICLAJ	HAXHIB	AXHOH
HIJSEC	HOQRUD	HOQSAK	HOQSEO	HOQSIS	IPAFUD
JELQOJ	JESXEN	JOZZED	KELXAD	KELXEH	KIPGAV
KIPKED	KIPKIH	KIPKON	KIPKUT	KOXPHY11	KULMOU
KULMUA	KUMRER	LIOXPH	LUCJEA	LUGSIP	MEGMEU
MEXTIU	MEXTIU01	MUXHIX	NAMVAC	NAOXAP	NAOXAP11
OHIJEX	OJOCOH	POMQEQ	POMQIU	POMQOA	POMQUG
POMRAN	QAGXAB	QOHXUH	QUYRUA	RELVAJ	RELVEN

RELVIR	RELVOX	RIKJAW	SEMXIU	TANCAO	TANCES
TANCIW	TANCIW01	TANCOC	TANCUI	TANDAP	TANDET
TOYTEJ	TUBNUC	TUBPOY	TUPLUO	UDUROD	UDUWEX
UDUWIB	UKEFEV	UREXPO11	VANVOX	VAYGUY	VAYGUY01
VILFUU	VILGAB	WINSAO	WINSAO01	WUTKUT02	
WUTKUT03	WUTKUT04	WUTLEE	WUXSIT	WUXSOZ	XETSUK
YAFFUJ	YAFGEU	YAFYUD	YAFZAK	YAFZEO	YAFZIS
YAFZOY	YAFZUE	YUHTAW	ZETPAT	ZUWCIG	ZUWCIG01
XOLBAF	KOQROB	NUBDIB	NUBTUD	NUBVAL	

The analysis of crystal structures containing dhp was performed as well.[15,16] It revealed the average length of O−O bond of 1.45(5) Å in 72 fragments of 31 crystal structures.

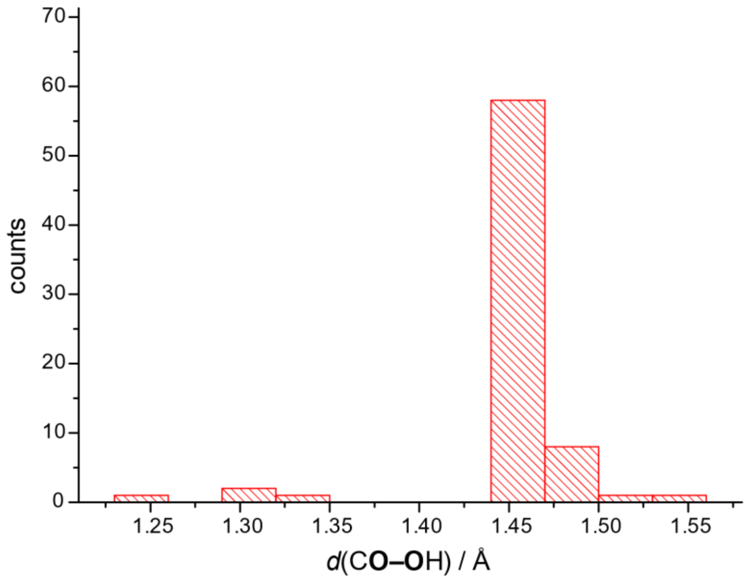

Bin center	Counts
1.215	0
1.245	1
1.275	0
1.305	2
1.335	1
1.365	0
1.395	0
1.425	0
1.455	58
1.485	8
1.515	1
1.545	1
1.575	0
1.605	0

Figure S51. Statistical analysis of C**O−O**H bonds in reported crystals structures in CSD containing DHPs.

List of the crystal structure refcodes in CSD database that contain dhp:

AHEQEL	AHEQIP	BAFGEX	BAFGIB	CURVUI	DPXCYD
DPXCYD01	MALSUN	OCOYOY	OCOYUE	OCOZAL	OCOZEP
REDSAX	REDSEB	REDSIF	REDSOL	REDSUR	REDTAY
REDTEC	REDTEC01	REPWUI	RIHQOR	SEKBOD	VUTFUO
VUTGAV	VUTGEZ	VUTGID	XAHMIC	XAHMIC01	XILRIW
XILROC					

Orientation of the phenyl groups in Ph_3AsO

Analysis of reported crystal structures containing Ph_3AsO in the CSD database was performed.[15,16] The search returned 80 crystal structures with 162 individual Ph_3AsO fragments.

The orientation of the 3 phenyl rings was classified according to the 3 torsion angles defined by O−As−C−C. The compounds were grouped based on the orientation of the tilt of all 3 phenyl rings (in the same or different directions) and the inclination (more or less than 15°).

111 out of 162 fragments have all the phenyl rings tilted in the same direction. In 51 fragments all the torsion angles are bigger than 15°. Both conditions – that the phenyl rings are tilted in the same direction and that this torsion angle is bigger than 15° – resembling a propeller-like structure, are fulfilled in 48 fragments. In only one structure, i.e. BOTMEC, pure Ph_3AsO, all the phenyl rings are virtually parallel to the As=O bond (all torsion angles are smaller than 15°).

Table S20. Analysis of the phenyl group orientation in the crystal structures with Ph_3AsO.

Refcode	Fragment	Torsion angle 1	Torsion angle 2	Torsion angle 3	All phenyl groups tilted in the same direction	All torsion angles > 15°	All torsion angles < 15°
WIXZUC	1	12.666	–10.257	45.024	NO	NO	NO
WIXZUC	2	9.297	31.436	42.308	YES	NO	NO
WIYBAL	1	–0.139	16.272	43.246	NO	NO	NO
WIYBAL	2	12.492	29.805	45.838	YES	NO	NO
AJESAM	1	–50.699	2.044	68.849	NO	NO	NO
BOQYIS	1	–12.563	–32.528	–25.564	YES	NO	NO
BOQYIS	2	28.849	13.797	81.675	YES	NO	NO
BOQYIS	3	51.661	3.877	66.644	YES	NO	NO
BOQYIS	4	3.065	–21.572	–54.24	NO	NO	NO
BOQYOY	1	–25.006	–40.867	–28.319	YES	YES	NO
BOQYOY	2	11.595	14.846	44.567	YES	NO	NO
BOQYOY	3	–84.239	8.18	30.745	NO	NO	NO
BOQYOY	4	–83.743	16.374	15.15	NO	YES	NO
BOQYUE	1	–29.517	–26.473	–22.726	YES	YES	NO
BOQYUE	2	83.343	–0.673	2.949	NO	NO	NO
BOQYUE	3	–59.892	–12.074	–45.851	YES	NO	NO
BOQZAL	1	11.211	15.139	40.818	YES	NO	NO
BOQZAL	2	–88.981	32.595	8.575	NO	NO	NO
BOQZAL	3	18.736	12.143	–83.937	NO	NO	NO
BOQZAL	4	–39.257	–25.163	–28.588	YES	YES	NO
BOQZEP	1	28.128	25.45	23.759	YES	YES	NO
BOQZEP	2	–84.486	0.863	–2.297	NO	NO	NO
BOQZEP	3	12.781	58.503	45.996	YES	NO	NO

BOQZIT	1	–83.811	30.972	7.145	NO	NO	NO
BOQZIT	2	–83.259	14.779	16.768	NO	NO	NO
BOQZIT	3	–41.838	–24.954	–28.569	YES	YES	NO
BOQZIT	4	15.026	11.357	43.964	YES	NO	NO
BOQZOZ	1	–12.332	–59.559	–25.042	YES	NO	NO
BOQZOZ	2	29.572	14.445	81.216	YES	NO	NO
BOQZOZ	3	46.308	3.838	67.54	YES	NO	NO
BOQZOZ	4	1.386	–20.981	–52.767	NO	NO	NO
BOQZUF	1	13.525	41.357	15.276	YES	NO	NO
BOQZUF	2	16.141	–37.299	–75.25	NO	YES	NO
BOQZUF	3	–33.251	–72.114	–33.291	YES	YES	NO
BOQZUF	4	–44.711	–24.003	–72.595	YES	YES	NO
BOQZUF	5	33.926	29.574	18.549	YES	YES	NO
BOQZUF	6	35.91	36.078	14.14	YES	NO	NO
BORBAO	1	44.959	66.386	17.401	YES	YES	NO
BORBAO	2	11.922	29.763	73.173	YES	NO	NO
BORBAO	3	5.26	–16.61	–44.974	NO	NO	NO
BORBAO	4	–44.432	–17.724	–11.605	YES	NO	NO
BORBES	1	45.07	67.28	16.409	YES	YES	NO
BORBES	2	12.145	72.518	30.474	YES	NO	NO
BORBES	3	5.79	–17.072	–45.111	NO	NO	NO
BORBES	4	–44.98	–18.097	–10.476	YES	NO	NO
BORBIW	1	28.156	26.493	22.385	YES	YES	NO
BORBIW	2	–83.19	0.522	–3.237	NO	NO	NO
BORBIW	3	13.063	59.862	45.521	YES	NO	NO
BORBOC	1	16.168	12.366	–83.559	NO	NO	NO
BORBOC	2	7.153	31.527	–88.749	NO	NO	NO
BORBOC	3	45.981	10.343	11.581	YES	NO	NO
BORBOC	4	–23.195	–25.493	–33.378	YES	YES	NO
BORBUI	1	–12.222	–30.193	–25.472	YES	NO	NO
BORBUI	2	28.788	13.658	82.243	YES	NO	NO
BORBUI	3	44.958	3.254	66.094	YES	NO	NO
BORBUI	4	3.978	–20.996	–57.543	NO	NO	NO
**BOTMEC**	**1**	**12.957**	**–0.817**	**–6.376**	**NO**	**NO**	**YES**
CIVNUQ10	1	–83.873	–16.583	12.228	NO	NO	NO
CIVNUQ10	2	59.951	26.914	42.538	YES	YES	NO
DOYDAW	1	–39.716	–24.775	–62.952	YES	YES	NO
EQUFOM	1	35.621	56.362	48.212	YES	YES	NO
FOVKUX	1	37.901	37.058	69	YES	YES	NO
GISRIJ	1	–23.333	–33.157	–39.697	YES	YES	NO
GISRIJ	2	–40.881	–32.481	–22.343	YES	YES	NO
GISRIJ01	1	–15.206	–19.936	–83.644	YES	YES	NO
GISRIJ02	1	52.419	53.694	41.809	YES	YES	NO
HATTIJ	1	–10.768	–55.603	1.416	NO	NO	NO

HIQDOA	1	–83.161	–19.681	23.11	NO	YES	NO
HITWAL	1	86.222	–2.183	–15.071	NO	NO	NO
HITWAL	2	64.634	8.303	17.485	YES	NO	NO
HITWIT	1	2.694	25.1	18.68	YES	NO	NO
HITWIT	2	–11.334	–86.29	–14.543	YES	NO	NO
HITWIT	3	15.324	18.578	27.249	YES	YES	NO
HITWIT	4	–11.546	–83.766	–5.556	YES	NO	NO
HUWDUY	1	–36.274	–68.178	–15.686	YES	YES	NO
HUWDUY	2	24.47	74.557	5.49	YES	NO	NO
HUWFEK	1	75.688	10.382	45.721	YES	NO	NO
HUWFEK	2	–29.397	–28.54	–14.066	YES	NO	NO
HUWFEK	3	40.114	58.396	18.13	YES	YES	NO
IBIYEX	1	–2.907	12.419	87.001	NO	NO	NO
IBIYEX	2	19.845	3.803	–85.695	NO	NO	NO
IBIYIB	1	28.832	75.289	30.412	YES	YES	NO
IBIYIB	2	–5.268	–27.411	–8.62	YES	NO	NO
IBIYIB	3	–10.023	8.98	68.685	NO	NO	NO
IBIYIB	4	72.158	–9.408	23.095	NO	NO	NO
IVAQEB	1	1.268	17.644	69.525	YES	NO	NO
IVODAB	1	5.269	25.458	73.451	YES	NO	NO
IVODAB	2	–26.913	–22.084	–69.759	YES	YES	NO
IVODAB01	1	–63.958	–17.027	–4.683	YES	NO	NO
IVODAB01	2	14.685	–86.864	3.179	NO	NO	NO
IVODEF	1	68.995	30.024	19.423	YES	YES	NO
IVODEF	2	–72.592	–5.422	–22.943	YES	NO	NO
IVOMAK	1	20.651	62.577	7.695	YES	NO	NO
IVOMAK	2	–2.872	–18.364	84.921	NO	NO	NO
JACWEP01	1	–51.273	–51.278	–51.269	YES	YES	NO
JACWEP01	2	54.325	54.355	54.36	YES	YES	NO
JAYCAN	1	54.647	35.208	26.608	YES	YES	NO
KEBYOF	1	55.143	31.334	37.177	YES	YES	NO
KEBYOF	2	44.007	19.211	52.182	YES	YES	NO
KEBYOF	3	5.867	40.424	37.797	YES	NO	NO
KEBYOF	4	35.083	59.507	–1.956	NO	NO	NO
LABLEF	1	–16.786	–53.49	–56.18	YES	YES	NO
LEDDIH	1	59.517	12.277	51.404	YES	NO	NO
LOVNUI	1	87.096	–2.82	8.411	NO	NO	NO
LUWBUA	1	68.492	55.169	39.512	YES	YES	NO
LUWCAH	1	63.556	39.934	42.975	YES	YES	NO
LUWCAH	2	73.948	–4.984	28.899	NO	NO	NO
MEVJOM	1	–10.241	85.406	–12.048	NO	NO	NO
MEVJOM	2	37.219	2.538	78.89	YES	NO	NO
MEVJUS	1	57.053	58.375	10.012	YES	NO	NO
MIFDOU	1	42.279	67.15	18.394	YES	YES	NO

MIFDOU	2	–13.262	–1.641	–84.608	YES	NO	NO
MIJBOW	1	–29.665	–40.013	–37.101	YES	YES	NO
MIJBOW	2	–33.327	–88.132	–11.313	YES	NO	NO
MIJBUC	1	–30.497	–19.897	–39.054	YES	YES	NO
MIJBUC	2	66.008	14.311	21.534	YES	NO	NO
MIJCUD	1	–86.452	–2.928	–5.424	YES	NO	NO
MIJCUD	2	89.17	–8.541	21.077	NO	NO	NO
NAKSID	1	40.299	43.503	45.633	YES	YES	NO
NAKSID	2	–26.755	–7.648	–31.915	YES	NO	NO
NPASOT	1	–70.997	–65.367	–8.563	YES	NO	NO
PAHBUV	1	–55.124	–45.207	–10.118	YES	NO	NO
PASOCT10	1	–37.822	–14.863	–75.002	YES	NO	NO
PASOFP	1	–53.841	–20.232	–21.621	YES	YES	NO
PIXSIB	1	–50.611	4.94	–54.745	NO	NO	NO
PIXSIB	2	–52.149	0.91	–45.971	NO	NO	NO
PIXSIB	3	0.797	–64.329	–51.718	NO	NO	NO
PIXSIB	4	–61.27	–0.8	–55.532	YES	NO	NO
POFNAZ	1	37.643	11.5	70.327	YES	NO	NO
PUVKAV	1	–37.624	–38.605	–69.813	YES	YES	NO
RAZFAB	1	9.962	5.376	–82.134	NO	NO	NO
ROXSIJ	1	–1.679	52.81	7.681	NO	NO	NO
SUYFEZ	1	–83.104	–31.125	–6.274	YES	NO	NO
SUYFEZ	2	21.238	9.167	79.928	YES	NO	NO
TEHYAH	1	3.377	–21.762	–87.617	NO	NO	NO
TPASOM	1	31.644	8.435	55.448	YES	NO	NO
TPASOM03	1	59.349	33.39	6.213	YES	NO	NO
UROMEW	1	16.566	82.272	27.777	YES	YES	NO
UROZOR	1	–79.284	–34.041	–10.697	YES	NO	NO
UROZOR	2	–27.807	–16.209	–84.857	YES	YES	NO
VACRUO	1	–38.285	–21.298	–68.518	YES	YES	NO
VUYREM	1	–39.501	–23.686	–14.249	YES	NO	NO
XOTWEI	1	86.974	26.494	–6.225	NO	NO	NO
XOTWEI	2	–82.72	–40.424	3.335	NO	NO	NO
XOTWEI	3	23.189	87.906	–10.324	NO	NO	NO
XOTWEI	4	78.58	2.088	–32.421	NO	NO	NO
XURFIZ	1	62.488	41.258	34.129	YES	YES	NO
XURFOF	1	–89.241	–2.374	–3.475	YES	NO	NO
XURFOF	2	83.257	–0.177	6.433	NO	NO	NO
XURGEW	1	53.973	17.798	47.484	YES	YES	NO
XURGEW	2	35.198	69.802	6.53	YES	NO	NO
ZEJZAR	1	18.535	54.957	46.557	YES	YES	NO
ZEJZIZ	1	–41.327	–19.591	–66.336	YES	YES	NO
ZIJNIP	1	–69.912	–6.225	–22.867	YES	NO	NO
ZIJNIP	2	71.134	43.368	7.655	YES	NO	NO

ZULDES	1	14.868	48.288	61.036	YES	NO	NO
SOKPOB	1	–4.43	–15.478	85.026	NO	NO	NO
SOKPOB	2	7.163	–79.691	–14.158	NO	NO	NO
SOKYEA	1	–53.43	–34.028	–64.088	YES	YES	NO
SOKYEA	2	–31.452	–47.671	–9.841	YES	NO	NO
SOKYIE	1	5.466	–17.389	87.527	NO	NO	NO
SOKYIE	2	–72.961	–2.856	13.878	NO	NO	NO

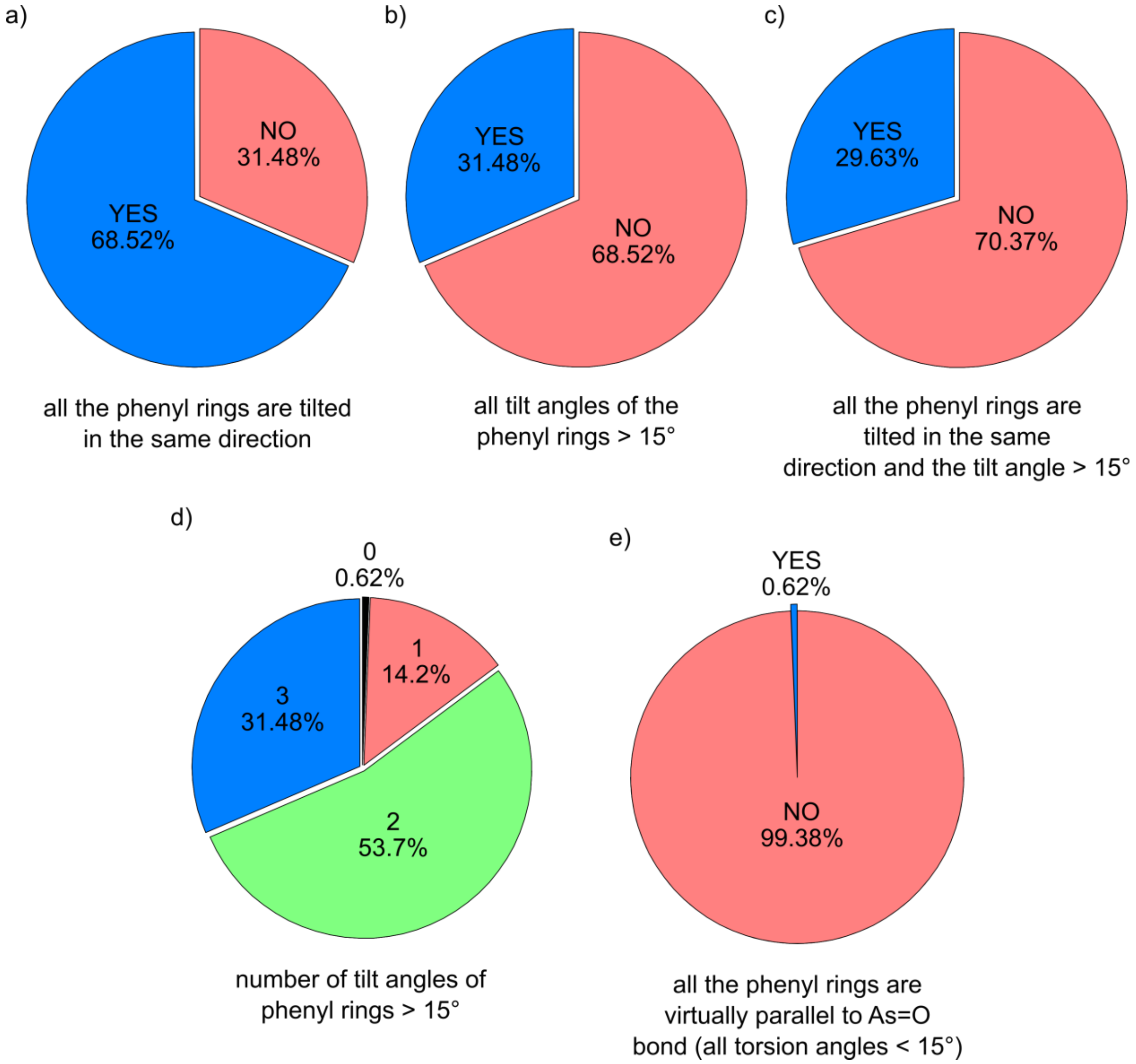

Figure S52. Analysis of the phenyl group orientation in the crystal structures with Ph_3AsO.

COMPUTATIONAL PART

General information

The electronic structures of the optimized geometries were calculated using DFT method PBE0-D3/def2-TZVP as implemented in the *ORCA* software[17,18] (version 6.0.0). Final single point energies were calculated at PBE0-D3/def2-QZVPP level of theory. Solvation energies in acetone and chloroform were computed with the latter method, using SMD implicit solvation model. Analysis of the molecular electrostatic potential was performed using *Multiwfn* software[19] (version 3.8(dev)) to analyze the single point electron densities.[20,21] The images of electrostatic potential were visualized using *GausView 6* software.[22]

Energetics

Table S21. Preliminary computational investigation of hydrogen-bonded adducts.

Compound	E_{tz}	$G_{tz\text{-}corr}$	E_{qz}	$E_{solv\text{-}acetone}$	$G_{s\text{-}acetone}$	$E_{solv\text{-}CHCl3}$	$G_{s\text{-}CHCl3}$
Ph_3PO	–697075.7	148.3	–697111.0	–16.7	–696979.5	–17.4	–696980.2
Ph_3AsO	–1885766.6	146.5	–1885825.2	–19.9	–1885698.6	–20.3	–1885699.0
H_2O_2	–95039.8	3.4	–95047.4	–7.6	–95051.6	–4.9	–95048.9
$(HOO)_2(c\text{-}C_5H_8)$	–311828.8	77.6	–311848.9	–11.2	–311782.4	–7.8	–311779.0
$Ph_3PO{\cdot}H_2O_2$	–792129.2	164.2	–792171.4	–20.6	–792027.9	–18.9	–792026.2
$Ph_3AsO{\cdot}H_2O_2$	–1980822.4	161.6	–1980887.7	–23.2	–1980749.3	–21.4	–1980747.5
$Ph_3PO{\cdot}(HOO)_2(c\text{-}C_5H_8)$	–1008924.3	242.2	–1008979.1	–24.7	–1008761.6	–22.2	–1008759.1
$Ph_3AsO{\cdot}(HOO)_2(c\text{-}C_5H_8)$	–2197619.8	240.1	–2197697.6	–25.4	–2197482.9	–23.3	–2197480.8
$[Ph_3PO{\cdot}H_2O_2]_2$	–1584277.1	345.4	–1584360.7	–36.1	–1584051.4	–33.4	–1584048.7
$[Ph_3AsO{\cdot}H_2O_2]_2$	–3961666.3	340.2	–3961796.0	–39.4	–3961495.3	–37.2	–3961493.1

The values are given in kcal mol^{-1}. $E_{solv} = E_{s\text{-}qz} - E_{qz}$ where E_s is the final SMD corrected single point energy. Final Gibbs free energy of a solvated species is calculated as $G_s = E_{qz} + G_{tz\text{-}corr} + E_{solv}$.

Table S22. Relative stability of the following reactions in acetone and chloroform.

Reaction	$\Delta G_{acetone}$	$K_{acetone}$	ΔG_{CHCl3}	K_{CHCl3}
$Ph_3PO + H_2O_2 \rightleftharpoons Ph_3PO{\cdot}H_2O_2$	3.18	$4.65{\cdot}10^{-3}$	2.90	$7.47{\cdot}10^{-3}$
$Ph_3AsO + H_2O_2 \rightleftharpoons Ph_3AsO{\cdot}H_2O_2$	0.87	0.230	0.37	0.535
$2Ph_3PO + 2H_2O_2 \rightleftharpoons [Ph_3PO{\cdot}H_2O_2]_2$	10.72	$1.37{\cdot}10^{-8}$	9.43	$1.21{\cdot}10^{-7}$
$2Ph_3AsO + 2H_2O_2 \rightleftharpoons [Ph_3AsO{\cdot}H_2O_2]_2$	1.95	0.0371	2.65	0.0114
$Ph_3PO + (HOO)_2(c\text{-}C_5H_8) \rightleftharpoons Ph_3PO{\cdot}(HOO)_2(c\text{-}C_5H_8)$	0.29	0.613	0.04	0.935
$Ph_3AsO + (HOO)_2(c\text{-}C_5H_8) \rightleftharpoons Ph_3AsO{\cdot}(HOO)_2(c\text{-}C_5H_8)$	–1.90	24.7	–2.79	111

The values of Gibbs free energy are given in kcal mol^{-1}. Reaction constant is calculated at 298 K. The Gibbs free energies are not corrected for BSSE error, which is generally below 1 kcal mol^{-1} and slightly disfavors the adduct formation.

Optimized structures

Table S23. Optimized geometry of Ph_3PO.

atom	*x*	*y*	*z*
P	4.9042461	6.9435292	−0.1349361
C	3.1055298	6.9570667	0.0367692
C	2.3592557	6.7992428	−1.1282180
C	2.4548527	7.1041757	1.2575286
C	0.9757010	6.7734209	−1.0682781
H	2.8760777	6.7144681	−2.0774687
C	1.0697371	7.0763051	1.3148792
H	3.0302467	7.2564750	2.1637066
C	0.3308050	6.9074234	0.1534017
H	0.3981547	6.6537012	−1.9774832
H	0.5664096	7.1956861	2.2672710
H	−0.7520539	6.8897775	0.1988708
C	5.5338582	7.8514239	1.2949600
C	5.7532773	9.2164153	1.1285164
C	5.7904333	7.2574375	2.5265661
C	6.2103313	9.9806266	2.1895024
H	5.5771718	9.6636714	0.1567738
C	6.2462756	8.0250563	3.5874545
H	5.6478090	6.1902852	2.6539110
C	6.4527945	9.3862773	3.4200906
H	6.3830617	11.0420783	2.0552062
H	6.4487127	7.5576277	4.5440849
H	6.8129265	9.9847978	4.2489739
C	5.4018611	5.2255000	0.1216745
C	6.5967564	4.8205889	−0.4672196
C	4.6613115	4.3183474	0.8729923
C	7.0526373	3.5242661	−0.2922128
H	7.1503662	5.5280009	−1.0741767
C	5.1208923	3.0220794	1.0481678
H	3.7158244	4.6202302	1.3094083
C	6.3173964	2.6262811	0.4687650
H	7.9814767	3.2116028	−0.7548617
H	4.5391706	2.3171476	1.6306606
H	6.6736110	1.6113576	0.6029603
O	5.3709358	7.4959441	−1.4278006

Table S24. Optimized geometry of Ph_3AsO.

atom	*x*	*y*	*z*
As	4.8966691	7.0648198	−0.0918005
C	2.9776442	6.9601724	0.0732790
C	2.2831630	6.0644382	−0.7325506
C	2.2863309	7.7985443	0.9379963
C	0.9007265	6.0046483	−0.6668568
H	2.8217494	5.4119146	−1.4113325
C	0.9018526	7.7360192	1.0013528
H	2.8263433	8.4995622	1.5645745
C	0.2104118	6.8395634	0.2010340
H	0.3603101	5.3059762	−1.2948566
H	0.3631660	8.3902811	1.6769486
H	−0.8712015	6.7912175	0.2520369
C	5.5102079	7.9421064	1.5105794
C	6.2346683	9.1125023	1.3333470
C	5.2685836	7.4480045	2.7864807
C	6.7158227	9.7952490	2.4412025
H	6.4134223	9.4678583	0.3237336
C	5.7516685	8.1329476	3.8900363
H	4.7064206	6.5309823	2.9253140
C	6.4739511	9.3061814	3.7161810
H	7.2819215	10.7098953	2.3082195
H	5.5671573	7.7507183	4.8873177
H	6.8512273	9.8397795	4.5810355
C	5.4907408	5.2339953	−0.0148566
C	6.5585153	4.8868405	−0.8314920
C	4.9026809	4.2882982	0.8159318
C	7.0469819	3.5891993	−0.8067039
H	6.9844378	5.6386786	−1.4876821
C	5.3953257	2.9927227	0.8385467
H	4.0497154	4.5520904	1.4322214
C	6.4684574	2.6454094	0.0296402
H	7.8787765	3.3129206	−1.4442808
H	4.9380020	2.2513397	1.4835188
H	6.8508469	1.6312670	0.0468918
O	5.4611569	7.8221715	−1.4245673

Table S25. Optimized geometry of H_2O_2.

atom	*x*	*y*	*z*
O	−0.1947532	5.8547117	1.5997986
H	0.6181793	5.8634334	1.0802634
O	−0.5343054	7.2394127	1.5997985
H	−1.3472372	7.2306917	1.0802622

Table S26. Optimized geometry of $(HOO)_2(c\text{-}C_5H_8)$.

atom	*x*	*y*	*z*
O	6.5796141	9.2649702	3.8186803
H	6.3265856	10.1950837	3.7028223
O	7.9079870	9.2420907	3.3030628
O	7.5076779	11.1177644	2.0667447
O	8.4525608	11.8562868	2.8363577
H	8.4887315	11.3499477	3.6639261
C	7.9115184	9.7627646	1.9902960
C	6.9368458	9.0876873	1.0210889
H	6.2324043	8.4596867	1.5635183
H	6.3670488	9.8665663	0.5089291
C	7.8257069	8.3297669	0.0360629
H	7.3487809	8.1914289	−0.9353227
H	8.0595763	7.3370251	0.4323448
C	9.0943005	9.1716241	−0.0212270
H	9.9476086	8.6469089	−0.4535597
H	8.9201455	10.0707868	−0.6200565
C	9.3203514	9.5553855	1.4372379
H	9.9311830	10.4442635	1.5830672
H	9.7819486	8.7284190	1.9826079

Table S27. Optimized geometry of $Ph_3PO{\cdot}H_2O_2$.

atom	*x*	*y*	*z*
P	4.8287307	6.9089843	−0.3820735
C	3.0406179	6.7071194	−0.3093100
C	2.4319885	5.4866718	−0.0362037
C	2.2581650	7.8362660	−0.5540847
C	1.0484226	5.3939876	−0.0015663
H	3.0373440	4.6059887	0.1425998
C	0.8780518	7.7362203	−0.5186700
H	2.7360833	8.7868666	−0.7692635
C	0.2728898	6.5174402	−0.2414256
H	0.5771388	4.4409654	0.2082329
H	0.2718254	8.6135739	−0.7110718
H	−0.8083013	6.4435529	−0.2162374
C	5.2767516	7.8210821	1.1086592
C	6.4611000	8.5546309	1.0843615
C	4.4866275	7.8218120	2.2533046
C	6.8477556	9.2823153	2.1979710
H	7.0586955	8.5720977	0.1800121
C	4.8809530	8.5448515	3.3690516
H	3.5512656	7.2736525	2.2654245
C	6.0598023	9.2745630	3.3414668
H	7.7646843	9.8592963	2.1727067
H	4.2598955	8.5480236	4.2570367
H	6.3627359	9.8465006	4.2108256
C	5.5427087	5.2650741	−0.2019568
C	5.9489026	4.6134041	−1.3625863
C	5.6850577	4.6372197	1.0318501
C	6.4819577	3.3363485	−1.2882502
H	5.8544362	5.1235382	−2.3142103
C	6.2172995	3.3600120	1.1021976
H	5.3900381	5.1512021	1.9398174
C	6.6128148	2.7090293	−0.0578854
H	6.8003378	2.8313130	−2.1925938
H	6.3307600	2.8745311	2.0643454
H	7.0324701	1.7112835	−0.0010597
O	5.3093924	7.5797747	−1.6233498
O	5.3264140	10.2854854	−1.7239502
O	4.3273174	10.4879066	−0.7279640
H	4.8587050	10.5662447	0.0745271
H	5.3271603	9.3025444	−1.7968138

Table S28. Optimized geometry of $Ph_3AsO \cdot H_2O_2$.

atom	*x*	*y*	*z*
As	4.8407768	6.9177404	−0.3994574
C	2.9298881	6.7316242	−0.3222141
C	2.3378419	5.4966273	−0.0912490
C	2.1497907	7.8671969	−0.5194446
C	0.9546942	5.3968013	−0.0489952
H	2.9499471	4.6128252	0.0481743
C	0.7692617	7.7569957	−0.4766249
H	2.6237668	8.8255271	−0.7071037
C	0.1728519	6.5257949	−0.2402530
H	0.4887397	4.4346230	0.1286239
H	0.1566935	8.6373063	−0.6323058
H	−0.9077109	6.4457563	−0.2092485
C	5.3435928	7.9035691	1.1724533
C	6.5153855	8.6495098	1.1220344
C	4.5623086	7.8925151	2.3198339
C	6.9049253	9.3855385	2.2302105
H	7.0988812	8.6715875	0.2079445
C	4.9609581	8.6257955	3.4281762
H	3.6334587	7.3327208	2.3435466
C	6.1303595	9.3700386	3.3827777
H	7.8135291	9.9749797	2.1932540
H	4.3517196	8.6240267	4.3243888
H	6.4366381	9.9480203	4.2469120
C	5.5804952	5.1551193	−0.2163966
C	6.0991494	4.5615485	−1.3593222
C	5.6086194	4.4890205	1.0024832
C	6.6377869	3.2859598	−1.2815775
H	6.0881990	5.1118425	−2.2938035
C	6.1476852	3.2140158	1.0738260
H	5.2218978	4.9641630	1.8977829
C	6.6587113	2.6133484	−0.0684757
H	7.0458738	2.8177719	−2.1695944
H	6.1747602	2.6909611	2.0225380
H	7.0820973	1.6173060	−0.0102541
O	5.4095853	7.6225746	−1.7724786
O	5.2394477	10.2774742	−1.7824236
O	4.2761315	10.4016986	−0.7377612
H	4.8352881	10.5728747	0.0298986
H	5.3049695	9.2885747	−1.8620114

Table S29. Optimized geometry of $Ph_3PO{\cdot}(HOO)_2(c\text{-}C_5H_8)$.

atom	*x*	*y*	*z*
O	6.6434977	10.9369930	5.3233868
O	6.7985588	8.6811724	3.7374768
H	6.6043188	9.4376465	4.3290449
O	8.0628719	9.0649900	3.2107094
O	7.1361314	10.9067798	2.1185949
O	7.8021628	11.8122682	2.9899107
H	7.4299573	11.5443787	3.8566084
C	7.8928915	9.7316837	1.9784418
C	7.1544186	8.8982305	0.9243408
H	6.5980973	8.0924585	1.3995766
H	6.4383421	9.5476768	0.4159226
C	8.2430888	8.4389640	−0.0416842
H	7.8551225	8.2044870	−1.0348819
H	8.7293245	7.5366334	0.3431395
C	9.2317592	9.5990792	−0.0295379
H	10.2052966	9.3505876	−0.4568679
H	8.8236907	10.4381383	−0.6019775
C	9.3109891	9.9688864	1.4481320
H	9.6246798	10.9917648	1.6483087
H	9.9933576	9.2948625	1.9721218
P	7.2102812	11.2062239	6.6844774
C	5.9111665	11.4697073	7.9002627
C	4.7325400	10.7419827	7.7555335
C	6.0574617	12.3476585	8.9693194
C	3.7133038	10.8864246	8.6816244
H	4.6166046	10.0768821	6.9076087
C	5.0355326	12.4873922	9.8955181
H	6.9649174	12.9318185	9.0710825
C	3.8661209	11.7556771	9.7528678
H	2.7953216	10.3227504	8.5650237
H	5.1497217	13.1750146	10.7251395
H	3.0660822	11.8688574	10.4752354
C	8.2404914	12.6800616	6.6985587
C	9.3464915	12.8009439	7.5344759
C	7.8956685	13.7263478	5.8460765
C	10.0953248	13.9669605	7.5265701
H	9.6302192	11.9775992	8.1800069
C	8.6516058	14.8864386	5.8366252
H	7.0480434	13.6213200	5.1786528
C	9.7471903	15.0085441	6.6792788
H	10.9589591	14.0578424	8.1746491
H	8.3883542	15.6935858	5.1637004
H	10.3389620	15.9165009	6.6679954
C	8.2300207	9.8606889	7.2970727
C	9.0203830	9.1780935	6.3742762
C	8.2652225	9.5079755	8.6432108
C	9.8456094	8.1530347	6.8057118
H	8.9747623	9.4314310	5.3207504
C	9.0977950	8.4848767	9.0679836
H	7.6342264	10.0250669	9.3569640
C	9.8883111	7.8097337	8.1494729
H	10.4509639	7.6157208	6.0855853
H	9.1220666	8.2084794	10.1154626
H	10.5342038	7.0051779	8.4818460

Table S30. Optimized geometry of $Ph_3AsO\cdot(HOO)_2(c\text{-}C_5H_8)$.

atom	*x*	*y*	*z*
O	6.0244760	9.8060963	5.4685555
O	7.6009171	8.4413461	3.7454122
H	6.8767478	8.7579573	4.3367137
O	8.2432043	9.6823711	3.4734729
O	6.3789941	10.3715852	2.2479365
O	5.9936348	11.3324645	3.2225337
H	5.9043048	10.7752127	4.0329681
C	7.7671486	10.2123839	2.2480604
C	8.0966659	9.3062139	1.0626426
H	8.0828987	8.2599768	1.3631601
H	7.3349957	9.4533658	0.2929084
C	9.4468822	9.8207627	0.5807086
H	9.6868239	9.5018442	−0.4354081
H	10.2394868	9.4525998	1.2399227
C	9.3072821	11.3325419	0.7280325
H	10.2616988	11.8625203	0.7002325
H	8.6938845	11.7225363	−0.0903081
C	8.5742337	11.5051796	2.0595745
H	7.9073075	12.3645434	2.0938874
H	9.2771788	11.6019871	2.8897686
As	6.9724697	10.5027023	6.6374307
C	5.8356564	11.3612806	7.9187178
C	4.5140099	10.9421640	7.9948122
C	6.3082823	12.3737581	8.7450568
C	3.6629805	11.5353227	8.9145411
H	4.1615573	10.1686845	7.3215100
C	5.4528434	12.9602898	9.6646028
H	7.3349851	12.7140462	8.6620613
C	4.1331317	12.5390020	9.7493280
H	2.6291145	11.2168810	8.9760168
H	5.8142994	13.7524067	10.3097374
H	3.4647931	13.0023529	10.4658167
C	8.1627329	11.8644174	5.9970045
C	9.5380327	11.7202777	6.1170163
C	7.6207771	12.9938567	5.3948700
C	10.3755607	12.7133940	5.6318635
H	9.9565271	10.8339670	6.5790777
C	8.4621646	13.9814954	4.9123634
H	6.5477971	13.0990499	5.2858376
C	9.8377213	13.8411548	5.0306932
H	11.4497844	12.6015225	5.7190624
H	8.0425908	14.8580098	4.4335992
H	10.4940863	14.6138630	4.6474329
C	8.0478018	9.2178537	7.5721161
C	8.7248661	8.2454047	6.8423771
C	8.1588168	9.2703255	8.9560738
C	9.5196218	7.3276498	7.5118380
H	8.6256434	8.1976835	5.7624011
C	8.9558612	8.3472978	9.6156737
H	7.6179130	10.0202335	9.5218878
C	9.6366801	7.3787086	8.8934875
H	10.0463396	6.5666228	6.9482567
H	9.0405827	8.3828598	10.6953394
H	10.2576954	6.6564671	9.4107067

Table S31. Optimized geometry of $[Ph_3PO \cdot H_2O_2]_2$.

atom	*x*	*y*	*z*
P	−2.2152914	10.4819608	6.9114299
O	−2.2886808	9.5646587	5.7281643
O	−4.7760108	7.5406019	5.4280291
O	−3.5548671	7.1656831	6.0627168
C	−1.7158868	9.5759240	8.3818035
C	−1.0131266	11.7991615	6.6690201
C	−3.7841224	11.2741460	7.2680828
C	−6.1797265	11.1555497	7.2281956
C	−0.1016182	13.4778979	5.2097103
C	−4.9501341	10.5857485	6.9386521
C	−1.0062243	12.4550883	5.4379090
C	−0.1106794	12.1725528	7.6593190
C	0.7935459	13.8532015	6.2024765
C	−6.2492153	12.3984153	7.8407139
C	−2.1041017	9.9481288	9.6645751
C	−0.9067239	8.4583743	8.1956727
C	0.7901008	13.2007903	7.4256233
C	−3.8534974	12.5271377	7.8716485
C	−5.0873543	13.0862567	8.1608464
C	−0.8584046	8.1052341	10.5701454
C	−1.6711439	9.2139927	10.7573401
C	−0.4803268	7.7255100	9.2904156
H	−0.0953146	13.9819856	4.2506315
H	−0.1051632	11.6528581	8.6103754
H	−2.7582221	10.8007828	9.8075511
H	−1.6952974	12.1587253	4.6533606
H	−7.2146755	12.8393108	8.0608597
H	−4.9001955	9.6196312	6.4448782
H	1.4997189	14.6548857	6.0191170
H	−7.0874045	10.6252641	6.9659245
H	−2.9441429	13.0724044	8.0993298
H	1.4933483	13.4878376	8.1986622
H	0.1410600	6.8502060	9.1418649
H	−0.6273181	8.1593137	7.1921091
H	−5.1424403	14.0637815	8.6253151
H	−0.5279646	7.5279056	11.4259875
H	−3.0220067	7.9837896	5.9611761
H	−4.5201037	7.4777021	4.4830641
H	−1.9787136	9.5007744	11.7559652
P	−2.4567210	6.3587227	2.7691093
O	−3.5772437	7.3331757	2.9705414
O	−2.4451734	10.3686175	3.1206131
O	−3.7733239	10.0783252	2.6914412
C	−1.8581007	6.4207001	1.0735897
C	−2.9669707	4.6636813	3.0945231
C	−1.0045290	6.6696610	3.7895475
C	0.7110109	8.2190794	4.4535288
C	−4.0149431	3.1059537	4.5967924
C	−0.4086592	7.9262712	3.6946662
C	−3.5869831	4.3925381	4.3154490
C	−2.7871391	3.6430211	2.1663202
C	−3.8321814	2.0892238	3.6692481
C	1.2469272	7.2610689	5.3030435
C	−0.6005143	5.9384331	0.7206108
C	−2.6917880	6.9627347	0.1015008
C	−3.2199249	2.3577463	2.4549929
C	−0.4666430	5.7118661	4.6433084
C	0.6576783	6.0091571	5.3986662
C	−1.0237547	6.5215743	−1.5693270
C	−0.1869760	5.9850680	−0.6000190
C	−2.2719696	7.0134582	−1.2185794
H	−4.4972452	2.8989557	5.5447834
H	−2.3162895	3.8528305	1.2136688
H	0.0584366	5.5372196	1.4827454
H	−3.7235147	5.1867002	5.0420850
H	2.1238715	7.4937798	5.8968354
H	−0.8388540	8.6855761	3.0502446
H	−4.1724176	1.0844819	3.8929372
H	1.1596973	9.2032600	4.3880647
H	−0.9280723	4.7354005	4.7220680
H	−3.0820475	1.5669311	1.7270524
H	−2.9191600	7.4442571	−1.9733124
H	−3.6590967	7.3551601	0.3919325
H	1.0719998	5.2604236	6.0637584
H	−0.6958802	6.5646200	−2.6015611
H	−3.7492836	9.1017068	2.6482877
H	−2.4666410	10.1022851	4.0640483
H	0.7931448	5.6121611	−0.8730675

Table S32. Optimized geometry of $[Ph_3AsO{\cdot}H_2O_2]_2$.

atom	*x*	*y*	*z*
As	−1.9606753	10.5207499	7.5156557
O	−1.1964134	9.2580499	6.7638570
O	−3.3346790	6.9136001	6.0543835
O	−1.9870656	6.6714073	6.4557699
C	−2.0316536	10.1614300	9.3976912
C	−1.0189054	12.1744653	7.2758153
C	−3.7574966	10.8547079	6.9395985
C	−5.8944342	10.0164161	6.2668685
C	−0.1884022	13.8568808	5.7857500
C	−4.5822722	9.7769281	6.6434456
C	−0.8775158	12.6712207	5.9829217
C	−0.4765970	12.8522516	8.3598571
C	0.3522120	14.5400217	6.8665548
C	−6.3773296	11.3152874	6.1939466
C	−2.8645865	10.8847004	10.2423476
C	−1.2300854	9.1425034	9.8934277
C	0.2099252	14.0394517	8.1518116
C	−4.2324871	12.1585022	6.8664067
C	−5.5483963	12.3860647	6.4934619
C	−2.0779540	9.5796118	12.0985636
C	−2.8829065	10.5934960	11.5969747
C	−1.2563647	8.8531252	11.2494513
H	−0.0699291	14.2448123	4.7810163
H	−0.5861501	12.4561408	9.3630543
H	−3.5053786	11.6645966	9.8448859
H	−1.2888372	12.1303132	5.1374878
H	−7.4042618	11.4950281	5.8967286
H	−4.1994746	8.7608130	6.6813036
H	0.8905098	15.4670073	6.7051540
H	−6.5398755	9.1802663	6.0245662
H	−3.5780218	12.9950852	7.0833606
H	0.6356186	14.5710599	8.9948014
H	−0.6379682	8.0546560	11.6421554
H	−0.6064770	8.5773991	9.2099925
H	−5.9225301	13.4011243	6.4290579
H	−2.0983254	9.3498757	13.1576403
H	−1.6632019	7.5872541	6.6181881
H	−3.2092104	7.1695633	5.1068270
H	−3.5320921	11.1520093	12.2610361
As	−2.0717671	6.3300187	2.6985266
O	−2.7456219	7.5931934	3.5311683
O	−0.5623735	9.9085428	4.2484118
O	−1.9074130	10.1728790	3.8509920
C	−2.2651321	6.6608771	0.8191262
C	−2.9386812	4.6631346	3.0819220
C	−0.2095828	6.0330439	3.0357964
C	1.9668711	6.9031815	3.5104978
C	−3.5049350	2.9750352	4.6839531
C	0.6115688	7.1182248	3.3126075
C	−2.8760711	4.1722848	4.3830732
C	−3.6237254	3.9696887	2.0927076
C	−4.1868421	2.2754296	3.6977831
C	2.4941760	5.6226479	3.4266239
C	−1.5209357	5.9619773	−0.1231097
C	−3.1744592	7.6327444	0.4247981
C	−4.2479583	2.7708069	2.4040569
C	0.3095422	4.7457632	2.9569908
C	1.6671820	4.5434845	3.1510153
C	−2.6128012	7.1964896	−1.8702393
C	−1.6995553	6.2302870	−1.4708780
C	−3.3461977	7.8987831	−0.9253474
H	−3.4664166	2.5918596	5.6966795
H	−3.6723363	4.3619562	1.0831754
H	−0.7949828	5.2195467	0.1906815
H	−2.3557017	4.7263591	5.1572762
H	3.5546828	5.4625722	3.5844446
H	0.1982268	8.1190467	3.4033115
H	−4.6767861	1.3394324	3.9406741
H	2.6111052	7.7445312	3.7375041
H	−0.3423196	3.9011254	2.7629621
H	−4.7840183	2.2261844	1.6356396
H	−4.0502690	8.6603711	−1.2391979
H	−3.7271272	8.1807104	1.1796854
H	2.0763647	3.5414954	3.0959802
H	−2.7469877	7.4080818	−2.9247745
H	−2.2453201	9.2642106	3.6792497
H	−0.6916089	9.6444167	5.1936734
H	−1.1197039	5.6907054	−2.2104578

SUPPORTING REFERENCES

(1) Starkl Renar, K.; Pečar, S.; Iskra, J., Activation of aqueous hydrogen peroxide for non-catalyzed dihydroperoxidation of ketones by azeotropic removal of water. *Org. Biomol. Chem.* **2015**, *13*, 9369–9372.

(2) Weber, M.; Hellriegel, C.; Rueck, A.; Wuethrich, J.; Jenks, P.; Obkircher, M., Method development in quantitative NMR towards metrologically traceable organic certified reference materials used as ^{31}P qNMR standards. *Anal. Bioanal. Chem.* **2015**, *407*, 3115–3123.

(3) Oxley, J. C.; Brady, J.; Wilson, S. A.; Smith, J. L., The risk of mixing dilute hydrogen peroxide and acetone solutions. *J. Chem. Health Saf.* **2012**, *19*, 27–33.

(4) Žmitek, K.; Zupan, M.; Stavber, S.; Iskra, J., Iodine as a Catalyst for Efficient Conversion of Ketones to *gem*-Dihydroperoxides by Aqueous Hydrogen Peroxide. *Org. Lett.* **2006**, *8*, 2491–2494.

(5) Ahn, S. H.; Cluff, K. J.; Bhuvanesh, N.; Blümel, J., Hydrogen Peroxide and Di(hydroperoxy)propane Adducts of Phosphine Oxides as Stoichiometric and Soluble Oxidizing Agents. *Angew. Chem. Int. Ed.* **2015**, *54*, 13341–13345.

(6) Arp, F. F.; Bhuvanesh, N.; Blümel, J., Hydrogen peroxide adducts of triarylphosphine oxides. *Dalton Trans.* **2019**, *48*, 14312–14325.

(7) Rigaku OD, *CrysAlis PRO*. Rigaku Corporation: Wrocław, Poland, **2024**.

(8) Sheldrick, G., Crystal structure refinement with *SHELXL*. *Acta Crystallogr. C* **2015**, *71*, 3–8.

(9) Dolomanov, O. V.; Bourhis, L. J.; Gildea, R. J.; Howard, J. A. K.; Puschmann, H., *OLEX2*: a complete structure solution, refinement and analysis program. *J. Appl. Crystallogr.* **2009**, *42*, 339–341.

(10) Brandenburg, K. Diamond – Crystal and Molecular Structure Visualization. Crystal Impact GbR: Bonn, Germany, **2005**.

(11) Shao, M.; Jin, X.; Tang, Y.; Huang, Q.; Huang, Y., Remarks on the structural evidence of high reactivity of arsonium ylides bearing with an electron-withdrawing substituent. *Tetrahedron Lett.* **1982**, *23*, 5343–5346.

(12) Savariault, J. M.; Lehmann, M. S., Experimental determination of the deformation electron density in hydrogen peroxide by combination of x-ray and neutron diffraction measurements. *J. Am. Chem. Soc.* **1980**, *102*, 1298–1303.

(13) Busing, W. R.; Levy, H. A., Crystal and Molecular Structure of Hydrogen Peroxide: A Neutron-Diffraction Study. *J. Chem. Phys.* **1965**, *42*, 3054–3059.

(14) Abrahams, S. C.; Collin, R. L.; Lipscomb, W. N., The crystal structure of hydrogen peroxide. *Acta Crystallogr.* **1951**, *4*, 15–20.

(15) Bruno, I. J.; Cole, J. C.; Edgington, P. R.; Kessler, M.; Macrae, C. F.; McCabe, P.; Pearson, J.; Taylor, R., New software for searching the Cambridge Structural Database and visualizing crystal structures. *Acta Crystallogr. B* **2002**, *58*, 389–397.

(16) Groom, C. R.; Bruno, I. J.; Lightfoot, M. P.; Ward, S. C., The Cambridge Structural Database. *Acta Crystallogr. B* **2016**, *72*, 171–179.

(17) Neese, F., The ORCA program system. *WIREs Comput. Mol. Sci.* **2012**, *2*, 73–78.

(18) Neese, F., Software update: The ORCA program system—Version 5.0. *WIREs Comput. Mol. Sci.* **2022**, *12*, e1606.

(19) Lu, T.; Chen, F., Multiwfn: A multifunctional wavefunction analyzer. *J. Comput. Chem.* **2012**, *33*, 580–592.

(20) Zhang, J.; Lu, T., Efficient evaluation of electrostatic potential with computerized optimized code. *Phys. Chem. Chem. Phys.* **2021**, *23*, 20323–20328.

(21) Lu, T.; Chen, F., Quantitative analysis of molecular surface based on improved Marching Tetrahedra algorithm. *J. Mol. Graph. Model.* **2012**, *38*, 314–323.

(22) Dennington, R.; Keith, T. A.; Millam, J. M. GaussView, Version 6. Semichem Inc., Shawnee Mission, KS, **2016**.
